# T-Cell Cytokine Response in *Salmonella* Typhimurium-Vaccinated versus Infected Pigs

**DOI:** 10.3390/vaccines9080845

**Published:** 2021-08-02

**Authors:** Selma Schmidt, Heinrich Kreutzmann, Maria Stadler, Kerstin H. Mair, Melissa R. Stas, Michaela Koch, Eleni Vatzia, Sophie Dürlinger, Christian Knecht, Joachim Spergser, Marlies Dolezal, Sven Springer, Tobias Theuß, Vicky Fachinger, Andrea Ladinig, Armin Saalmüller, Wilhelm Gerner

**Affiliations:** 1 Institute of Immunology, Department of Pathobiology, University of Veterinary Medicine, 1210 Vienna, Austria; selma.schmidt@pirbright.ac.uk (S.S.); maria.stadler@vetmeduni.ac.at (M.S.); kerstin.mair@vetmeduni.ac.at (K.H.M.); eleni.vatzia@pirbright.ac.uk (E.V.); armin.saalmueller@vetmeduni.ac.at (A.S.); 2 University Clinic for Swine, Department for Farm Animals and Veterinary Public Health, University of Veterinary Medicine, 1210 Vienna, Austria; heinrich.kreutzmann@vetmeduni.ac.at (H.K.); melissa.stas@vetmeduni.ac.at (M.R.S.); michaela.koch@vetmeduni.ac.at (M.K.); sophie.duerlinger@vetmeduni.ac.at (S.D.); christian.knecht@vetmeduni.ac.at (C.K.); andrea.ladinig@vetmeduni.ac.at (A.L.); 3 Institute of Microbiology, Department of Pathobiology, University of Veterinary Medicine, 1210 Vienna, Austria; joachim.spergser@vetmeduni.ac.at; 4 Platform for Bioinformatics and Biostatistics, Department of Biomedical Sciences, University of Veterinary Medicine, 1210 Vienna, Austria; marlies.dolezal@vetmeduni.ac.at; 5 Ceva Innovation Center GmbH, 06861 Dessau-Roßlau, Germany; sven.springer@ceva.com (S.S.); tobias.theuss@ceva.com (T.T.); vicky.fachinger@ceva.com (V.F.)

**Keywords:** *Salmonella* Typhimurium, pig, multifunctional T cells, interferon-γ, tumor necrosis factor-α, Interleukin-17A, lamina propria lymphocytes

## Abstract

Vaccination with the live attenuated vaccine Salmoporc is an effective measure to control *Salmonella* Typhimurium (STM) in affected swine populations. However, the cellular immune response evoked by the Salmoporc vaccine including differences in vaccinated pigs versus non-vaccinated pigs upon STM infection have not been characterized yet. To investigate this, tissue-derived porcine lymphocytes from different treatment groups (vaccination-only, vaccination and infection, infection-only, untreated controls) were stimulated in vitro with heat-inactivated STM and abundances of IFN-γ, TNF-α and/or IL-17A-producing T-cell subsets were compared across organs and treatment groups. Overall, our results show the induction of a strong CD4^+^ T-cell response after STM infection, both locally and systemically. Low-level induction of STM-specific cytokine-producing CD4^+^ T cells, notably for the IFN-γ/TNF-α co-producing phenotype, was detected after vaccination-only. Numerous significant contrasts in cytokine-producing T-cell phenotypes were observed after infection in vaccinated and infected versus infected-only animals. These results suggest that vaccine-induced STM-specific cytokine-producing CD4^+^ T cells contribute to local immunity in the gut and may limit the spread of STM to lymph nodes and systemic organs. Hence, our study provides insights into the underlying immune mechanisms that account for the efficacy of the Salmoporc vaccine.

## 1. Introduction

*Salmonella* Typhimurium (STM), a Gram-negative facultative intracellular bacterium within the family of the *Enterobacteriaceae*, can infect a broad range of host species. Nontyphoidal *Salmonella* (NTS) serovars such as STM are among the most frequent causes of gastrointestinal infections in humans [1,2] but invasive strains can also lead to more severe manifestations such as septicemia and meningitis in children and immunocompromised individuals [3,4]. In industrialized countries, the consumption of contaminated food of animal origin poses the greatest risk of STM transmission to humans; this applies in particular to products originating from pigs [5,6,7]. Although STM infection in weaner pigs can manifest as enterocolitis with symptoms such as fever and diarrhea, pigs more often act as subclinical carriers [8,9] making it harder to recognize and eradicate the disease on the farm level. Considering the rise of resistance to antimicrobials observed in many *Salmonella* strains [10], alternative measures such as vaccination are promising strategies to control *Salmonella* in affected swine herds [11]. Indeed, the live attenuated histidine–adenine auxotrophic vaccine Salmoporc (Ceva Santé Animale, Libourne, France), which is commercially available for the use in pigs in Europe, was confirmed in its effect of reducing clinical signs, tissue colonization and shedding in numerous studies [12,13,14,15,16,17,18].

Furthermore, vaccination with Salmoporc was shown to induce *Salmonella*-specific IgM, IgG and IgA antibodies [15,19] as well as differentiation of T follicular helper (Tfh) cells [20] in immunized pigs. The exact role of the humoral immune response in controlling STM infections has been subject to much debate and limited data are available on this in pigs. B-cell deficient mice were able to resolve infection with attenuated STM strains, but succumbed to infection with virulent strains [21,22]. Moreover, antibodies were shown to be protective in NTS bacteremia in African children [23]. 

When it comes to bacterial clearance of STM infection, however, there is consensus that a CD4^+^ T-cell response is vital [24,25]. Especially Th1 cells with their capacity for interferon-γ (IFN-γ) production, leading to an activation of antimicrobial macrophages, are essential for the resolution of infection in the mouse model [26,27,28]. In line with these findings, in HIV-infected humans with their reduction of CD4^+^ T cells otherwise non-invasive nontyphoidal *Salmonella* can cause systemic infection and disease [29]. Interleukin-17A (IL-17A)-producing Th17 cells assist in bacterial clearance through recruitment of neutrophils [30]. This corresponds to previous studies in pigs where mRNA level expression of cytokines in STM-infected piglets also suggested a T helper (Th) 1-driven response [31,32]. 

In a previous experiment, we could demonstrate the induction of multifunctional STM-specific CD4^+^ T cells in pigs that received a two-time Salmoporc immunization with a subsequent STM challenge [33]. Since all pigs in that study were both vaccinated and infected it remained unclear to what extent the vaccination influenced the CD4^+^ T-cell response measured after STM challenge infection. In humans, immunization with a live attenuated typhoid vaccine was shown to elicit *Salmonella* Typhi (*S*. Typhi)-specific multifunctional CD4^+^ T cells in the blood that predominantly produced IFN-γ and tumor necrosis factor-α (TNF-α) [34] as well as mucosal immunity at the site of infection driven by CD4^+^ and CD8^+^ T cells [35,36]. Although *S*. Typhi as a typhoidal serovar differs from NTS in many aspects [37], it is conceivable that the Salmoporc vaccine might have similar effects on the porcine immune system.

With the aim of addressing these knowledge gaps, we characterized the STM-specific response of CD4^+^, CD8^+^ and CD4^−^CD8β^−^ porcine T cells (the latter mainly representing γδ T cells) that were isolated from systemic organs such as blood and spleen as well as from local sites of immunity such as mesenteric lymph nodes and the gut. Frequencies of STM-specific IFN-γ, TNF-α and/or IL-17A-producing T cells measured in these tissues at 7 and 21 days post Salmoporc vaccination and/or STM infection were then compared between only vaccinated (VAC), vaccinated and infected (V+I), only infected (INF) and untreated control (CON) pigs.

## 2. Materials and Methods

### 2.1. Animals

Sera from sows (Large White × Landrace) at a university-owned pig farm in Lower Austria were tested by the IDEXX Swine *Salmonella* Ab test (IDEXX Europe, Hoofddorp, The Netherlands) prior to the study. The five sows with the lowest sample to positive control (S/P) ratios (S/P ratios: 0.295–0.473, cut-off for positivity: 1.0) were selected and 44 female and male castrated pigs (Large White × Landrace × Pietrain) from those sows were included in the study at the age of four weeks. All piglets underwent routine vaccinations against PCV-2 (Ingelvac CircoFLEX^®^, Boehringer-Ingelheim, Ingelheim am Rhein, Germany) at three weeks of age and *Mycoplasma hyopneumoniae* (M^+^PAC^®^, MSD Animal Health, Kenilworth, NJ, USA) in the first and third week of life at the farm. The *Salmonella*-free status of the piglets at the start of the study was validated by serological testing for *Salmonella*-specific antibodies by the IDEXX Swine *Salmonella* Ab test (IDEXX Europe) and by microbiological testing of fecal samples collected on three consecutive days after arrival (study days (SD) −11, −10, −9). Antibody S/P ratios were below the positivity cut-off for piglets (0.25). Additionally, no *Salmonella* were detectable in the fecal samples of the piglets at the stated time points.

### 2.2. Vaccination and Infection Experiment

**Figure 1:**
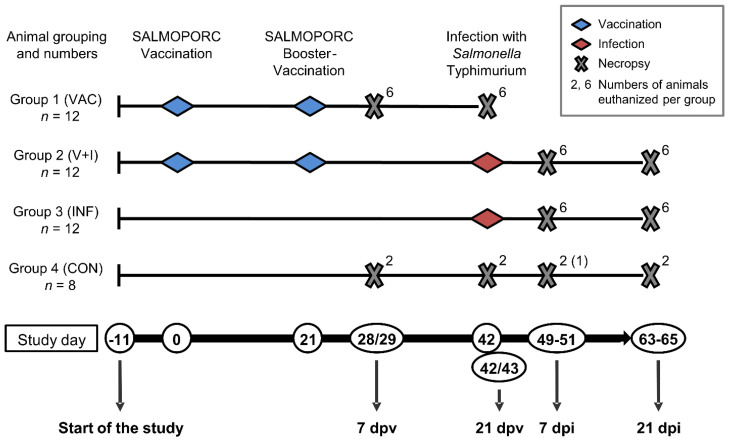
Animal grouping and time schedule for the study. At the beginning of the study (SD-11, study start), 44 piglets at an age of four weeks were divided into four groups: animals that were only vaccinated (group 1, VAC), animals that were vaccinated and challenged (group 2, V+I), animals that only received the challenge infection (group 3, INF) and non-vaccinated non-infected control animals (group 4, CON). First vaccination (SD0), booster vaccination (SD21) and challenge infection (SD42) are indicated by blue and red diamonds, respectively. Animals were euthanized 7 days post vaccination (7dpv, SDs 28/29), 21 days post vaccination (21 dpv, SDs 42/43), 7 days post infection (7 dpi, SDs 49–51) and 21 days post infection (21 dpi, SDs 63–65) as marked by grey crosses. Superscript numbers above crosses indicate numbers of animals euthanized from each group. Due to the loss of animal Sw#3, only one control animal was available for the necropsy date SDs 49–51.

For the duration of the study, all animals were housed in a biosafety level (BSL) 2 facility at the University of Veterinary Medicine Vienna with different treatment groups residing in separate compartments of the isolation unit. Upon arrival at four weeks of age, the animals were weighed and the data were used to achieve equal distribution of animals with different body weights into four groups (Figure 1). Starting at an age of 6 weeks, groups 1 (VAC) and 2 (V+I) were immunized twice orally in a 3-week interval (SD0 and SD21, Figure 1) with 1.0 mL of the live attenuated histidine–adenine auxotrophic STM vaccine (Salmoporc, Ceva Santé Animale, Libourne, France) containing a dose of 1.33 × 10^9^ colony forming units (cfu). Groups 3 (INF) and 4 (CON) received 1.0 mL of tap water orally. Three weeks after the second immunization (SD42), groups 2 (V+I) and 3 (INF) were infected orally with 5 mL per animal containing 2 × 10^8^ cfu/mL of a virulent monophasic STM strain (DT193, no. RKI 06-1900, described by [38] and provided by Ceva Innovation Center GmbH, Dessau-Roßlau, Germany). For the infection, the challenge strain was mixed with sugar beet solution to improve acceptance by the animals. The control group (CON) received 5 mL of sugar beet syrup diluted in water. Both vaccine and challenge strain solutions were applied orally using an oral drencher kit provided by Ceva Santé Animale. Necropsy was performed over two or three consecutive days at 7 and 21 days post vaccination (dpv) and/or infection (dpi): SDs 28/29 were summarized as 7 dpv, SDs 42/43 as 21 dpv, SDs 49–51 as 7 dpi and SDs 63–65 as 21 dpi (Figure 1, bottom). All animals were anaesthetized by intramuscular injection of Ketaminhydrochlorid (Narketan^®^, 10 mg/kg body weight, Vétoquinol, Lure Cedex, France) and Azaperon (Stresnil^®^, 1.3 mg/kg body weight, Elanco, Greenfield, IN, USA) followed by euthanasia via intracardial injection of T61^®^ (tetracaine hydrochloride, mebezonium iodide and embutramide, 1.0 mL/10 kg body weight, MSD Animal Health, Kenilworth, NJ, USA). One animal (Sw#3, Group 4, CON) was unexpectedly found dead on SD7. All results from this animal were excluded from the analyses. The animal experiment was approved by the institutional ethics committee, the Advisory Committee for Animal Experiments (§12 of Law for Animal experiments, Tierversuchsgesetz-TVG) and the Federal Ministry for Science, Research and Economy (BMBWF-68.205/0095-V/3b/2018). 

### 2.3. Clinical Examination, Necropsy, and Sample Collection

Animals were examined daily, and observations were evaluated by a scoring system, taking rectal temperature, diarrhea, vomiting, and changes in behavior into account. Additionally, body weights of all pigs were recorded weekly. After euthanasia, a general pathological examination of the inner organs of all animals was performed. Fecal samples from all animals were collected at the beginning of the study (SDs −11, −10, −9) for STM analysis. Serum samples from the jugular vein (*Vena jugularis*) were taken from all animals upon arrival (SD-11) and from all animals still alive at SD42 prior to challenge infection. At every day of necropsy (SD28/29, 42/43, 49–51, 63–65) blood was drawn by cardiac puncture from the respective animals after anesthesia prior to euthanasia. On these days, samples were collected from liver, spleen, jejunal lymph nodes (JLN), ileocolic lymph nodes (ICLN), jejunum, ileum, and cecum. Tissue samples from liver, jejunum, ileum, and cecum were always taken from the same part of the organ as described in [33].

### 2.4. Detection of Salmonella-Specific Antibodies in Serum

Blood samples were centrifuged for 10 min and 1900× *g* at room temperature and resulting sera were tested for *Salmonella*-specific antibodies by use of a commercially available ELISA kit (IDEXX Swine *Salmonella* Ab test, IDEXX Europe) according to the manufacturer’s instructions. Ratios between mean optical density (OD) of each sample and mean OD of the positive control were calculated and reported as S/P ratios. In addition, serum samples were used to detect *Salmonella*-specific IgM, IgA, and IgG antibodies using an in-house ELISA as described in [15]. Briefly, 96-well plates (Nunc Maxisorp^TM^, Thermo Fisher, Waltham, MA, USA) were coated with 10 µg/mL of the Salmoporc vaccine strain (provided by Ceva Innovation Center GmbH, Dessau-Roßlau, Germany) at 4 °C overnight. Plates were then washed and blocked for 2 h with 1% BSA blocking buffer. Swine sera were diluted 40-fold for IgM, 12-fold for IgA, and 200-fold for IgG detection. These swine sera, after pre-incubation with soluble *E. coli* proteins (provided by Ceva Innovation Center GmbH, Dessau-Roßlau, Germany), were incubated with plate-immobilized STM antigen for 2 h at room temperature. Detection of IgM, IgA, and IgG was achieved with Ig-class-specific secondary goat anti-swine antibodies conjugated to horseradish peroxidase (Bethyl Laboratories, Montgomery, TX, USA) and subsequent incubation with TMB/E (3,3′,5,5′-tetramethylbenzidine) substrate (Sigma-Aldrich, Merck KGaA, Darmstadt, Germany). The reaction was stopped with sulfuric acid and optical densities were measured at a wavelength of 450 nm. An internal reference serum, derived from the combined sera of two V+I animals, euthanized 7 and 21 dpi, respectively, and two INF animals, also euthanized 7 and 21 dpi, served as a positive control on all plates. Results are given as S/P ratio obtained by the ratio between mean OD of individual samples and mean OD of the reference serum.

### 2.5. Microbiological Investigation

Fecal samples taken at the start of the study as well as samples of liver, spleen, JLN, ICLN, jejunum, ileum, and cecum collected at necropsy were examined for the presence of *Salmonella enterica* according to ISO 6579–1:2017 [39]. All samples were streaked onto Xylose-Lysine-Deoxycholate (XLD) agar plates (BBL™, Becton Dickinson (BD), Heidelberg, Germany) in a three-sector T-streak pattern and incubated at 37 °C for 48 h in ambient air. Samples were additionally pre-enriched in buffered peptone water (BPW, Millipore™, Merck KGaA, Darmstadt, Germany) for 24 h at 37 °C. Subsequently, 0.1 mL of each culture was transferred to Rappaport-Vassiliadis R10 and Selenite broth (both Difco™, BD, Heidelberg, Germany). Incubation was performed for 24 h at 42 °C, and sub-cultures streaked onto XLD agar plates (BBL™, BD, Heidelberg, Germany) that were incubated at 37 °C for 48 h under aerobic conditions. MALDI TOF mass spectrometry was employed for identification of putative *Salmonella* colonies. The content of *Salmonella* in a sample was estimated by counting colonies in the three-sector T-streak pattern (direct smear), i.e., if growth was only observed in the first streak, *Salmonella* colony counts were considered rare or few, depending on the number of colonies grown (rare <10 colonies; few >10 colonies); if colonies were observed in the second fraction, the quantity was considered moderate, and if *Salmonella* colonies were noticed in all three streaks, it was considered many. This semi-quantitative analysis was performed for the seven organs mentioned above of all VAC pigs (7 and 21 dpv), for liver, spleen, and JLN of V+I and INF pigs (7 and 21 dpi) as well as all organs from control animals (7 and 21 dpv/dpi).

Samples of ICLN, jejunum, ileum and cecum taken from V+I and INF animals were used to perform quantitative determinations of the challenge strain load (cfu per g tissue) by application of the Koch spread-plate method as described in [12]. In brief, samples from lymph nodes were cut into small pieces and the mucosa of gut samples was gently scraped off. Tissue material was subsequently transferred to centrifuge tubes (Greiner, Kremsmünster, Austria), weighed and frozen at −25 °C until analysis. After thawing, tissue homogenates prepared with an Ultra-Turrax (IKA^®^-Labortechnik, Staufen, Germany) were suspended in physiological saline solution at a 10-fold dilution series and plated on deoxycholate-citrate agar supplemented with ampicillin (10 µg/mL), streptomycine (50 µg/mL), sulfamerazine (100 µg/mL) and tetracycline (10 µg/mL). After incubation at 37 ± 1 °C for 24 h under aerobic conditions, bacterial colonies were counted on petri dishes and the bacterial load was calculated as cfu/g tissue. A verification of the cultures originating from the challenge strain was performed serologically (Enteroclon, sifin diagnostics GmbH, Berlin, Germany). In the cases of no bacterial growth, a qualitative examination was carried out according to DIN ISO 6579–1:2017. Samples being positive only after enrichment were rated with 10 cfu/g in the original tissue. A sample which yielded no *Salmonella* growth after enrichment was rated as 0 cfu/g tissue.

### 2.6. Isolation of Lymphocytes

Peripheral blood mononuclear cells were isolated from heparinized blood by density gradient centrifugation using lymphocyte separation medium (Pancoll human, density: 1.077 g/mL, PAN Biotech, Aidenbach, Germany). Lymphocytes from spleen and mesenteric lymph nodes were collected as previously described [40]. The procedure for the isolation of lamina propria lymphocytes (LPL) from jejunum and ileum was performed as outlined in [33]. A Sysmex XP 300 hematology analyzer (Sysmex Europe GmbH, Norderstedt, Germany) was used to count cell numbers in all tissue preparations.

### 2.7. Preparation of STM Antigen for In Vitro Stimulation

STM antigen used for the in vitro stimulation of lymphocytes were manufactured by culturing the vaccine strain (STM no. 421/125) and the challenge strain (STM no. RKI 06–1900) via two pre-cultures that were prepared in STM 6/83 medium (in-house) at 37 °C and 150 rpm. After a 10 min centrifugation at 7000× *g*, the pellets were re-suspended in PBS. Afterwards, colony forming units were assessed and concentrates were placed for 90 min in a water bath at 60 °C for heat-inactivation. Antigens were then aliquoted and frozen at −80 °C until further use.

### 2.8. In Vitro Stimulation and Intracellular Cytokine Staining (ICS)

For intracellular staining of IFN-γ, TNF-α and IL-17A, round-bottomed 96-well microtiter plates (Greiner Bio One, Frickenhausen, Germany) were seeded with 5 × 10^5^ freshly isolated cells in a final volume of 200 µL/well in cell culture medium (RPMI 1640, PAN Biotech) supplemented with 10% fetal calf serum (FCS, Merck KGaA, Darmstadt, Germany). Heat-inactivated 2.5 × 10^8^ cfu/mL STM vaccine strain or heat-inactivated 2.25 × 10^8^ cfu/mL STM challenge strain were present in the wells for stimulation and plates were cultured for approximately 19 h at 37 °C. Cells incubated in cell culture medium only served as negative controls. Brefeldin A (BD GolgiPlug™, BD Biosciences, San Jose, CA, USA) was added to the microcultures at a final concentration of 1 μg/mL for the final four hours of cultivation. Cultivated cells were harvested and re-suspended in buffer containing PBS with 3% FCS (Merck KGaA). For flow cytometry (FCM)-based phenotyping of lymphocyte subsets, cells were surface-stained with primary monoclonal antibodies directed to CD3 (mIgG1, clone: PPT3, biotinylated, Southern Biotech, Birmingham, AL, USA), CD4 (mIgG2b, clone: 74–12-4, in-house), CD8α (mIgG2a, clone: 76–2-11, in-house) and CD8β-A488 (mIgG1, clone: PPT23, in-house). The secondary staining step included Streptavidin-BV605 (BioLegend, San Diego, CA, USA), goat anti-mouse IgG2b-APC-Cy7 and goat anti-mouse IgG2a-PE-Cy7 (both Southern Biotech, Birmingham, AL, USA). To discriminate dead cells, Fixable Viability Dye eFlour506 (Thermo Fisher, Waltham, MA, USA) was applied according to manufacturer’s protocol at 0.05 μL reactive dye per sample. Free binding sites of secondary antibodies were blocked with whole mouse IgG molecules (2 µg per sample; Jackson ImmunoResearch Laboratories, West Grove, PA, USA). BD Cytofix/Cytoperm^TM^ Fixation/Permeabilization Kit (BD Biosciences, San Jose, CA, USA) was used following the manufacturer’s instructions. Intracellular staining was performed using IFN-γ-PE (mIgG1, clone: P2G10, BD Biosciences, San Jose, CA, USA), TNF-α-PE/Dazzle 594 (mIgG1, clone: Mab11, BioLegend, San Diego, CA, USA) and IL-17A-eF450 (mIgG1, clone: eBio64DEC17, Thermo Fisher, Waltham, MA, USA). Except for the intracellular staining step that encompassed 30 min, all other incubation steps were carried out in 96-well round-bottom plates for 20 min at 4 °C. After two washing steps, samples were left in 50 µL Perm/Wash^TM^ Buffer (BD Biosciences, San Jose, CA, USA) overnight at 4 °C and analyzed the following morning. FCM analyses were performed on a FACSAria^TM^ (BD Biosciences, San Jose, CA, USA) and data of at least 1 × 10^6^ lymphocytes per sample were recorded for blood, spleen, and lymph nodes. For LPL preparations from jejunum and ileum at least 3 × 10^5^ lymphocytes were recorded. FCM data were analyzed with FlowJo^TM^ Software for Windows (Version 10.5.3; BD Biosciences, San Jose, CA, USA). 

### 2.9. Statistical Analysis

**Figure 2:**
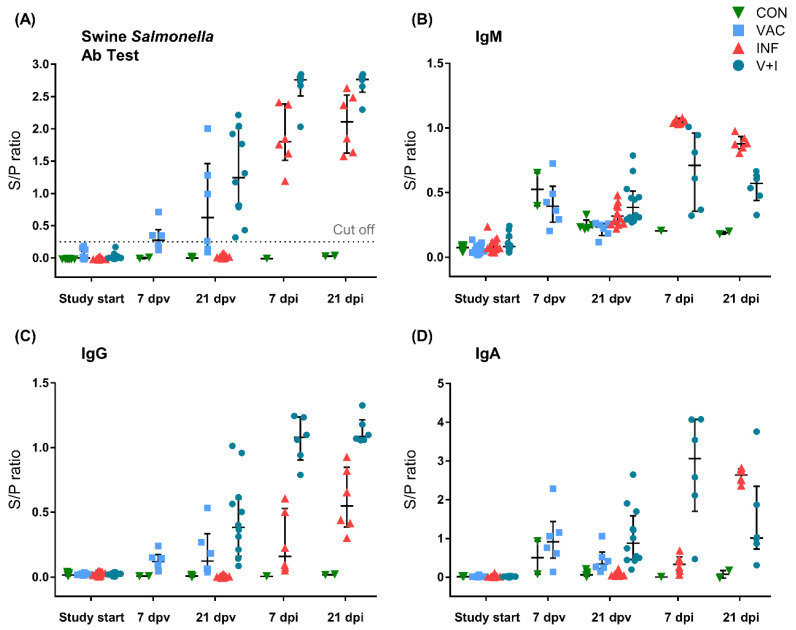
*Salmonella*-specific antibodies in serum. Serum samples were taken on day of arrival (SD-11, study start), 7 days post booster vaccination (7 dpv), on day of challenge infection (21 dpv) as well as 7 and 21 days after infection (7 and 21 dpi, respectively; note: number of serum samples varies on the different time points. Study start: *n* = 43; 7 dpv: *n* = 8; 21 dpv: *n* = 35; 7 dpi: *n* = 13; 21 dpi: *n* = 14). (**A**) *Salmonella*-specific antibodies were measured in the serum using the Swine *Salmonella* Ab Test (IDEXX). Sample to positive control (S/P) ratios were calculated by dividing optical densities (OD) of samples by OD of the positive reference control. The dotted line represents the recommended cut-off for piglets at an S/P ratio of 0.25. Results <0.25 were defined as negative, samples ≥0.25 as positive. (**B**–**D**) *Salmonella*-specific IgM (**B**), IgG (**C**) and IgA (**D**) were measured in the serum by Ig-isotype specific in-house ELISA. S/P ratios were obtained by calculating the ratio between mean OD of individual samples and mean OD of the reference serum. (**A**–**D**) Different colors and symbols represent different groups: green triangles depict control animals, light blue rectangles VAC animals, red triangles INF animals and petrol blue circles show V+I animals. The median is indicated by a central black line with whiskers showing the interquartile range.

**Figure 3:**
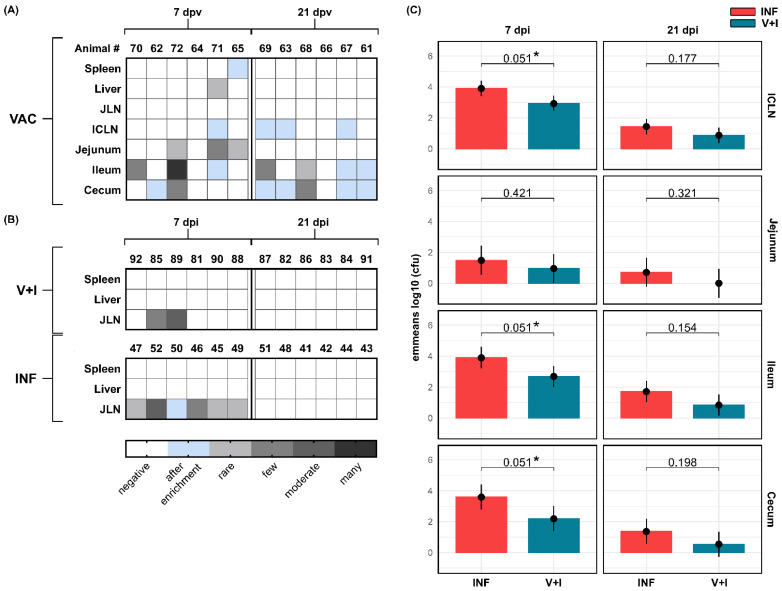
Detection of *Salmonella* Typhimurium in different treatment groups 7 and 21 days post vaccination (dpv)/infection (dpi). (**A**,**B**) Semi-quantitative microbiological analysis was performed for spleen, liver, JLN (all time points), ICLN, jejunum, ileum, and cecum (7/21 dpv only). Results are displayed as heat maps with a grayscale gradient depicting detection of *Salmonella* Typhimurium by agar isolation. Light blue boxes indicate isolation after enrichment. Negative findings for *Salmonella* Typhimurium are displayed in white. (**C**) Quantitative microbiological analysis was performed for ICLN, jejunum, ileum, and cecum from V+I (petrol) and INF animals (red) 7 and 21 dpi. Results are shown as bar charts. Y-axes depict estimated marginal means (emmeans) of the bacterial load (cfu/g tissue) on a log_10_ scale. Numbers above brackets show corresponding multiple testing corrected *p*-values applying a false discovery rate approach across all pairwise comparisons of contrasts between emmeans of V+I and INF animals for each day and tissue (* indicates *p* < 0.1). Black whiskers indicate the lower and upper 95% confidence intervals of emmeans.

**Figure 4:**
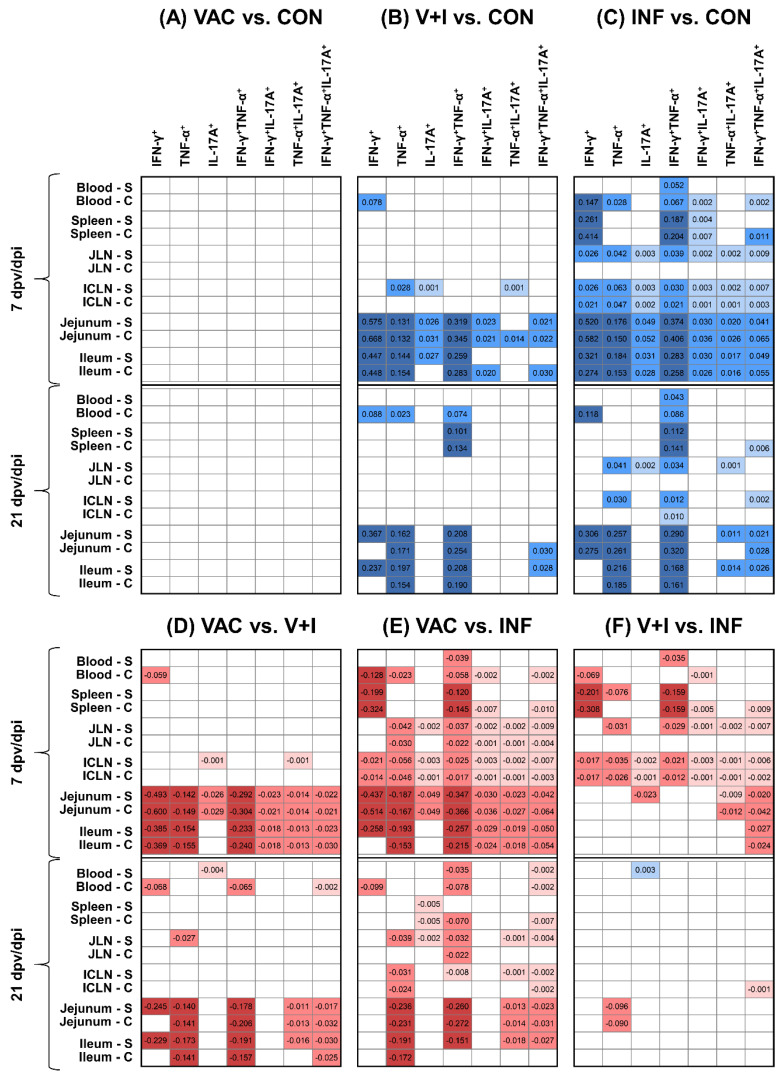
Contrasts of STM-stimulated cytokine-producing CD4^+^ T cells between treatment groups. Contrasts for (**A**) VAC versus CON, (**B**) V+I versus CON, (**C**) INF versus CON, (**D**) VAC versus V+I, (**E**) VAC versus INF, (**F**) V+I versus INF at 7 and 21 days post vaccination (dpv) or infection (dpi) in various tissues. Results are shown as effect size heatmaps. Samples were stimulated in vitro with STM antigen (S: Salmoporc, vaccine strain; C: challenge/infection strain). White boxes represent non-significant differences between groups. Boxes with significant differences are color-coded and contain numbers with corresponding effect sizes also known as contrasts between estimated marginal means on a log_10_ level (dark blue: >0.1, blue: 0.1 to 0.01, light blue: <0.01; dark red: <−0.1, red: −0.1 to −0.01, light red: >−0.01). Boxes in blue indicate STM-stimulated cytokine-producing CD4^+^ T cells are more abundant in the group that is stated first in the comparison. Red boxes indicate higher abundances in the second group. Differences were considered significant at a multiple testing corrected 10% false discovery rate cut-off across all pairwise comparisons (7 and 21 dpv/dpi, four treatment groups, six tissues, and seven phenotypes).

Data for the graphs in Figure 2 and Figures S5–S7 including calculation of median and interquartile range were prepared with GraphPad Prism 7 (GraphPad Software, San Diego, CA, USA). Heatmaps in Figure 3A,B and Figure 4, Figures S9 and S10 were visualized using GraphPad Prism 7. Statistical analysis was performed in R version 3.6.2 (R Core Team (2019). R: A language and environment for statistical computing. R Foundation for Statistical Computing, Vienna, Austria. URL https://www.R-project.org/, accessed on 7 August 2020). 

#### 2.9.1. Bacterial Load

Cfu per gram tissue, measured in ICLN, jejunum, ileum, and cecum, were log_10_ transformed after adding a constant of one to each observation and used as univariate response in linear models applying function *lm* in base R fitting treatment and days after infection as fixed categorical main effects and the interaction between them. Both categorical fixed effects have two levels each; treatment: INF and V+I, and days after infection: 7 and 21 dpi, respectively. The interaction between treatment and days after infection is key in these models. We then calculated estimated marginal means (emmeans) for treatment groups for both days and tested for differences between them with package *emmeans* v1.4.7 [41]. We created bar plots to display the results of our hypothesis testing for estimated marginal means using packages *ggplot2* [42] and *ggpubr* [43] in which the fitted model is shown as black dots and the height of each bar, whiskers represent upper and lower 95% confidence intervals. *p*-values are derived from hypothesis testing of contrasts between estimated marginal means for treatment separately for both days after infection and corrected for multiple testing across all tissues using the false discovery rate approach proposed by Benjamini and Hochberg [44]. Significance was declared at a multiple testing corrected 10% false discovery rate.

#### 2.9.2. T-Cell Response

We analyzed the T-cell response fitting univariate linear mixed models applying function *lmer* in R package *lme4* v1.1–21 [45]. Frequencies of cytokine-producing CD4^+^, CD8^+^ and CD4^−^CD8β^−^ T cells measured in blood, spleen, JLN, ICLN, jejunum, and ileum were log_10_ transformed after adding a constant of one to every observation. Hypothesis testing for CD4^+^ and CD4^−^CD8β^−^ T cells was performed for IFN-γ single-producing, TNF-α single-producing, IL-17A single-producing, IFN-γ/TNF-α co-producing, IFN-γ/IL-17A co-producing, TNF-α/IL-17A co-producing and IFN-γ/TNF-α/IL-17A triple-producing cells. Measures of phenotypes involving IL-17A in CD8^+^ T cells were too low, even after log_10_ transformation to be amenable for linear (mixed) models, i.e., violating the required assumptions of variance homogeneity and normal distribution of residuals and therefore excluded from hypothesis testing. To facilitate the comparison of the STM-specific T-cell response between groups after treatment, necropsy days for the different groups were aligned to 7 days post treatment (7 dpv and 7 dpi) and 21 days post treatment (21 dpv and 21 dpi). Control animals euthanized at 7 dpv/dpi and 21 dpv/dpi, respectively, served as reference values for the respective time points. Main fixed categorical effects in our model are animal treatment (four levels: VAC, V+I, INF, CON), in vitro stimulation (three levels: medium, STM vaccine strain, STM challenge strain) and days after vaccination/infection (two levels: 7 and 21 days) and a triple interaction between them, which is considered the key aspect of our model. The model also contained all pairwise lower-level interaction terms (treatment * stimulation, treatment * days after vaccination/infection, stimulation * days after vaccination/infection). We further included a fixed categorical effect of sex with two levels (male and female) as a nuisance factor. A random intercept animal effect was needed to account for the covariance structure in our data (multiple observations per pig). We used maximum likelihood estimation by setting option REML to false. All assumptions for linear mixed models for the analyzed cytokine phenotypes were met. Residuals and random intercepts were normally distributed and residuals homoscedastic. We verified the absence of collinearity via generalized variance inflation factors [46] using function *vif* in package *car* v3.0–8 [47]. We created bar plots to visualize the results of our hypothesis testing as described for the bacterial load. In short, the height of each bar and the black dots represent the respective estimated marginal means for each treatment group, stimulation level and days after vaccination/infection. Lower and upper 95% confidence intervals of estimated marginal means are shown as black whiskers. *p*-value brackets are derived from hypothesis testing of all pairwise treatment contrasts between estimated marginal means, separately for each level of stimulation and days after vaccination/infection, respectively. These are possible to estimate because of the triple interaction fitted in our models. The y-axes are scaled the same within T-cell subsets for the following three groups of tissues: blood and spleen, JLN and ICLN, jejunum and ileum, but separately for each measured phenotype, to ease interpretation of effect sizes. Multiple testing correction was applied across all tissues and phenotypes within cell type. Significance was declared at a multiple testing corrected 10% false discovery rate [44]. The following two samples were found to be extreme outliers in all T-cell subsets and consequently both removed from the raw ICS data and excluded from hypothesis testing: Animal Sw#69, spleen, stimulation with the challenge strain (due to a staining artefact); animal Sw#90, ileum, stimulation with the challenge strain (due to very low numbers of lymphocytes).

## 3. Results

### 3.1. Clinical Signs

Major aim of the study was the comparison of the T-cell immune response in VAC pigs and V+I pigs versus INF pigs. For animal welfare of INF animals, a lower infection dose was chosen compared to the one used in a previous trial where all animals underwent vaccination prior to challenge infection (1 × 10^9^ cfu/animal compared to 5 × 10^9^ cfu/animal, [33]). Consequently, clinical signs were very mild in all groups (data not shown). All groups showed homogeneous weight gain for most of the study (data not shown). A reduction in average daily gain was observed in both infected groups (V+I pigs: from 1019.5 to 628.6 g/d; INF pigs: from 916.7 to 541.7 g/d) one week after infection. Additionally, rectal temperatures increased in infected animals during a ten-day period starting after the challenge infection. Rectal temperatures during this period ranged from 39.0 °C to 40.4 °C (V+I) and 39.0 °C to 40.5 °C (INF), with differences in average group temperature compared to the CON group reaching a maximum of 0.6 °C on SD50 in both infected groups. During the first three weeks of the study, animals from all groups intermittently showed slightly increased diarrhea scores, likely due to change in diet and stress caused by social group formation. After infection, V+I and INF animals showed slightly increased diarrhea scores in comparison to CON animals, but the levels were similar to what was observed in the adaption period and never exceeded an average group score of 1 (1 = pasty feces).

### 3.2. STM-Specific Antibodies

For the evaluation of the humoral response against STM, both the commercially available IDEXX Swine *Salmonella* Ab test (Figure 2A) and an in-house ELISA test based on whole-cell lysate of purified STM (Figure 2B,C) were used. Negative test results from the IDEXX ELISA verified the *Salmonella*-free status of all animals upon arrival (study start, Figure 2A). All pig sera from the control group remained below the cut-off value of 0.25 throughout the course of the study. S/P ratios of vaccinated animals (VAC and V+I) rose moderately after the second vaccination. A substantial increase was observed in S/P ratios of V+I and INF animals after STM infection with S/P ratios of V+I animals rising above those of INF animals. Although the IDEXX Swine *Salmonella* Ab test is directed against *Salmonella*-specific IgG, the in-house STM ELISA made it possible to distinguish between *Salmonella*-specific IgM, IgA, and IgG. No distinct group-specific impact of the vaccination on S/P ratios of STM-specific IgM was detected (Figure 2B). After infection, however, S/P ratios rose in all infected animals with the highest levels reached by INF pigs. The antibody profile of STM-specific IgG largely matched the results of the IDEXX Swine *Salmonella* Ab test (Figure 2C). Regarding STM-specific IgA, a slight rise of S/P ratios could be observed after vaccination followed by a strong increase in V+I pigs after challenge infection (Figure 2D). S/P ratios of IgA in INF animals experienced a delayed rise but eventually surpassed those of V+I animals 3 weeks post infection. Overall, largest differences between the V+I and INF group were detected at 7 dpi where STM-specific IgG and IgA levels were highest in the V+I group while IgM dominated in INF pigs.

### 3.3. Bacterial Load

Semi-quantitative analysis of the bacterial load did not detect *Salmonella* in any of the collected fecal samples before immunization (SDs −11, −10, −9) nor from any of the organs of the control group at any given time (data not shown). Similarly, spleen and liver were negative for all groups and time points except for two VAC animals from which the vaccine strain could be isolated at 7 dpv (Figure 3A). Although the STM vaccine strain was also found sporadically in ICLN and jejunum, it was more abundant in samples from ileum and cecum, especially 21 dpv. At 7 dpi, *Salmonella* was detected in the JLN of all INF animals (Figure 3B) but not in spleens or livers. In contrast, only in two of the V+I animals could *Salmonella* be isolated from the JLN at this time point. By day 21 post infection, JLN of both groups were negative for *Salmonella*. 

For a more precise evaluation of STM loads in the organs most affected by STM infection, a quantitative detection of the STM challenge strain load was performed for samples of ICLN, jejunum, ileum, and cecum of V+I and INF animals 7 and 21 dpi (Figure 3C). Challenge strain numbers in ICLN as well as ileal and cecal mucosa were significantly reduced (*p* < 0.1) in V+I pigs 7 dpi compared to INF animals. The challenge strain content in the jejunal mucosa was likewise reduced in V+I animals albeit without reaching statistical significance due to large confidence intervals in both groups. The same trend could be observed at 21 dpi for all analyzed organs though without statistically significant differences between the V+I and INF group. Taken together, a lower challenge strain load was found in V+I pigs versus INF pigs for all investigated organs with differences reaching significance in ICLN, ileal, and cecal mucosa at 7 dpi.

### 3.4. STM-Specific Production of IFN-γ, TNF-α and/or IL-17A by CD4^+^, CD8^+^ and CD4^−^CD8β^−^ T Cells

To gain a thorough understanding of the STM-specific T-cell response in the different animal groups at the two time points after treatment, lymphocytes isolated from blood, spleen, JLN, ICLN, jejunum, and ileum at necropsy were stimulated in vitro with either the STM vaccine strain or the STM challenge infection strain and subjected to intracellular cytokine staining. Cells cultivated in medium only acted as negative controls. For the identification of STM-specific cytokine production in T cells, live lymphocytes were gated on CD3^+/dim^CD4^+^ cells to identify CD4^+^ T cells (Figure S1). Within CD3^+^CD4^−^ cells, CD8^+^ T cells were identified by a gate on CD8α^+^CD8β^+^ cells, leaving a remaining subset with a CD4^−^CD8α^+/−^CD8β^−^ phenotype. Subsequently, all three T-cell subsets were further analyzed for IFN-γ, TNF-α and IL-17A production. Representative FCM plots depicting cytokine-producing cells within CD4^+^, CD8^+^ and CD4^−^CD8β^−^ T cells for all treatment groups are shown in Figures S2–S4. For CD4^+^ and CD4^−^CD8β^−^ T cells, seven possible cytokine-producing phenotypes were identified by Boolean combination gates: IFN-γ single-producing, TNF-α single-producing, IL-17A single-producing, IFN-γ/TNF-α co-producing, IFN-γ/IL-17A co-producing, TNF-α/IL-17A co-producing and IFN-γ/TNF-α/IL-17A triple-producing cells. Unsurprisingly, barely any IL-17A production was discovered within CD8^+^ T cells (Figure S3). Consequently, only IFN-γ single-producing, TNF-α single-producing and IFN-γ/TNF-α co-producing CD8^+^ T cells were further investigated.

Frequencies of cytokine-producing T cells for all analyzed phenotypes, stimulation variants, organs, treatment groups and T-cell subsets are summarized as individual graphs in Figure S5 (CD4^+^ T cells), Figure S6 (CD8^+^ T cells) and Figure S7 (CD4^−^CD8β^−^ T cells). The y-axes of these graphs are scaled individually for each cytokine-producing T-cell phenotype but kept the same for the following organ groups: blood and spleen, JLN and ICLN, jejunum and ileum. Looking at all analyzed T-cell subsets on this global level, highest frequencies for most cytokine-related phenotypes were produced by CD4^+^ T cells. In accordance with previously published data [33] and regardless of the cytokine phenotype, frequencies of STM-specific CD4^+^ T cells within the two intestinal sections, jejunum and ileum, largely surpassed those measured in the other organs. 

### 3.5. Treatment-Related Contrasts for STM-Specific Cytokine-Producing CD4^+^ T Cells

Main aim of the study was the comparison of frequencies of cytokine-producing T cells between the four treatment groups to detect differences between the effect of the vaccine and the infection on the cellular immune response of the pigs. To this end, proportions of cytokine-producing T cells for all phenotypes, organs, time points, and treatment groups were modeled in a generalized mixed model. STM-specific T-cell responses were compared between 7 dpv (VAC group) and 7 dpi (V+I and INF group), as well as 21 dpv and 21 dpi. Control animals served as reference values for either 7 dpv/dpi or 21 dpv/dpi, depending on their respective days of euthanasia. 

Since the raw data indicated a dominant role for cytokine-producing CD4^+^ T cells in the response against STM, statistical analysis was first conducted on this T-cell subset. Contrasts between groups that yielded significant *p*-values are highlighted in Figure 4 with their respective effect sizes. Darker colors indicate higher effect sizes. Stimulation during in vitro cultivation with the vaccine strain (Salmoporc, S) and with the challenge infection strain (Challenge, C) yielded largely similar results. By applying our generalized mixed model, no significant differences were detected between VAC and CON animals (Figure 4A). After challenge infection, differences between groups became more pronounced. At 7 dpi, fields with significant contrasts where relative abundances of cytokine-producing CD4^+^ T cells in V+I animals surpassed those of the CON group were largely confined to the intestine (Figure 4B). By contrast, cytokine production by STM-specific CD4^+^ T cells in INF animals at 7 dpi was not only significantly higher in the gut compared to CON animals but also in systemic organs and both lymph nodes (Figure 4C). Two weeks later, at 21 dpi, frequencies for most phenotypes of cytokine-producing CD4^+^ T cells were at lower levels in V+I and INF pigs (Figure S5). Consequently, fewer significant differences could be observed for both groups but still dominated within the contrasts between the INF and the CON group (Figure 4B,C, bottom half). Similar results but with effects into the opposite direction were obtained for comparisons of the VAC group with the V+I and the INF group (Figure 4D,E). 

Overall, highest abundance of significant contrasts and highest effect sizes were obtained for STM-specific IFN-γ single-producing, TNF-α single-producing and IFN-γ/TNF-α co-producing CD4^+^ T cells. Of note, cytokine-producing phenotypes involving IL-17A production such as IFN-γ/IL-17A co-producing, TNF-α/IL-17A co-producing and IFN-γ/TNF-α/IL-17A triple-producing STM-specific CD4^+^ T cells were significantly higher in almost all organs from INF animals at 7 dpi when compared to either the control (Figure 4C) or the VAC group (Figure 4E). Nevertheless, effect sizes stayed below those obtained for IFN-γ single-producing, TNF-α single-producing and IFN-γ/TNF-α co-producing CD4^+^ T cells.

When contrasting the two infected groups, V+I and INF animals, 7 days after infection, higher levels of STM-specific cytokine-producing CD4^+^ T cells were elicited in the INF group with significant differences emerging mainly in blood and spleen as well as the two lymph nodes (Figure 4F), even encompassing all seven cytokine-producing phenotypes in the ICLN. Additionally, IFN-γ/TNF-α/IL-17A triple-producing CD4^+^ T cells were significantly more abundant in jejunum and ileum of INF pigs in comparison to the V+I group. Although significantly higher abundances of cytokine-producing STM-specific CD4^+^ T cells were detected in INF animals compared to V+I pigs at 7 dpi in systemic sites and intestinal lymph nodes, differences in levels of cytokine production between these groups were less pronounced by 21 dpi.

**Figure 5:**
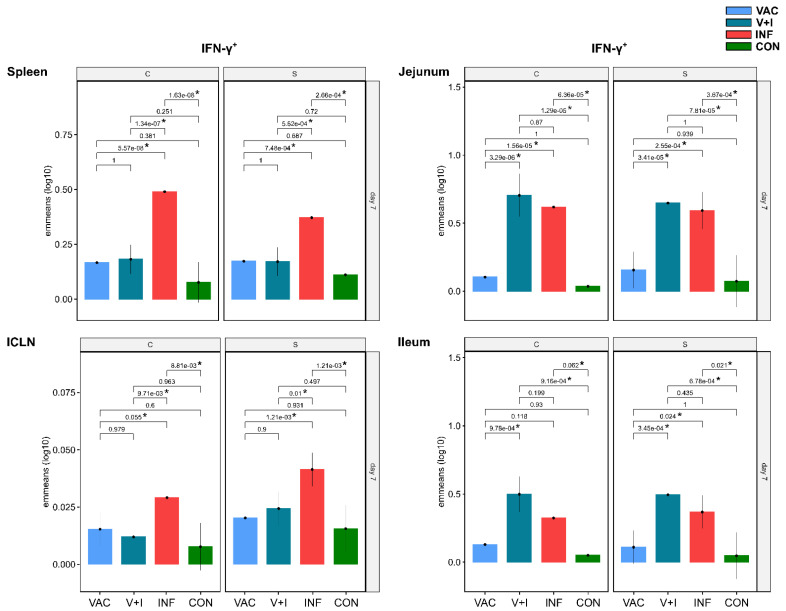
Estimated marginal means (emmeans) of STM-stimulated IFN-γ single-producing CD4^+^ T cells compared across treatment groups 7 days post vaccination/infection in spleen, ICLN, jejunum, and ileum. Samples were stimulated in vitro with STM antigen (S: Salmoporc, vaccine strain; C: challenge/infection strain). Y-axes depict emmeans of STM-stimulated IFN-γ single-producing CD4^+^ T cells for each treatment group on a log_10_ scale. Numbers above brackets show corresponding false discovery rate corrected *p*-values for contrasts between emmeans (* indicates *p* < 0.1) in the VAC (light blue), V+I (petrol), INF (red) and CON group (green). Black whiskers indicate the lower and upper 95% confidence intervals of emmeans. Multiple testing correction was applied across all comparisons (all six pairwise comparisons of the four treatment groups, for 7 and 21 dpv/dpi, for each of the three stimulation levels, six tissues, and seven phenotypes). This leads to a total multiple testing load of 6 ∗ 2 ∗ 3 ∗ 6 ∗ 7 = 1512 comparisons.

**Figure 6:**
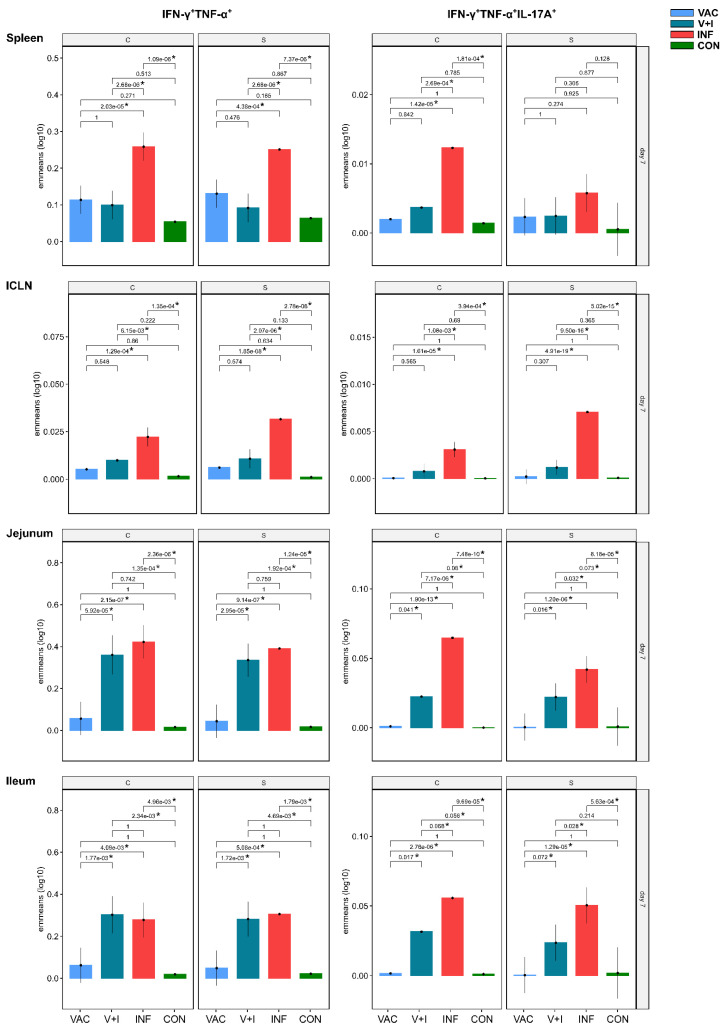
Estimated marginal means (emmeans) of STM-stimulated IFN-γ/TNF-α and IFN-γ/TNF-α/IL-17A co-producing CD4^+^ T cells compared across treatment groups 7 days post vaccination/infection in spleen, ICLN, jejunum, and ileum. Samples were stimulated in vitro with STM antigen (S: Salmoporc, vaccine strain; C: challenge/infection strain). Y-axes depict emmeans of STM-stimulated IFN-γ/TNF-α and IFN-γ/TNF-α/IL-17A co-producing CD4^+^ T cells for each treatment group on a log_10_ scale. Numbers above brackets show corresponding false discovery rate corrected *p*-values for contrasts between emmeans (* indicates *p* < 0.1) in the VAC (light blue), V+I (petrol), INF (red) and CON group (green). Black whiskers indicate the lower and upper 95% confidence intervals of emmeans. Multiple testing correction was applied across all comparisons (all six pairwise comparisons of the four treatment groups, separately for 7 and 21 dpv/dpi, separately for each of the three stimulation levels, six tissues, and seven phenotypes). This leads to a total multiple testing load of 6 ∗ 2 ∗ 3 ∗ 6 ∗7 = 1512 comparisons.

The significant contrasts found between CD4^+^ T cells from V+I and INF animals clearly indicated an effect of the vaccination. This motivated us to scrutinize the CD4^+^ T-cell response after vaccination in the VAC group in more detail. Indeed, a closer look at individual cytokine phenotypes revealed higher emmeans in VAC animals for STM-specific IFN-γ single-producing (Figure 5) and IFN-γ/TNF-α co-producing CD4^+^ T cells (Figure 6) in spleen, ICLN, jejunum, and ileum at 7 dpv when compared to the control group. This was still the case for the two aforementioned phenotypes at 21 dpv in spleen (Figure S8B), ICLN (Figure S8D), jejunum (Figure S8E) and ileum (Figure S8F). Due to the large confidence intervals present in the control group, however, these differences did not reach significance at either time point. Of note, in the spleen, and to some extent in the ICLN, emmeans for IFN-γ single-producing and IFN-γ/TNF-α co-producing CD4^+^ T cells in VAC animals were close to levels obtained in V+I animals. By contrast, and as described for the effect sizes in Figure 4C,E,F, IFN-γ/TNF-α/IL-17A triple-producing CD4^+^ T cells were clearly outstanding in the INF group, followed by the V+I group (Figure 6). 

### 3.6. Treatment-Related Contrasts for STM-Specific Cytokine-Producing CD8^+^ and CD4^−^CD8β^−^ T Cells

In addition to CD4^+^ T cells, proportions of STM-specific cytokine-producing phenotypes were also modeled for CD8^+^ and CD4^−^CD8β^−^ T cells (Figures S9 and S10). In accordance with the raw data (Figures S6 and S7), statistical analysis of both remaining T-cell subsets generated less significant differences between groups than found for CD4^+^ T cells. Regarding CD8^+^ T cells, most instances of significance were found for contrasts of CON or VAC animals with the INF group at 7 dpi (Figure S9). Unlike CD4^+^ T-cell results for the same comparisons, relative abundance of IFN-γ and/or TNF-α producing CD8^+^ T cells were significantly higher in INF animals mostly in the spleen and both lymph nodes, but rarely in the intestine. This could also be observed for STM-specific cytokine-producing CD4^−^CD8β^−^ T cells though analysis of this subset overall provided a more heterogeneous pattern (Figure S10). Similar to CD4^+^ T cells, STM-specific cytokine production by the CD8^+^ and CD4^−^CD8β^−^ subsets was significantly lower in systemic organs and lymph nodes of V+I pigs at 7 dpi when compared to INF pigs. Of all phenotypes analyzed for CD8^+^ and CD4^−^CD8β^−^ T cells, the IFN-γ/TNF-α co-producing phenotype proved to be the most prominent one with higher levels in INF animals compared to all other groups.

## 4. Discussion

STM is a major foodborne pathogen that continues to be highly prevalent in the swine industry. For its control in affected swine herds, vaccination is a recommended and effective measure. Up to date, several inactivated vaccines have shown a reasonable efficacy in controlled as well as in field studies [48,49,50]. Nevertheless, live attenuated commercial vaccines such as Salmoporc (Ceva Santé Animale, Libourne, France) as well as other live vaccine candidates [51,52] are more likely to induce a strong stimulation of the humoral and the cell-mediated immune response. This applies in particular to the Th1 response, which appears to be a key element in controlling STM infections in the mouse model [26,27,53,54] and in pigs [55]. Hence, we considered the use of the live vaccine Salmoporc and its comparison to infection as the most relevant setting to study the porcine T-cell response against STM in detail.

In a previous study, we have shown the induction of multifunctional STM-specific CD4^+^ T cells after vaccinating pigs two times with Salmoporc followed by an infection with STM. To expand on this, we have now investigated the T-cell response in various tissues of only vaccinated (VAC), vaccinated and infected (V+I) as well as only infected (INF) pigs with untreated pigs as a control (CON) group.

To confirm successful vaccination and infection of the animals, serological and bacteriological parameters were ascertained. The vaccine strain could be isolated from the gut tissue of most VAC animals after two immunizations with the Salmoporc vaccine. After challenge infection, V+I animals exhibited significantly lower bacterial loads in ICLN, ileal, and cecal mucosa compared to the INF group. Similar reductions in challenge strain contents of vaccinated animals have been observed in other studies where pigs underwent two oral vaccinations with the Salmoporc vaccine followed by oral challenge infection [12,13,15].

Analysis of serum antibodies with the Swine *Salmonella* Ab Test by IDEXX and an in-house Ig-specific STM ELISA revealed very similar kinetics for STM-specific IgG with both tests. In contrast to S/P ratios of both STM-specific IgG and IgA, no clear vaccination-related elevation of IgM levels could be detected post immunization. Similar observations were made in other studies after oral vaccination of pigs with Salmoporc [15,19]. This finding may be due to natural IgM antibodies which bind with low affinity but are highly cross-reactive and would be present in control and treated animals alike [56]. After challenge infection, S/P ratios of STM-specific IgM increased in all infected animals with highest levels measured in INF animals. This suggests that the more virulent infection strain leads to an activation of naïve B cells that produce bona fide STM-specific IgM antibodies. A considerable elevation was also detected for STM-specific IgG and IgA with S/P ratios of V+I animals far surpassing those of INF animals at 7 dpi. Of note, overall levels of STM-specific IgA were up to 3-fold higher compared to STM-specific IgG. Antigen-specific serum IgG and IgA are mainly produced by long-lived plasma cells in the bone marrow (BM). Interestingly, it has recently been demonstrated that *Salmonella* inhibits IgG-secreting but not IgA-secreting plasma cells in the BM of mice [57]. SiiE, the *Salmonella* protein identified as responsible, seems to compete with laminin on BM stromal cells for binding of IgG-secreting plasma cells leading to their depletion [57]. STM might employ the same strategy in swine to evade humoral immunity, potentially explaining the lower increases in STM-specific serum IgG observed in our and other [15] studies.

To further investigate the T-cell immune response against STM in the pig, we looked for the production of IFN-γ, TNF-α and/or IL-17A by CD4^+^, CD8^+^ and CD4^−^CD8β^−^ T cells in the four different treatment groups. Of all investigated phenotypes, STM-specific IFN-γ/TNF-α co-producing CD4^+^ T cells stood out with the largest number of significant contrasts between groups accompanied by considerable effect sizes, especially in spleen and intestine. Although differences in relative abundances of STM-specific CD4^+^ T cells between VAC and CON animals did not reach significance for any cytokine phenotype, STM-specific IFN-γ/TNF-α co-producing CD4^+^ T cells were induced at low levels in spleen, ICLN, jejunum, and ileum at both 7 and 21 dpv after vaccination which suggests a role in protection. This corresponds to data collected in murine studies, where IFN-γ producing Th1 cells have been manifoldly reported to play a crucial role in controlling STM infection [26,27,53,54]. In fact, best correlates of protection for infections with other intracellular pathogens have been multifunctional CD4^+^ T cells, capable of producing IFN-γ, TNF-α and IL-2 simultaneously [58,59,60]. Regarding the fairly low levels of STM-specific cytokine-producing CD4^+^ T cells in response to vaccination alone, it is conceivable that the two-dose immunization also induced other relevant cell types which were not investigated in this study such as memory B cells or IgA-secreting plasma cells in the intestine. A strong IgA response in particular is desirable for protection of the epithelial barrier against mucosal pathogens and secretory IgA has been shown to have protective capacity against STM in mice [61]. IgA-secreting plasma cells specific for STM LPS have been identified in murine Peyer’s patches after orogastric STM immunization and infection in the streptomycin mouse model of nontyphoidal *Salmonella* [62]; however, this has not yet been done in the pig. This is partially due to a substantial lack of tools to study porcine memory B cells and plasma cells [63] which was also the reason why no further investigations on B cells were performed in this study.

This study also identified IL-17A-producing CD4 T-cell phenotypes. Induction of STM-specific IL-17A single-producing, IFN-γ/IL-17A co-producing, TNF-α/IL-17A co-producing and IFN-γ/TNF-α/IL-17A triple-producing CD4^+^ T cells mainly took place after infection with highest levels reached in INF animals. IL-17A is a key component of mucosal immunity by promoting the recruitment of neutrophils and guarding the integrity of the epithelial barrier [64]. However, IL-17A production is also associated with immunopathology and pathogenic subsets of Th17 cells have been described [65,66]. Our findings suggest that the more virulent infection strain supports the induction of Th17 cells in STM-infected pigs. Due to the chosen low infection dose, which was controlled by the immune system of the INF pigs, these cells may have had a beneficial role in the immune response. However, it is conceivable that at higher doses of infection such cells also contribute to immunopathology. In this context, the restriction of Th17-associated phenotypes to the gut tissue in V+I animals in comparison to INF animals might be interpreted as another beneficial outcome of the vaccination. Indeed, these cells with their inflammatory potential may also under field conditions stay local and therefore contribute to an efficient immune response yet could contribute to a more systemic inflammation in infected animals.

Of key interest in the study was the comparison of the T-cell response between V+I and INF pigs after challenge infection to evaluate possible protective effects of the vaccine. Interestingly, relative abundances of STM-specific cytokine-producing CD4^+^ T cells at 7 dpi were significantly higher in INF animals than in V+I animals for numerous cytokine phenotypes in blood, spleen and JLN, even extending to all seven phenotypes in the ICLN. Significantly higher levels in the gut samples of INF animals were limited to IL-17A containing phenotypes, such as STM-specific TNF-α/IL-17A co-producing and IFN-γ/TNF-α/IL-17A triple-producing CD4^+^ T cells. These findings correspond to microbiological results where bacterial loads in V+I pigs were reduced in the two analyzed lymph nodes and intestinal sections when compared to the INF group. Since significant differences at 7 dpi between the two infected groups mainly centered on systemic organs and lymph nodes, it can be speculated that vaccine-induced STM-specific effector/memory CD4^+^ T cells contribute to a reduction of STM colonization both locally and via homing to the gut thus helping to contain the pathogen in the intestine and hindering systemic dissemination. However, as outlined above, other vaccine-induced immune mechanisms such as local IgA may also have contributed to this reduction in STM colonization. 

Apart from CD4^+^ T cells, we examined other potentially relevant T-cell subsets such as CD8^+^ T cells and CD4^−^CD8β^−^ T cells. Although frequencies of STM-specific cytokine-producing CD8^+^ T cells were lower than those measured for the CD4^+^ or the CD4^−^CD8β^−^ T-cell subset, TNF-α-single and IFN-γ/TNF-α co-producing cells were significantly higher in INF pigs in comparison to the other treatment groups at 7 dpi, mainly in systemic organs and mesenteric lymph nodes. Reports on CD8^+^ T cells in mice have mostly assigned them a modest role in protection against *Salmonella* [67,68]. In contrast, strong CD8^+^ T-cell responses have been observed in humans after oral immunization with a live attenuated *S.* Typhi vaccine and in a challenge infection model with wild type *S*. Typhi [69,70]. However, it must be noted that the experimental layout for T-cell re-stimulation used in our study is not ideal for CD8^+^ T cells. The MHC class I presentation necessary for stimulation of CD8^+^ T cells requires presence of the antigen in the cytoplasm of APCs which is usually not achieved when using inactivated antigen. 

Although relative abundance of cytokine-producing CD4^−^CD8β^−^ T cells also prevailed in INF pigs when compared to the other groups, depiction of significant contrasts yielded a scattered response pattern across phenotypes and organs. This might be explained by the different cell types possibly present in the CD3^+^CD4^−^CD8β^−^ gate such as NKT cells, MAIT cells and γδ T cells. Although a few studies addressed the involvement of γδ T cells, it has been suggested that they contribute to host defense in *Salmonella* infection in mice [71,72], where they have been described as IL-17A-producers in spleen and intestinal mucosa [30,73]. Similarly, in chickens, another γδ-high species such as the pig, increased numbers of CD8α^+high^ γδ T cells were observed in blood, spleen and cecum after STM infection along with enhanced expression levels of IFN-γ mRNA in these cells [74]. Further staining assays that include a marker directed against TCR-γδ are necessary to clarify their role during STM infection in the pig. 

In conclusion, our data suggest an important role of CD4^+^ T cells in protective immune responses against STM in swine with possible additional contributions from B cells, CD8^+^ T cells and γδ T cells. Vaccination of pigs with the live attenuated STM vaccine Salmoporc significantly reduces bacterial colonization in ICLN, ileal and cecal mucosa and stimulates local immunity in the gut thus impeding spread of STM to lymph nodes and systemic organs.

## Data Availability

The data presented in this study are available on request from the corresponding author.

## Supplementary Materials

The following are available online at https://www.mdpi.com/article/10.3390/vaccines9080845/s1, Figure S1: Gating strategy for FCM analyses of CD4^+^, CD8^+^ and CD4^−^CD8β^−^ T cells, Figure S2: Representative raw data for FCM analysis of CD4^+^ T cells, Figure S3: Representative raw data for FCM analysis of CD8^+^ T cells, Figure S4: Representative raw data for FCM analysis of CD4^−^CD8β^−^ T cells, Figure S5: Frequencies of STM-stimulated cytokine-producing CD4^+^ T cells in blood (A), spleen (B), JLN (C), ICLN (D), jejunum (E) and ileum (F), Figure S6: Frequencies of STM-stimulated cytokine-producing CD8^+^ T cells in blood (A), spleen (B), JLN (C), ICLN (D), jejunum (E) and ileum (F), Figure S7: Frequencies of STM-stimulated cytokine-producing CD4^−^CD8β^−^ T cells in blood (A), spleen (B), JLN (C), ICLN (D), jejunum (E) and ileum (F), Figure S8: Estimated marginal means of STM-stimulated IFN-γ/TNF-α/IL-17A producing CD4^+^ T cells in blood compared across treatment groups on day 7 and day 21 post vaccination or infection, Figure S9: Contrasts of STM-stimulated cytokine-producing CD8^+^ T cells between treatment groups at 7 and 21 days post vaccination or infection in various tissues, Figure S10: Contrasts of STM-stimulated cytokine-producing CD4^−^CD8β^−^ T cells between treatment groups at 7 and 21 days post vaccination or infection in various tissues.
