# Supplementary material for: T-Cell Cytokine Response in Salmonella Typhimurium-Vaccinated versus Infected Pigs
Source: Vaccines (Basel). 2021 Aug 2;9(8):845. doi: 10.3390/vaccines9080845 (PMC8402558; doi:10.3390/vaccines9080845)
Supplement: Supplementary file 1 [file vaccines-09-00845-s001.zip › vaccines-1292300-supplementary.pdf]

Supplementary Materials: T-cell cytokine response in *Salmonella* Typhimurium vaccinated versus infected pigs

Selma Schmidt, Heinrich Kreutzmann, Maria Stadler, Kerstin H. Mair, Melissa R. Stas, Michaela Koch, Eleni Vatzia, Sophie Dürlinger, Christian Knecht, Joachim Spergser, Marlies Dolezal, Sven Springer, Tobias Theuß, Vicky Fachinger, Andrea Ladinig, Armin Saalmüller and Wilhelm Gerner*

Figure S1. Gating strategy for FCM analyses of $CD4^{+}$, $CD8^{+}$ and $CD4^{-}CD8\beta^{-}$ T cells. Intracellular cytokine staining was performed on lymphocytes isolated from several tissues following overnight *in vitro* stimulation with STM antigen. The gating strategy for FCM analyses of $CD4^{+}$, $CD8^{+}$ and $CD4^{-}CD8\beta^{-}$ T cells is shown for one representative animal (Sw#89, V+I group, SD50) in one organ (ileum-derived LPL, re-stimulation with the vaccine strain). Cells were gated according to their light scatter properties and further subgated for live cells (VDeFluor506 negative). After exclusion of doublets, a gate was set on $CD3^{+/dim}CD4^{+}$ T cells and remaining cells with a $CD8\beta^{+}$ phenotype were excluded by an additional gate. Subsequently, gates were set on $CD3^{+}CD8\alpha^{+}CD8\beta^{+}$ T cells and $CD3^{+}CD8\alpha^{+/-}CD8\beta^{-}$ T cells. Finally, IFN-γ-, TNF-α- and IL-17A-producing cells were identified for all three T-cell subsets and subjected to a Boolean analysis.

Figure S2. Representative raw data for FCM analysis of $CD4^{+}$ T cells. Intracellular cytokine staining was performed on lymphocytes isolated from several tissues following overnight *in vitro* stimulation with STM antigen. Co-production of IFN-γ/TNF-α, IFN-γ/L-17A and TNF-α/IL-17A in $CD4^{+}$ T cells is shown for blood, spleen, ICLN and ileum for samples that were stimulated with the vaccine strain. Representative data from one pig of each treatment group is shown: CON (Sw#6), VAC (Sw#65), V+I (Sw#89) and INF (Sw#52). Approximately $1x10^{5}$ cells are displayed for blood and spleen, $2x10^{5}$ cells for ICLN and $5x10^{4}$ cells for the ileum.

Figure S3. Representative raw data for FCM analysis of $CD8^{+}$ T cells. Intracellular cytokine staining was performed on lymphocytes isolated from several locations following overnight *in vitro* stimulation with STM antigen. Co-production of IFN-γ/TNF-α, IFN-γ/L-17A and TNF-α/IL-17A in $CD8^{+}$ T cells is shown for blood, spleen, ICLN and ileum for samples that were stimulated with the vaccine strain. Representative data is shown from one pig per treatment group: CON (Sw#6), VAC (Sw#65), V+I (Sw#89) and INF (Sw#52). Approximately $5x10^{4}$ cells are displayed for blood and spleen, $1x10^{5}$ cells for ICLN and ileum.

Figure S4. Representative raw data for FCM analysis of $CD4^{-}CD8\beta^{-}$ T cells. Intracellular cytokine staining was performed on lymphocytes isolated from several locations following overnight *in vitro* stimulation with STM antigen. Co-production of IFN-γ/TNF-α, IFN-γ/L-17A and TNF-α/IL-17A in $CD4^{-}CD8\beta^{-}$ T cells is shown for blood, spleen, ICLN and ileum for samples that were stimulated with the vaccine strain. Representative data is shown from one pig per treatment group: CON (Sw#6), VAC (Sw#65), V+I (Sw#89) and INF (Sw#52). Approximately $1x10^{5}$ cells are displayed for the blood, $3x10^{5}$ cells for the spleen, $3x10^{4}$ cells for ICLN and $6x10^{4}$ cells for the ileum.

Figure S5. Frequencies of STM-stimulated cytokine-producing $CD4^{+}$ T cells in blood (A), spleen (B), JLN (C), ICLN (D), jejunum (E) and ileum (F). $CD4^{+}$ T cells were gated within live lymphocytes and further analyzed for IFN-γ, TNF-α and IL-17A production by Boolean gating. Individual graphs indicate percentages of cytokine-producing $CD4^{+}$ T cells from individual animals of the VAC (light and dark blue), the V+I (light and dark petrol) and the INF (light and dark red) group within total $CD4^{+}$ T cells. Animals were euthanized either 7 days post vaccination/infection (7 dpv/dpi, circles, lighter color) or 21 days post vaccination/infection (21 dpv/dpi, rectangles, darker color). All data obtained from control pigs (CON) is displayed in green triangles on the right

of each graph. Cells were stimulated with the vaccine strain (S, Salmoporc) or the challenge infection strain (C, Challenge) or cultivated in medium-only (M, Medium). Black bars indicate the median and whiskers show the interquartile range. Y-axes are scaled individually per cytokine-producing phenotype but consistent within related organ groups: blood+spleen, JLN+ICLN and jejunum+ileum.

Figure S6. Frequencies of STM-stimulated cytokine-producing $CD8^+$ T cells in blood (A), spleen (B), JLN (C), ICLN (D), jejunum (E) and ileum (F). $CD8^+$ T cells were gated within live lymphocytes and further analyzed for IFN-γ, TNF-α and IL-17A production by Boolean gating. Individual graphs indicate percentages of cytokine-producing $CD8^+$ T cells from individual animals of the VAC (light and dark blue), the V+I (light and dark petrol) and the INF (light and dark red) group within total $CD8^+$ T cells. Animals were euthanized either 7 days post vaccination/infection (7 dpv/dpi, circles, lighter color) or 21 days post vaccination/infection (21 dpv/dpi, rectangles, darker color). All data obtained from control pigs (CON) is displayed in green triangles on the right of each graph. Cells were stimulated with the vaccine strain (S, Salmoporc) or the challenge infection strain (C, Challenge) or cultivated in medium-only (M, Medium). Black bars indicate the median and whiskers show the interquartile range. Y-axes are scaled individually per cytokine-producing phenotype but consistent within related organ groups: blood+spleen, JLN+ICLN and jejunum+ileum.

Figure S7. Frequencies of STM-stimulated cytokine-producing $CD4^-CD8\beta^-$ T cells in blood (A), spleen (B), JLN (C), ICLN (D), jejunum (E) and ileum (F). $CD4^-CD8\beta^-$ T cells were gated within live lymphocytes and further analyzed for IFN-γ, TNF-α and IL-17A production by Boolean gating. Individual graphs indicate percentages of cytokine-producing $CD4^-CD8\beta^-$ T cells from individual animals of the VAC (light and dark blue), the V+I (light and dark petrol) and the INF (light and dark red) group within total $CD4^-CD8\beta^-$ T cells. Animals were euthanized either 7 days post vaccination/infection (7 dpv/dpi, circles, lighter color) or 21 days post vaccination/ infection (21 dpv/dpi, rectangles, darker color). All data obtained from control pigs (CON) is displayed in green triangles on the right of each graph. Cells were stimulated with the vaccine strain (S, Salmoporc) or the challenge infection strain (C, Challenge) or cultivated in medium-only (M, Medium). Black bars indicate the median and whiskers show the interquartile range. Y-axes are scaled individually per cytokine-producing phenotype but consistent within related organ groups: blood+spleen, JLN+ICLN and jejunum+ileum.

Figure S8. Estimated marginal means (emmeans) of STM-stimulated IFN-γ/TNF-α/IL-17A producing $CD4^+$ T cells in blood compared across treatment groups on day 7 and day 21 post vaccination or infection. Samples were stimulated *in vitro* with STM antigen (S: Salmoporc, vaccine strain; C: challenge/infection strain) or cultivated in medium-only (M: Medium). (A) Y-axes depict emmeans of STM-stimulated IFN-γ/TNF-α/IL-17A producing $CD4^+$ T cells for each treatment group on a $\log_{10}$ scale. Numbers above brackets show corresponding *p*-values calculated from contrasts between emmeans in the VAC (light blue), V+I (petrol), INF (red) and CON group (green). Black whiskers indicate the lower and upper 95% confidence intervals of emmeans. Multiple testing correction was applied across all comparisons (all six pairwise comparisons of the four treatment groups, separately for 7 and 21 dpv/dpi, separately for each of the three stimulation levels, six tissues and seven phenotypes). This leads to a total multiple testing load of 6*2*3*6*7=1512 comparisons. (B-F) Same as (A) but for cytokine-producing $CD4^+$ T cells derived from spleen (B), JLN (C), ICLN (D), jejunum (E) and ileum (F).

Figure S9. Contrasts of STM-stimulated cytokine-producing $CD8^+$ T cells between treatment groups at 7 and 21 days post vaccination (dpv) or infection (dpi) in various tissues. Results are shown as effect size heatmaps. Samples were stimulated *in vitro* with STM antigen (S: Salmoporc, vaccine strain; C: challenge/infection strain). White boxes represent non-significant differences between groups. Boxes with significant differences are color-coded and contain numbers with corresponding effect sizes aka contrasts between estimated marginal means on a $\log_{10}$ level (dark blue: > 0.1, blue: 0.1 to 0.01, light blue: < 0.01; dark red: < -0.1, red: -0.1 to -0.01, light red: > -0.01). Boxes in blue indicate STM-stimulated cytokine-producing $CD8^+$ T cells are more abundant in the group

that is stated first in the comparison. Red boxes indicate higher abundances in the second group. Differences were considered significant at a multiple testing corrected 10% false discovery rate cut-off across all pairwise comparisons (7 and 21 dpv/dpi, four treatment groups, six tissues and three phenotypes).

Figure S10. Contrasts of STM-stimulated cytokine-producing $CD4^-CD8\beta^-$ T cells between treatment groups at 7 and 21 days post vaccination (dpv) or infection (dpi) in various tissues. Results are shown as effect size heatmaps. Samples were stimulated *in vitro* with STM antigen (S: Salmoporc, vaccine strain; C: challenge/infection strain). White boxes represent non-significant differences between groups. Boxes with significant differences are color-coded and contain numbers with corresponding effect sizes aka contrasts between estimated marginal means on a $\log_{10}$ level (dark blue: > 0.1, blue: 0.1 to 0.01, light blue: < 0.01; dark red: < -0.1, red: -0.1 to -0.01, light red: > -0.01). Boxes in blue indicate STM-stimulated cytokine-producing $CD4^-CD8\beta^-$ T cells are more abundant in the group that is stated first in the comparison. Red boxes indicate higher abundances in the second group. Differences were considered significant at a multiple testing corrected 10% false discovery rate cut-off across all pairwise comparisons (7 and 21 dpv/dpi, four treatment groups, six tissues and seven phenotypes).

Figure S1

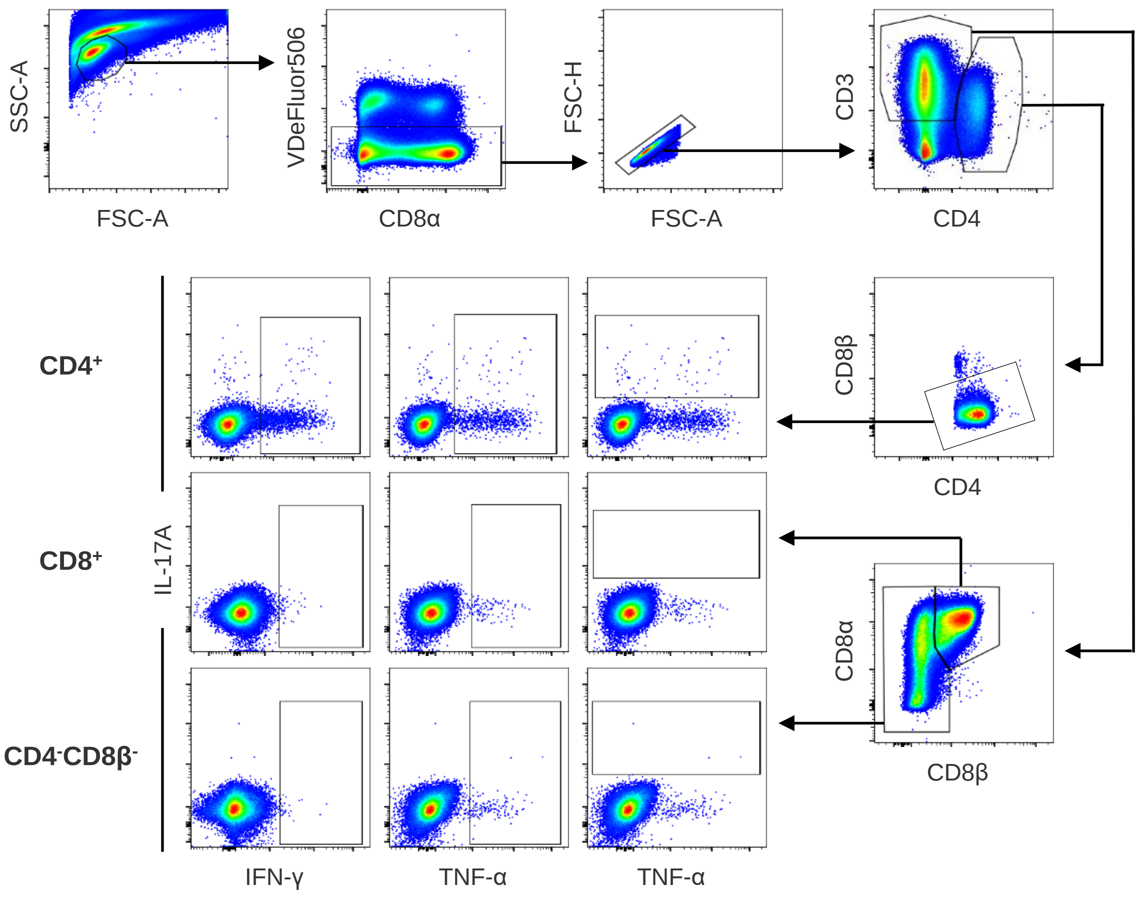

SSC-A
FSC-A
VDeFluor506
CD8α
FSC-H
FSC-A
CD3
CD4
CD4+
CD8β
CD4
CD8+
IL-17A
CD8α
CD8β
CD4-CD8β-
IFN-γ
TNF-α
TNF-α

Figure S2: CD4$^+$ T cells

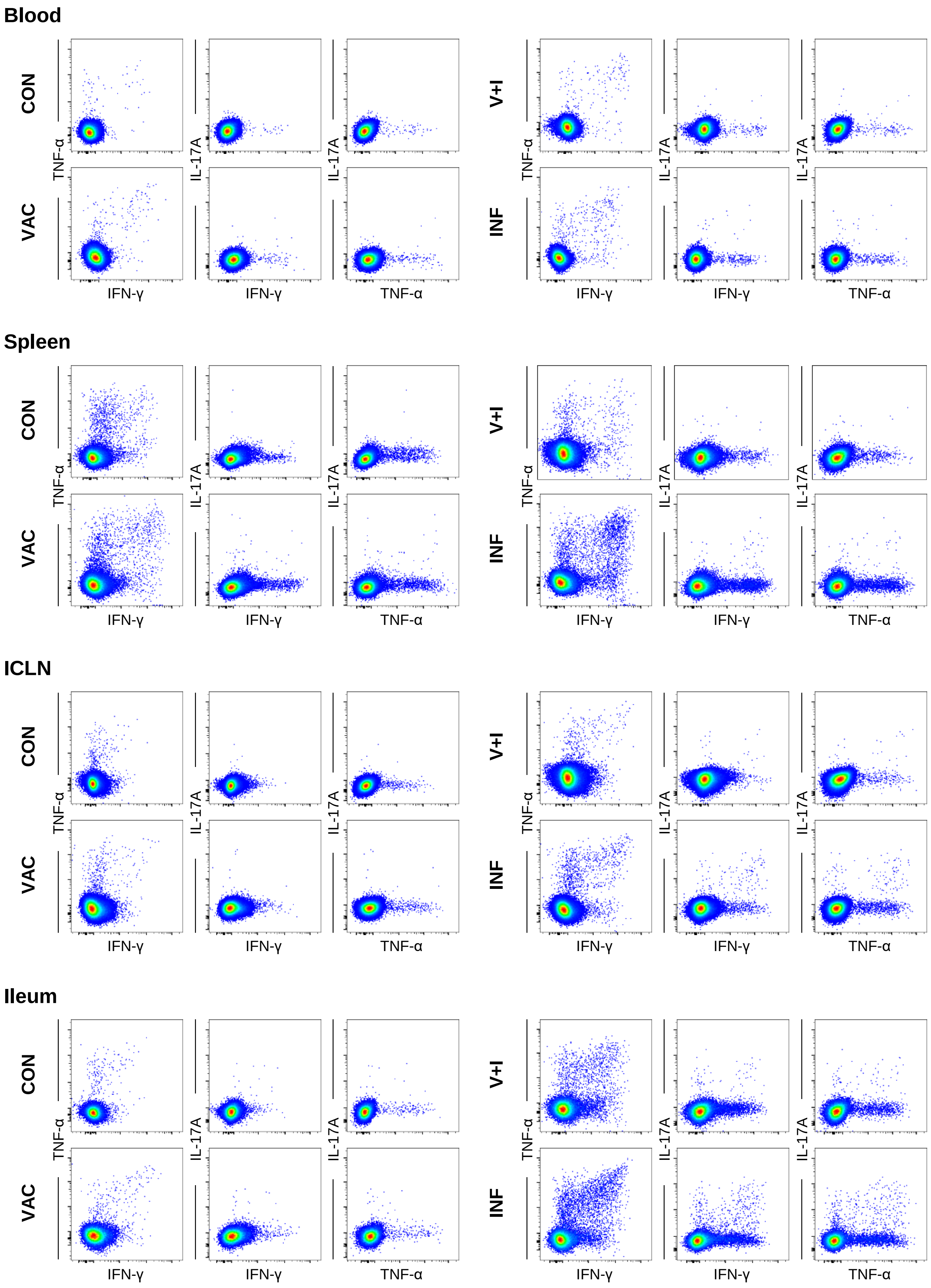

Figure S3: CD8$^+$ T cells

Blood

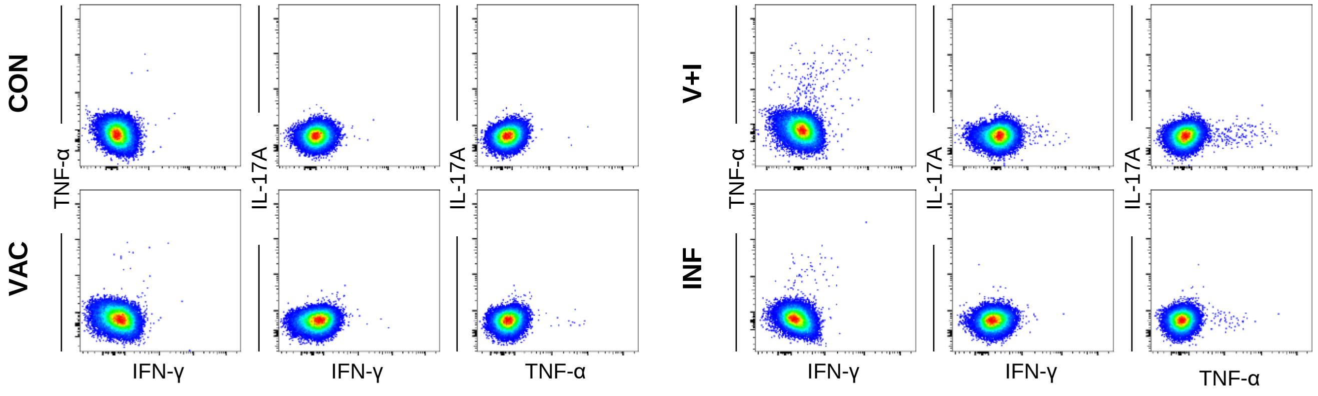

Spleen

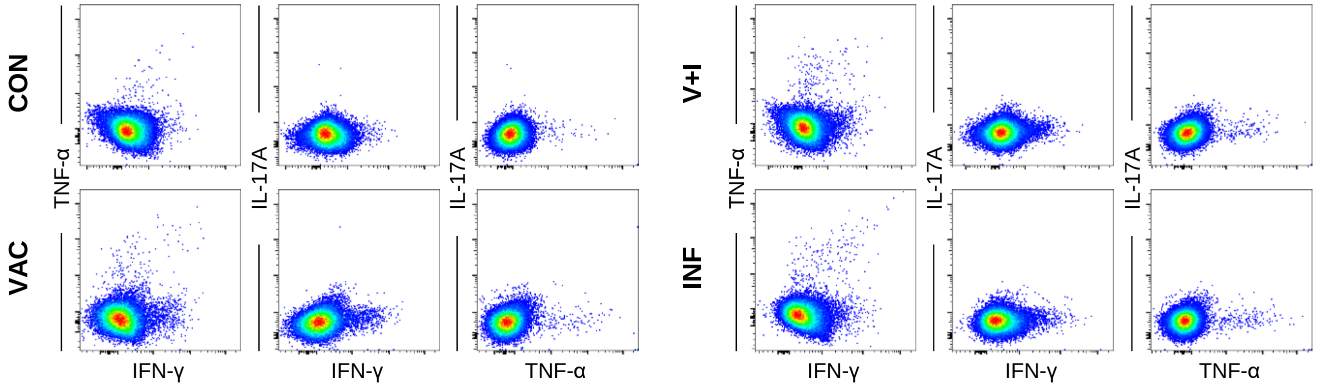

ICLN

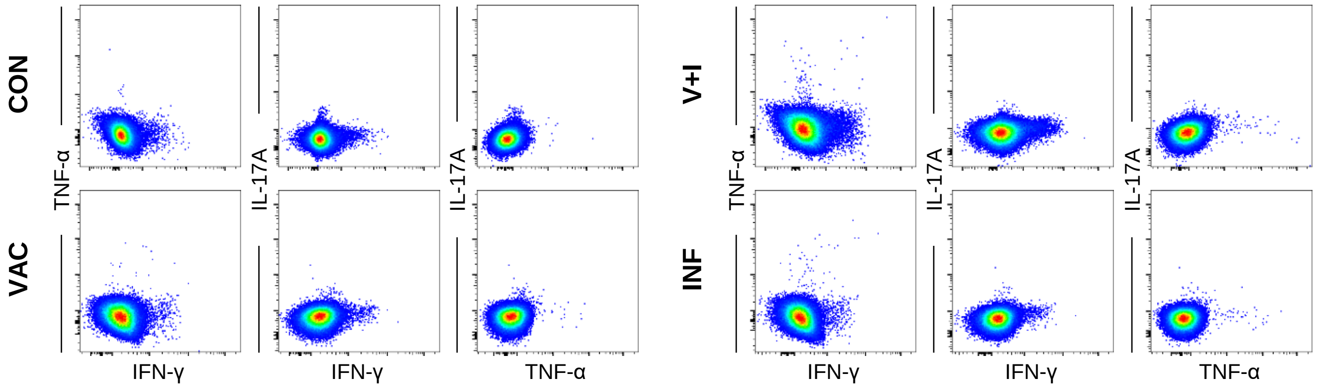

Ileum

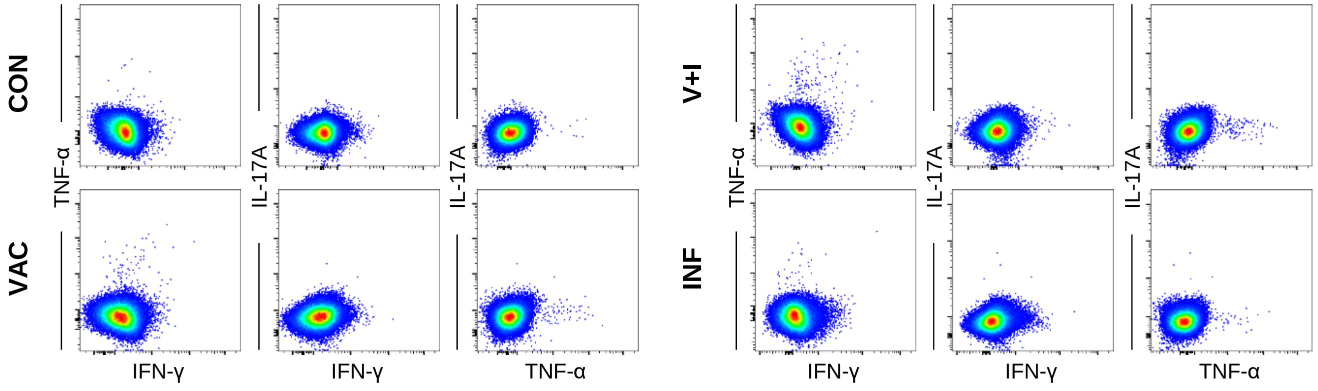

Figure S4: CD4$^-$CD8β$^-$ T cells

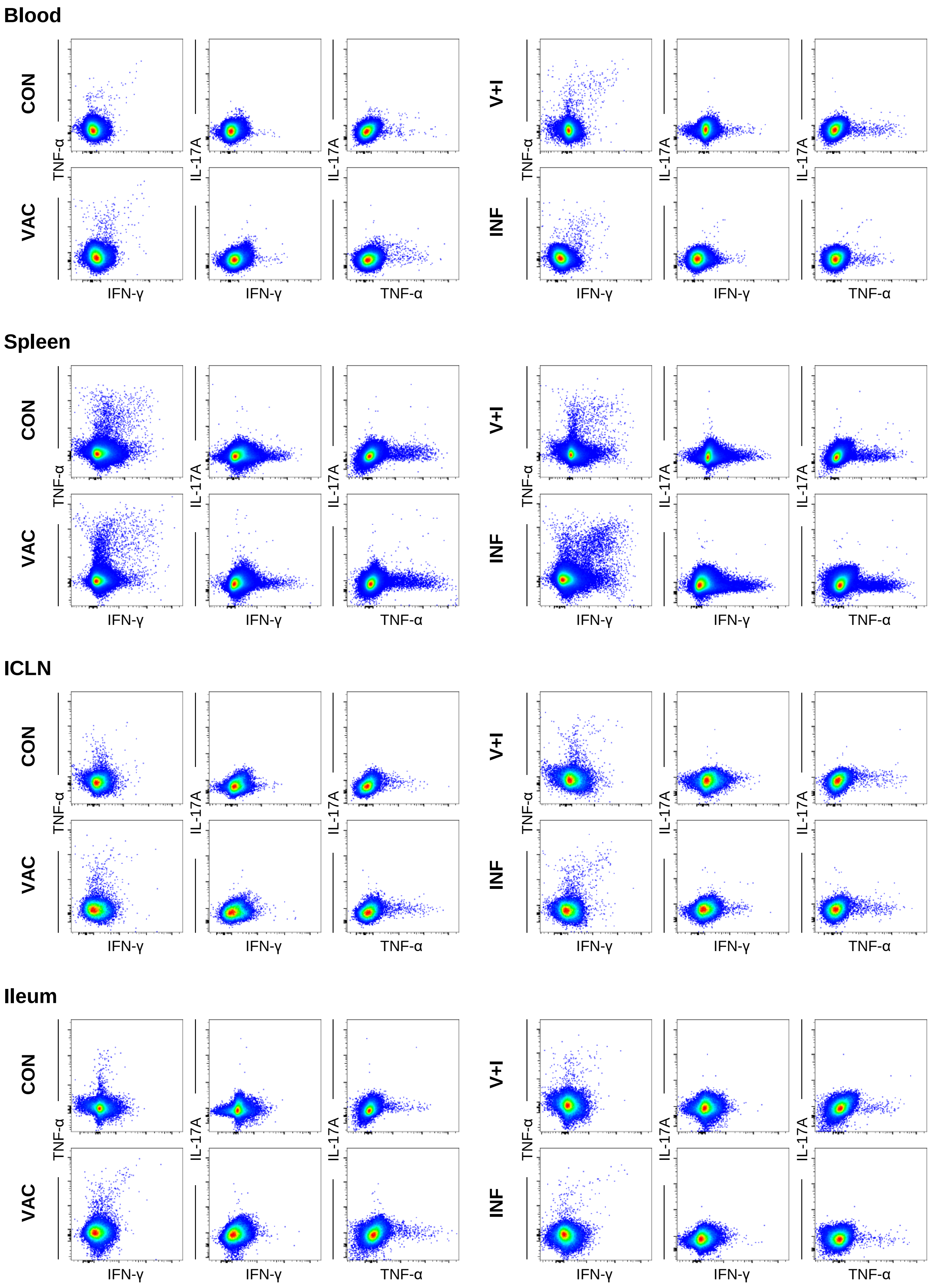

Figure S5A: CD4⁺ T cells; Blood

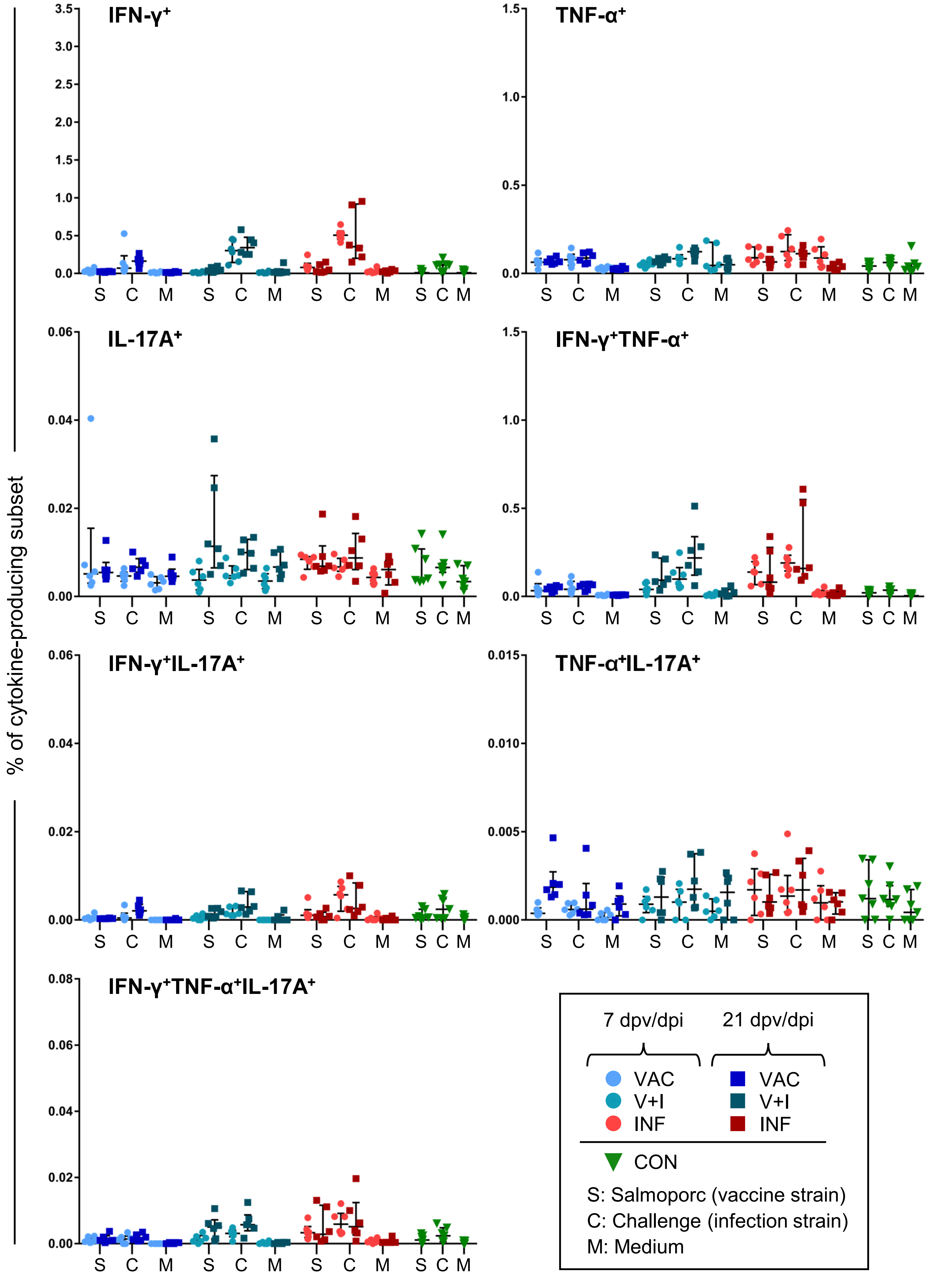

Figure S5B: CD4⁺ T cells; Spleen

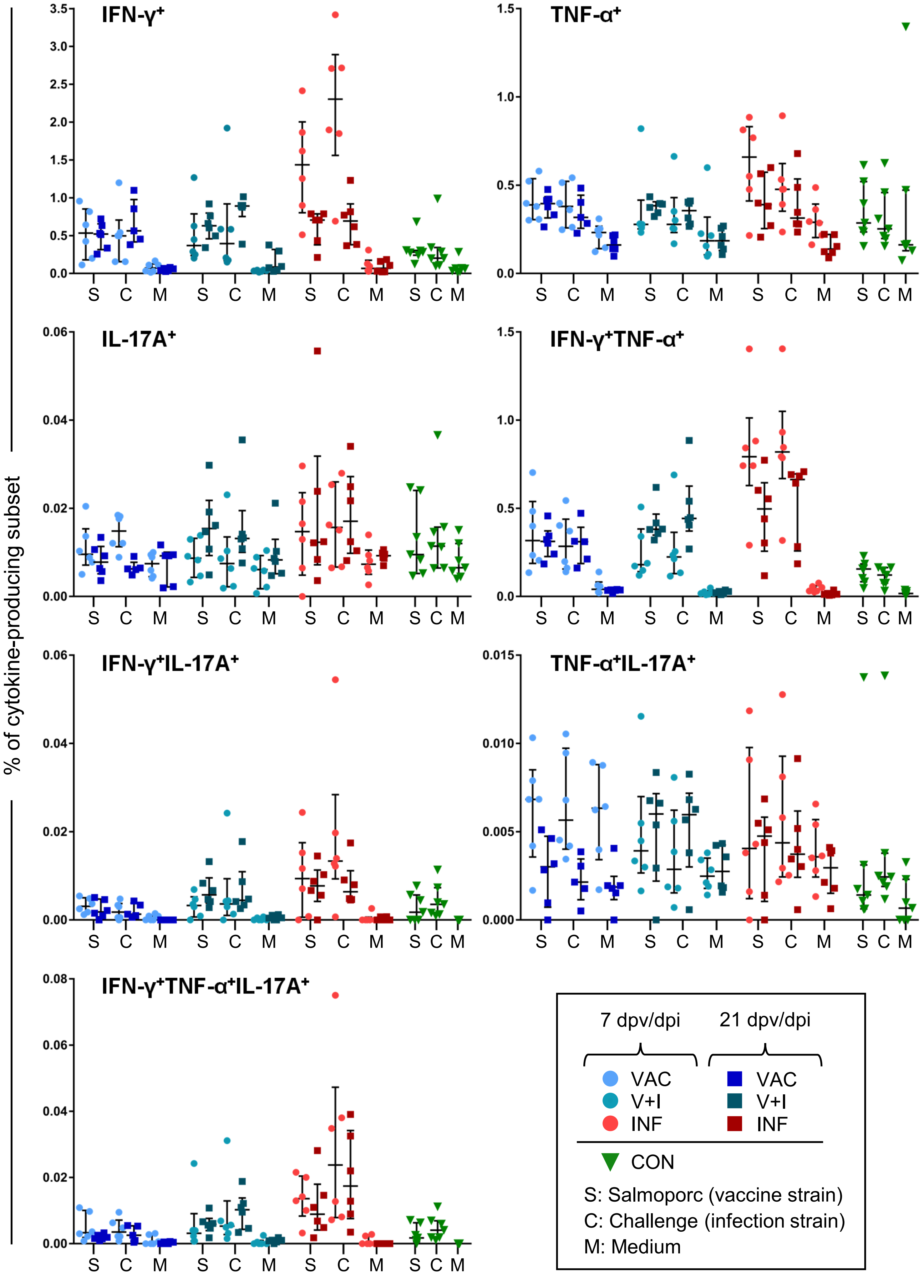

Figure S5C: CD4⁺ T cells; JLN

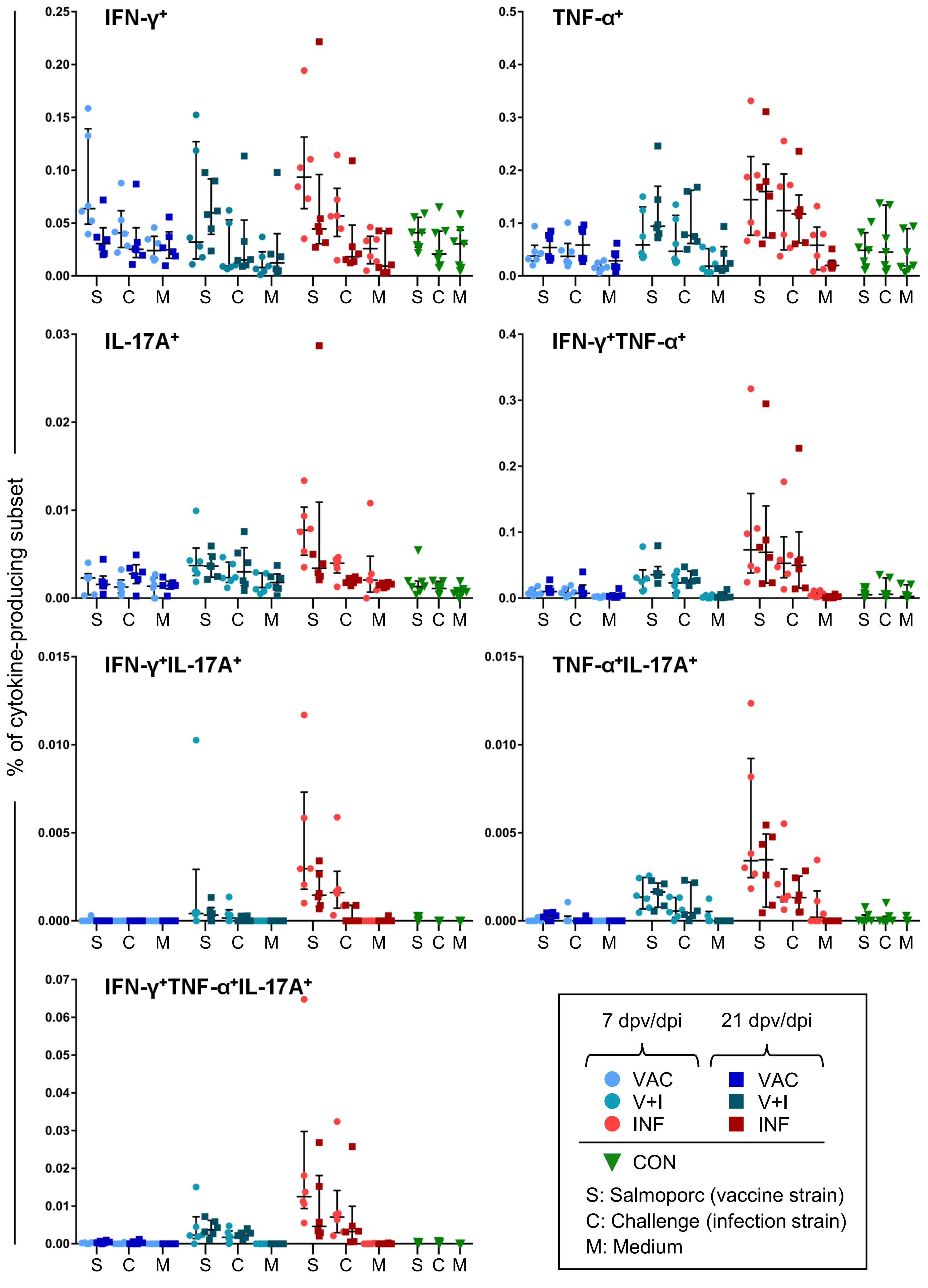

Figure S5D: $CD4^+$ T cells; ICLN

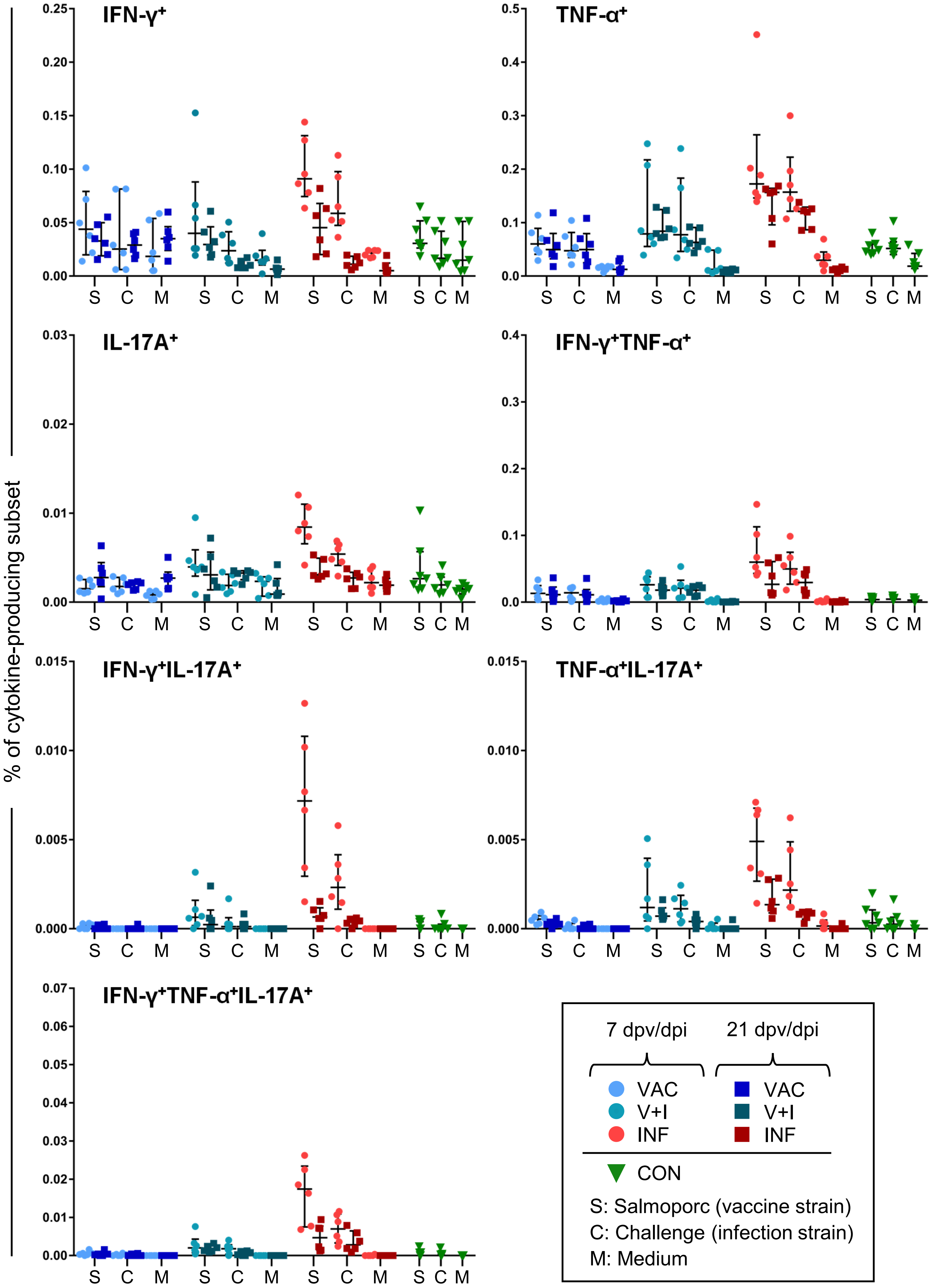

Figure S5E: CD4+ T cells; Jejunum

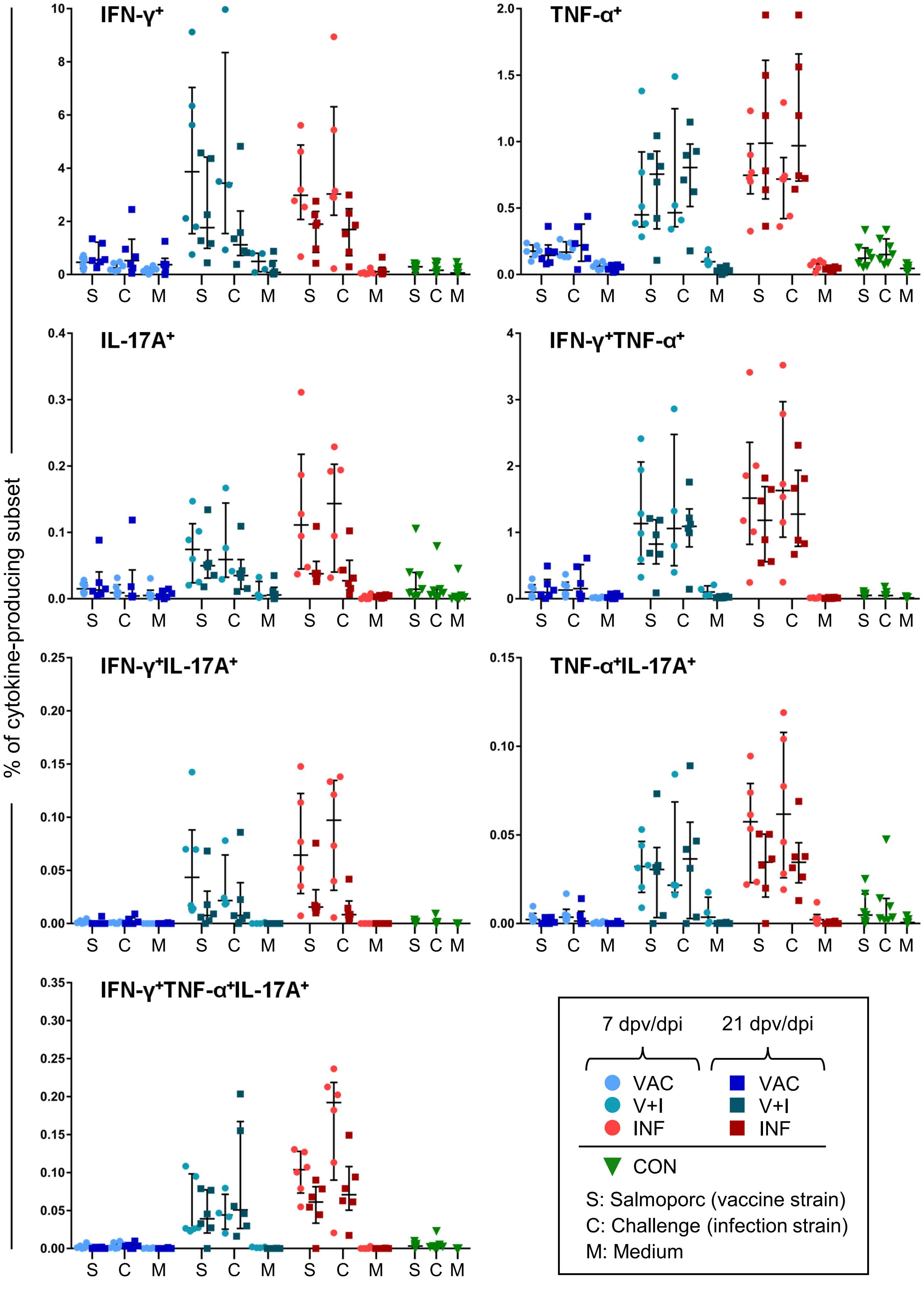

Figure S5F: CD4⁺ T cells; Ileum

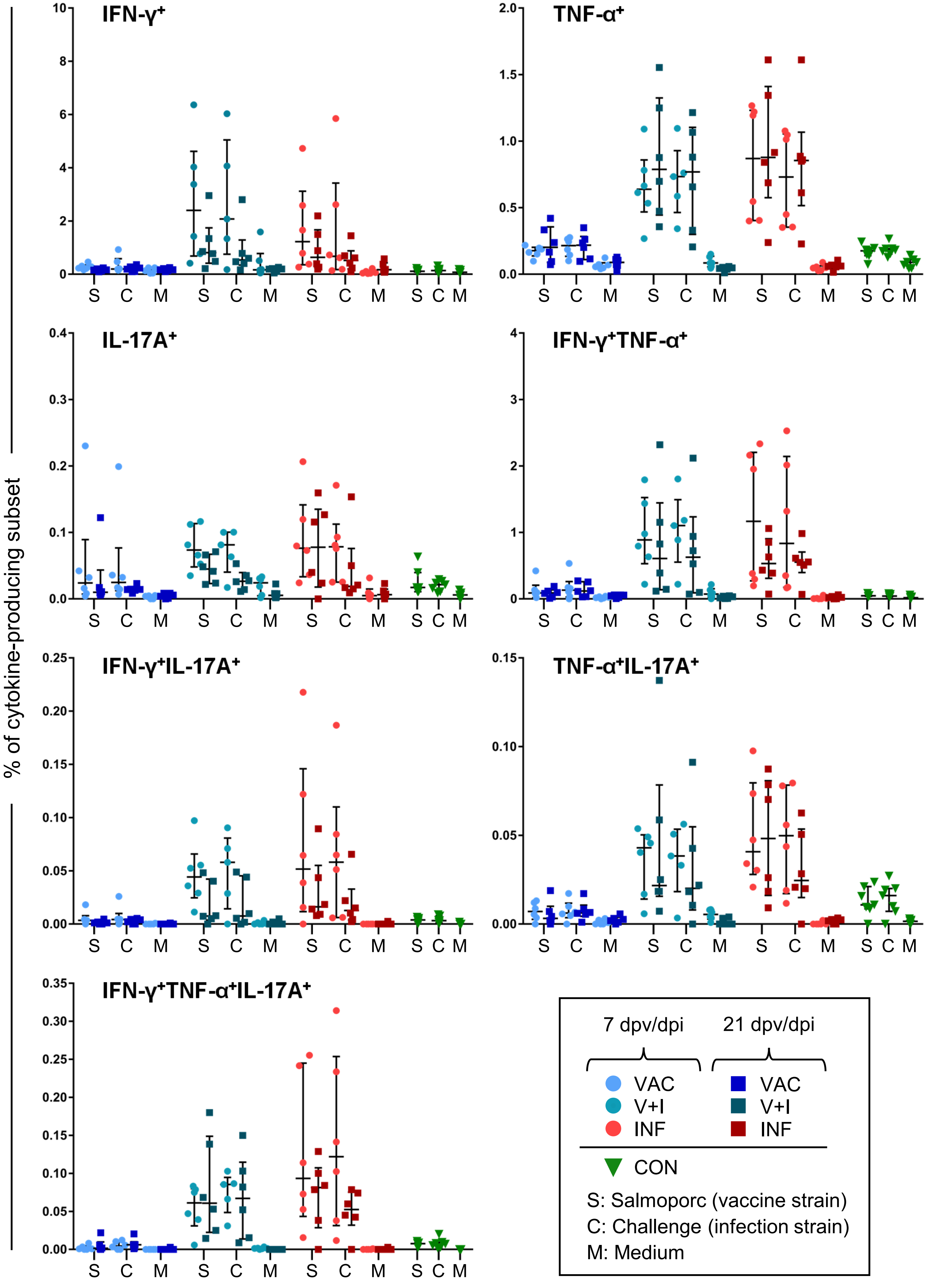

Figure S6A: CD8⁺ T cells; Blood

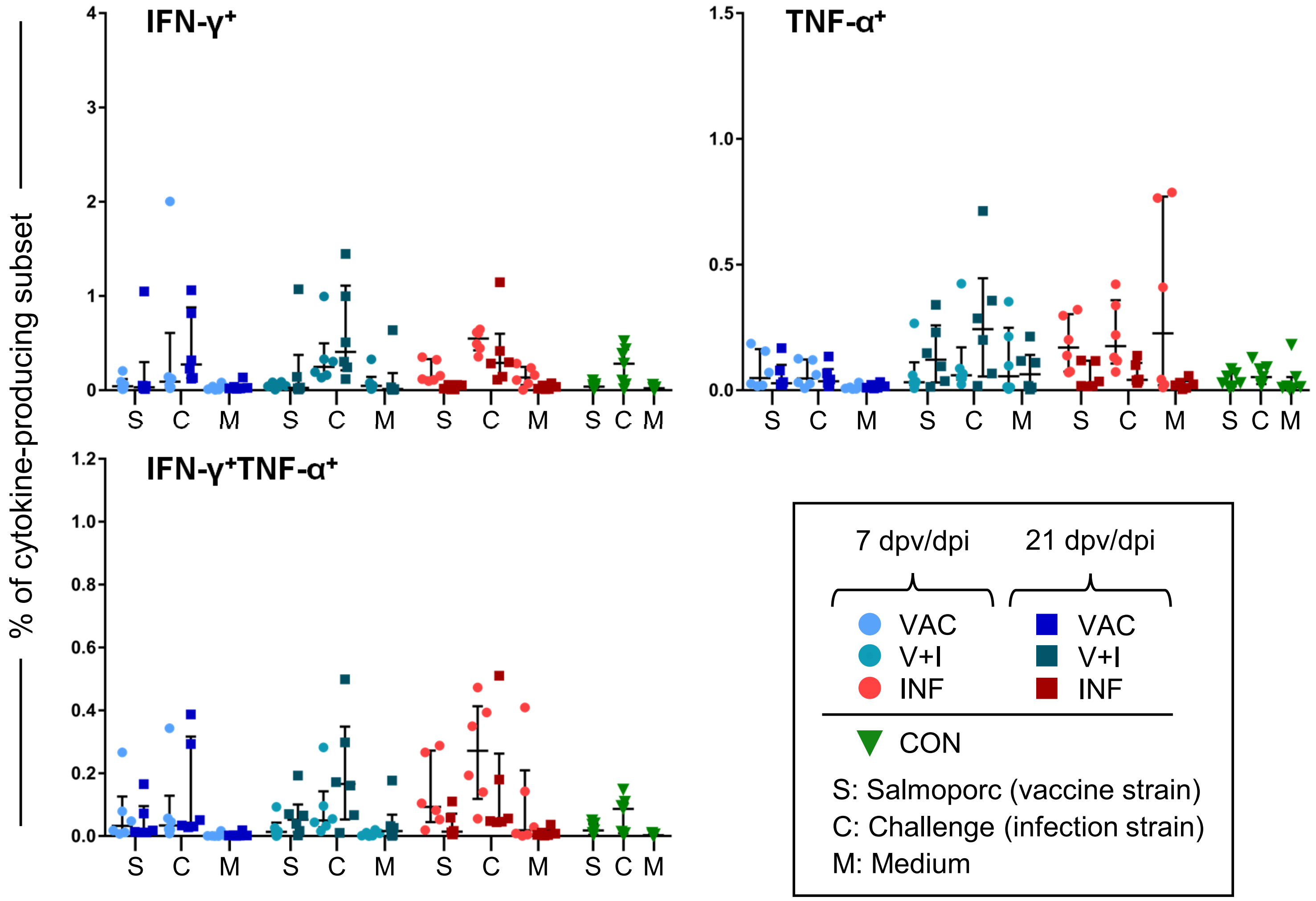

Figure S6B: CD8⁺ T cells; Spleen

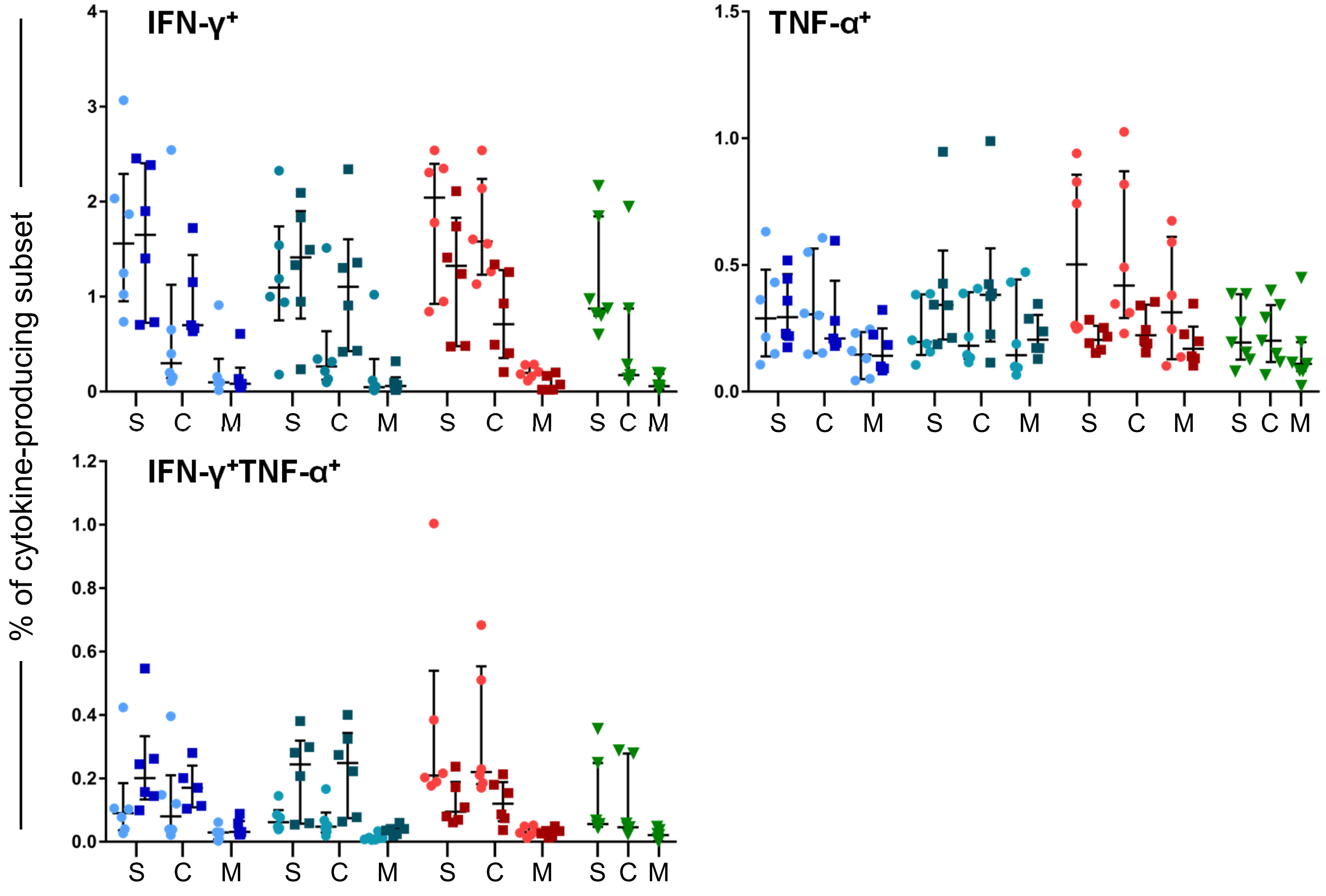

Figure S6C: CD8⁺ T cells; JLN

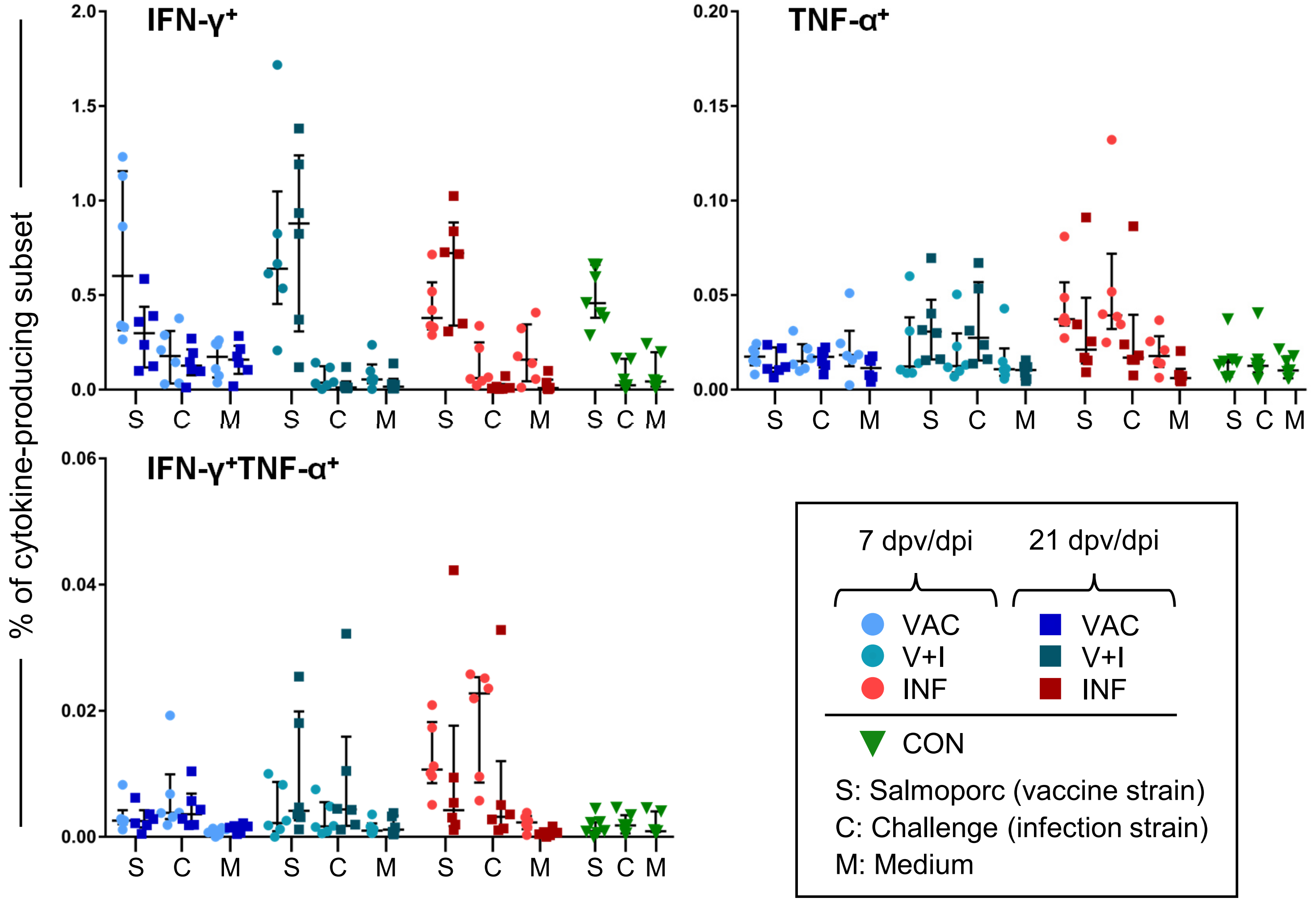

Figure S6D: CD8⁺ T cells; ICLN

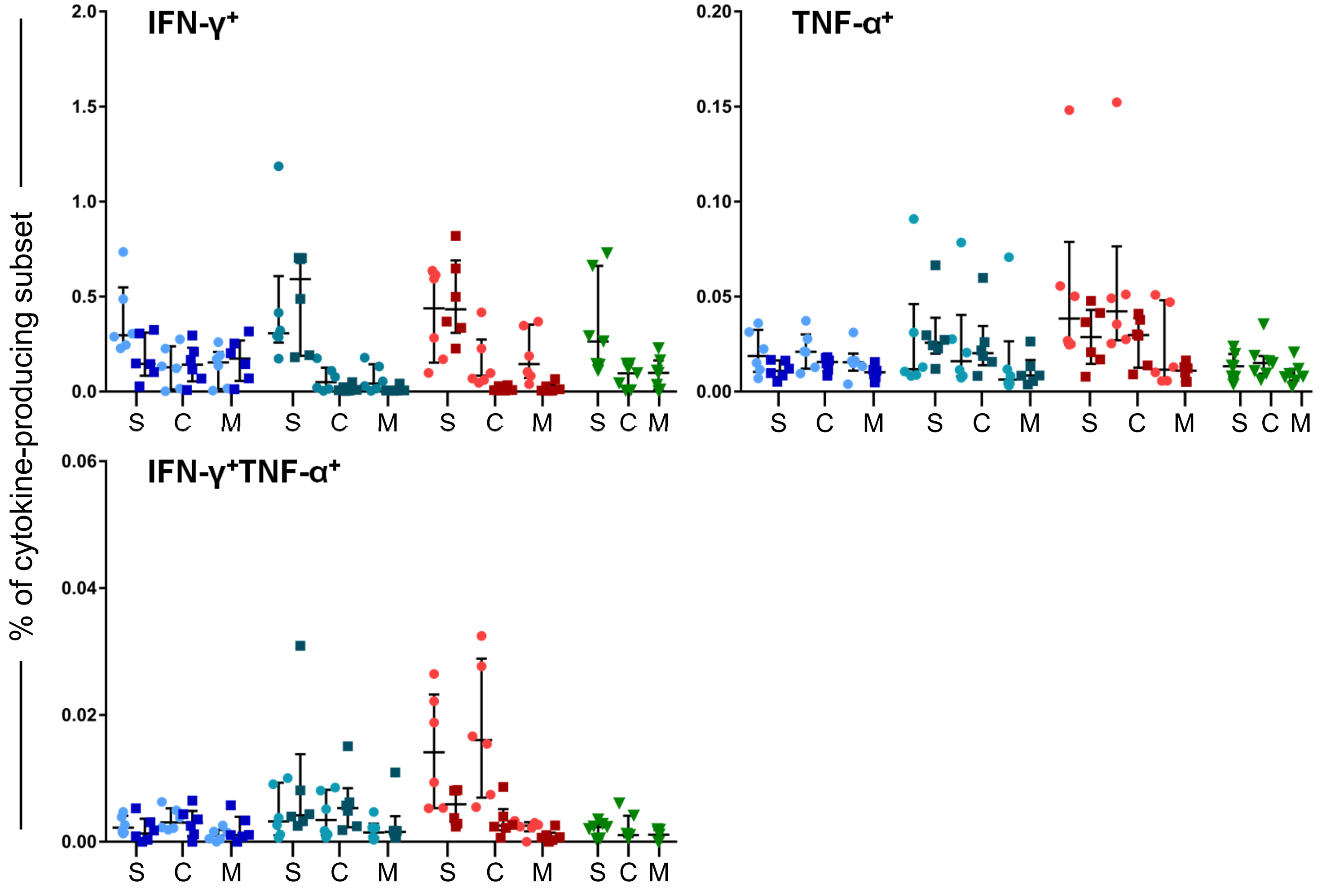

Figure S6E: CD8$^+$ T cells; Jejunum

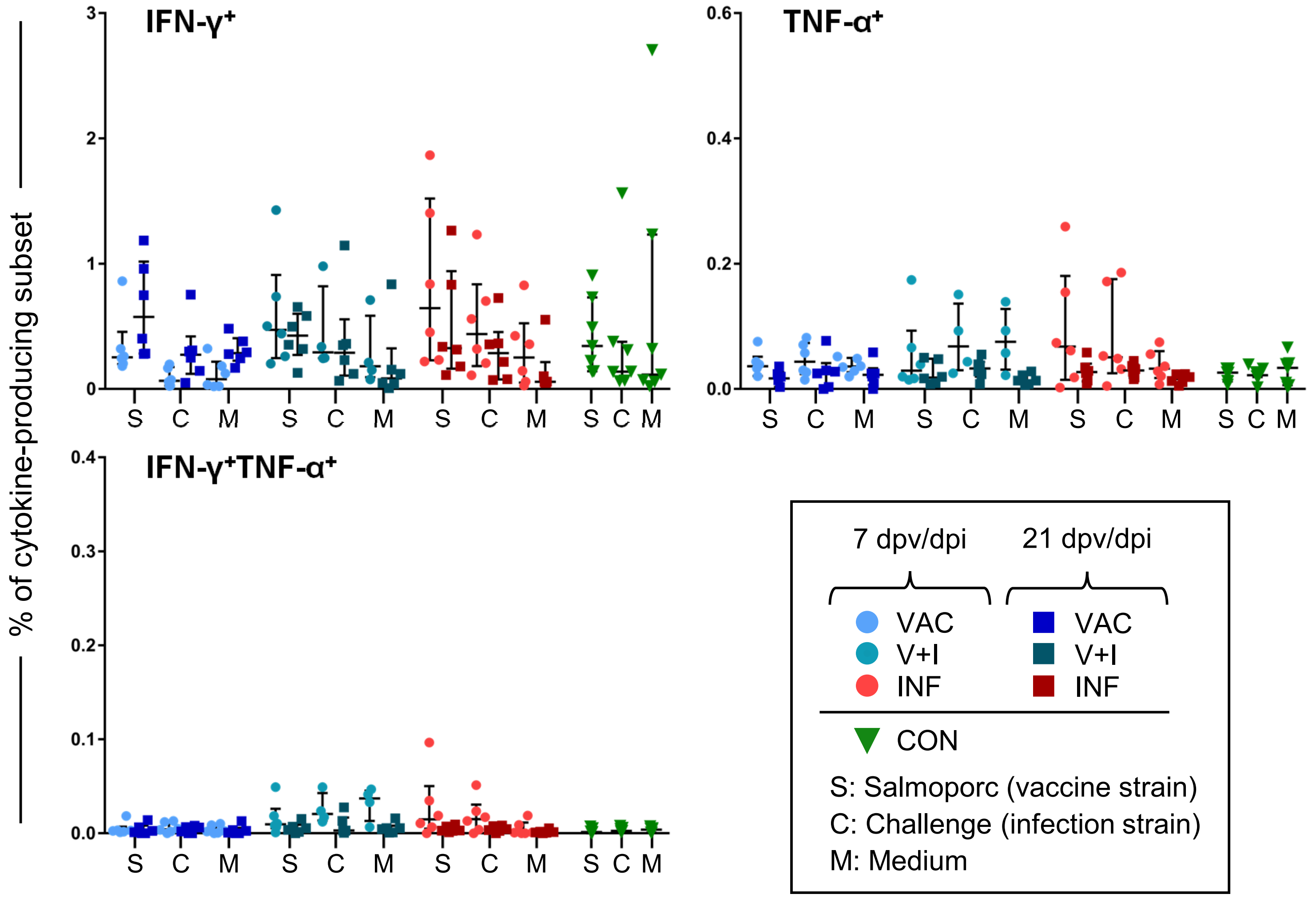

Figure S6F: CD8$^+$ T cells; Ileum

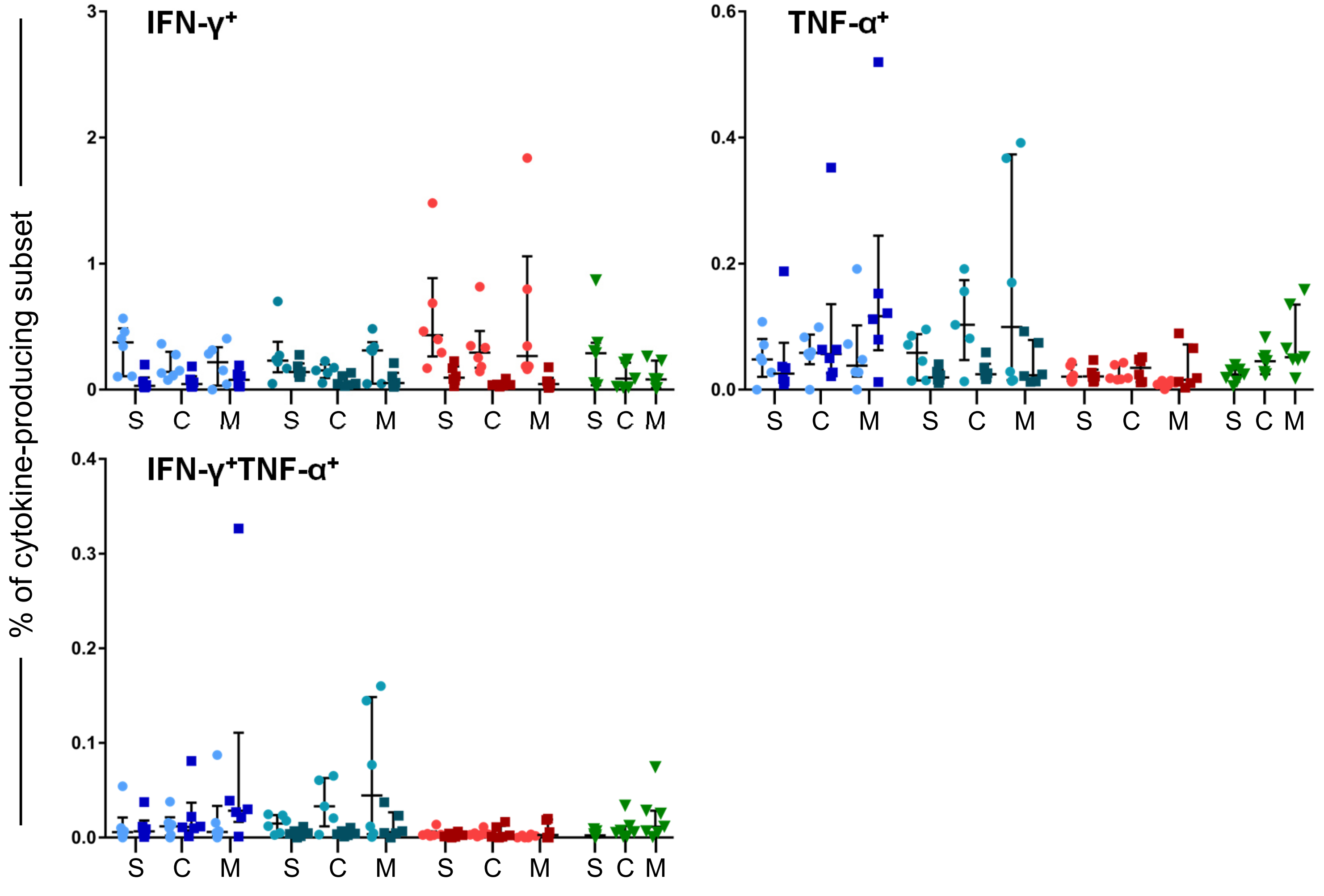

Figure S7A: CD4⁻CD8β⁻ T cells; Blood

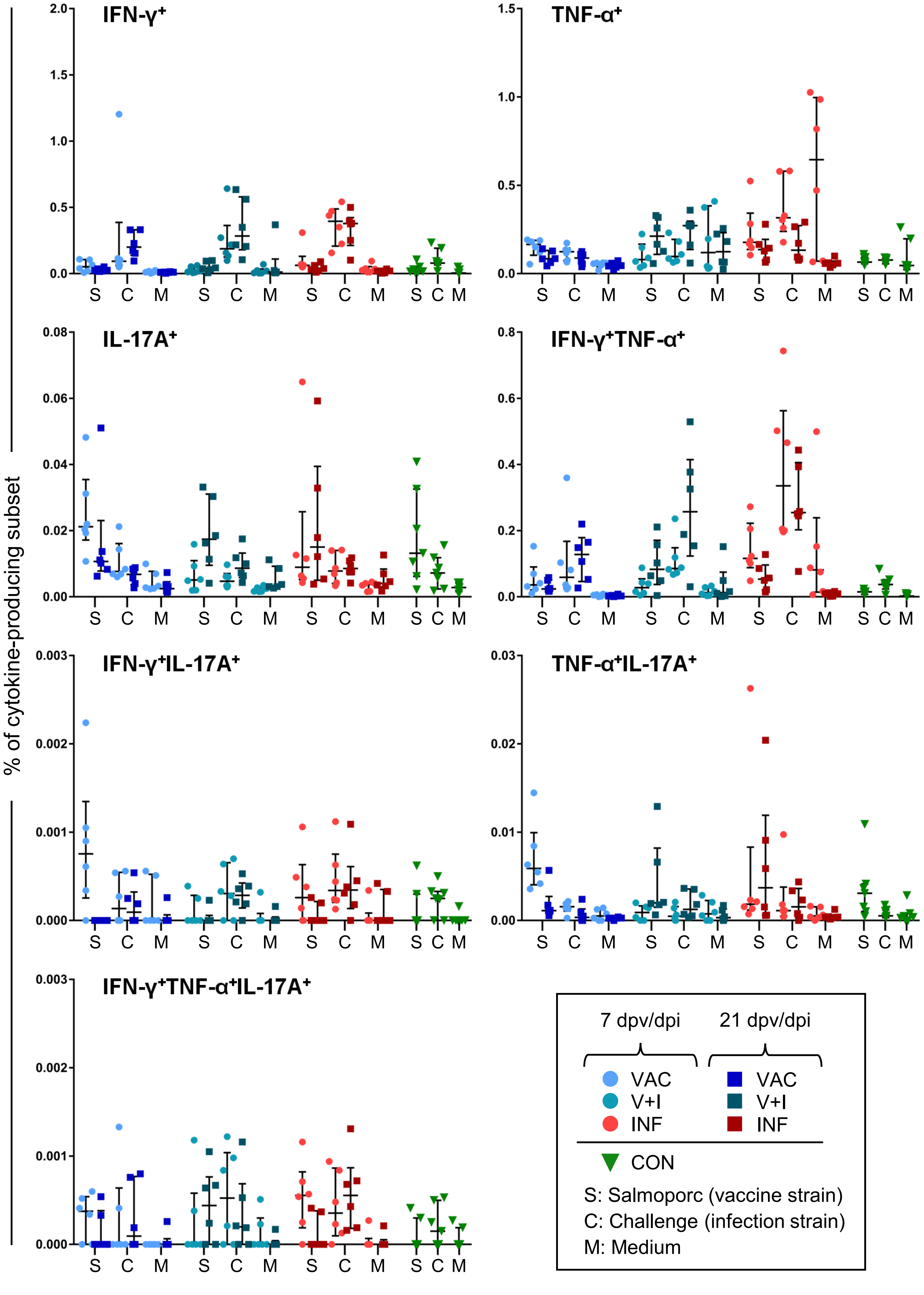

Figure S7B: CD4⁻CD8β⁻ T cells; Spleen

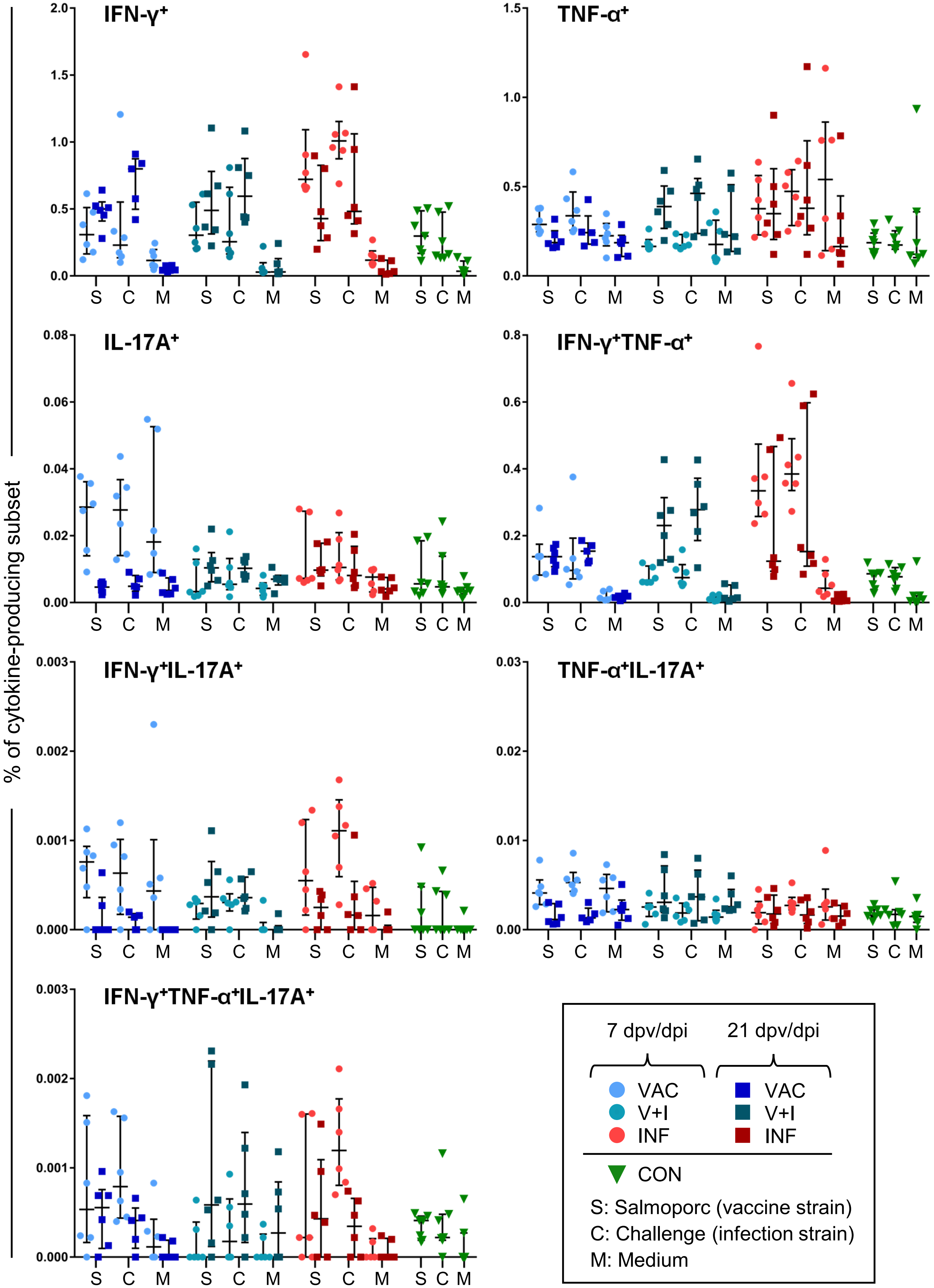

Figure S7C: CD4⁻CD8β⁻ T cells; JLN

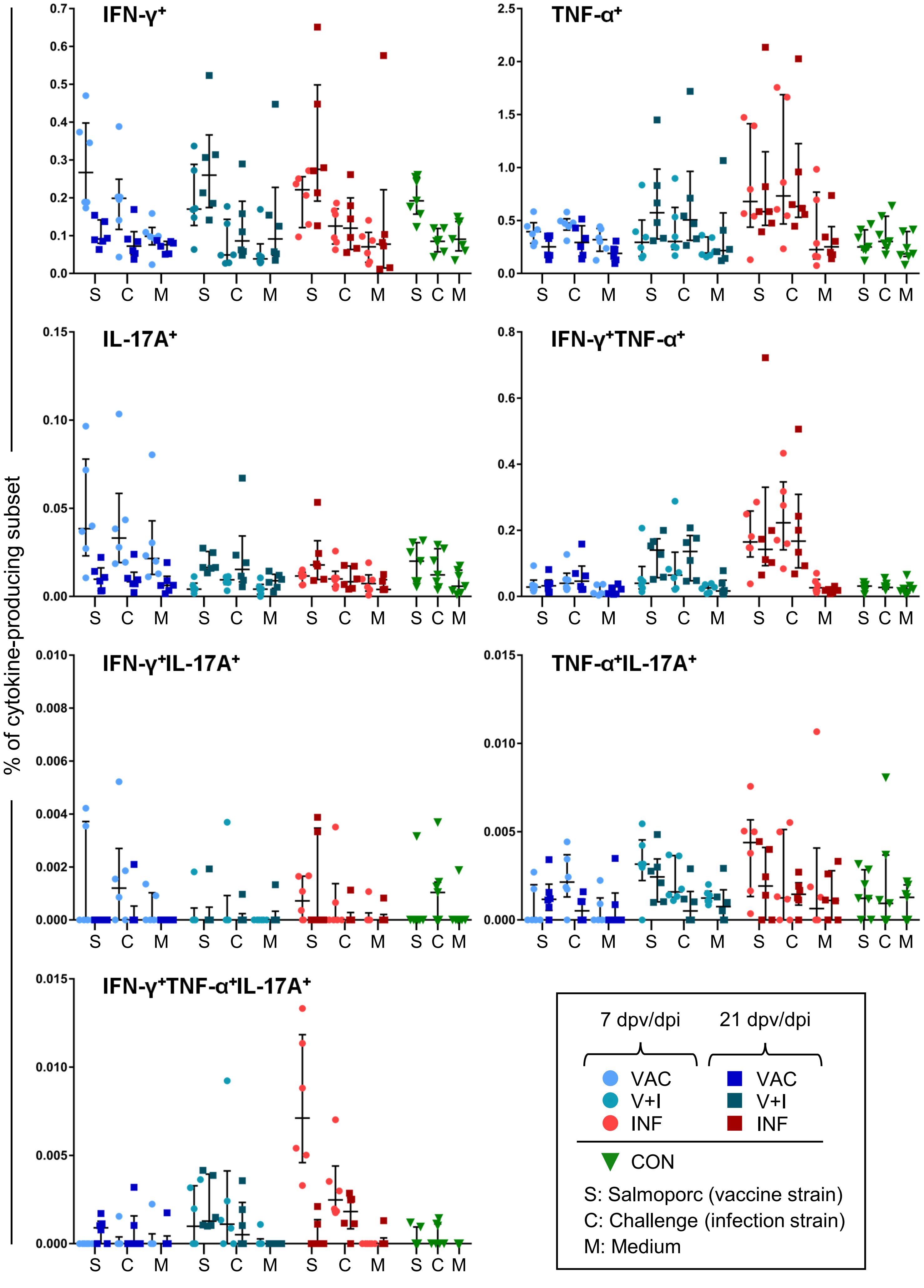

Figure S7D: CD4⁻CD8β⁻ T cells; ICLN

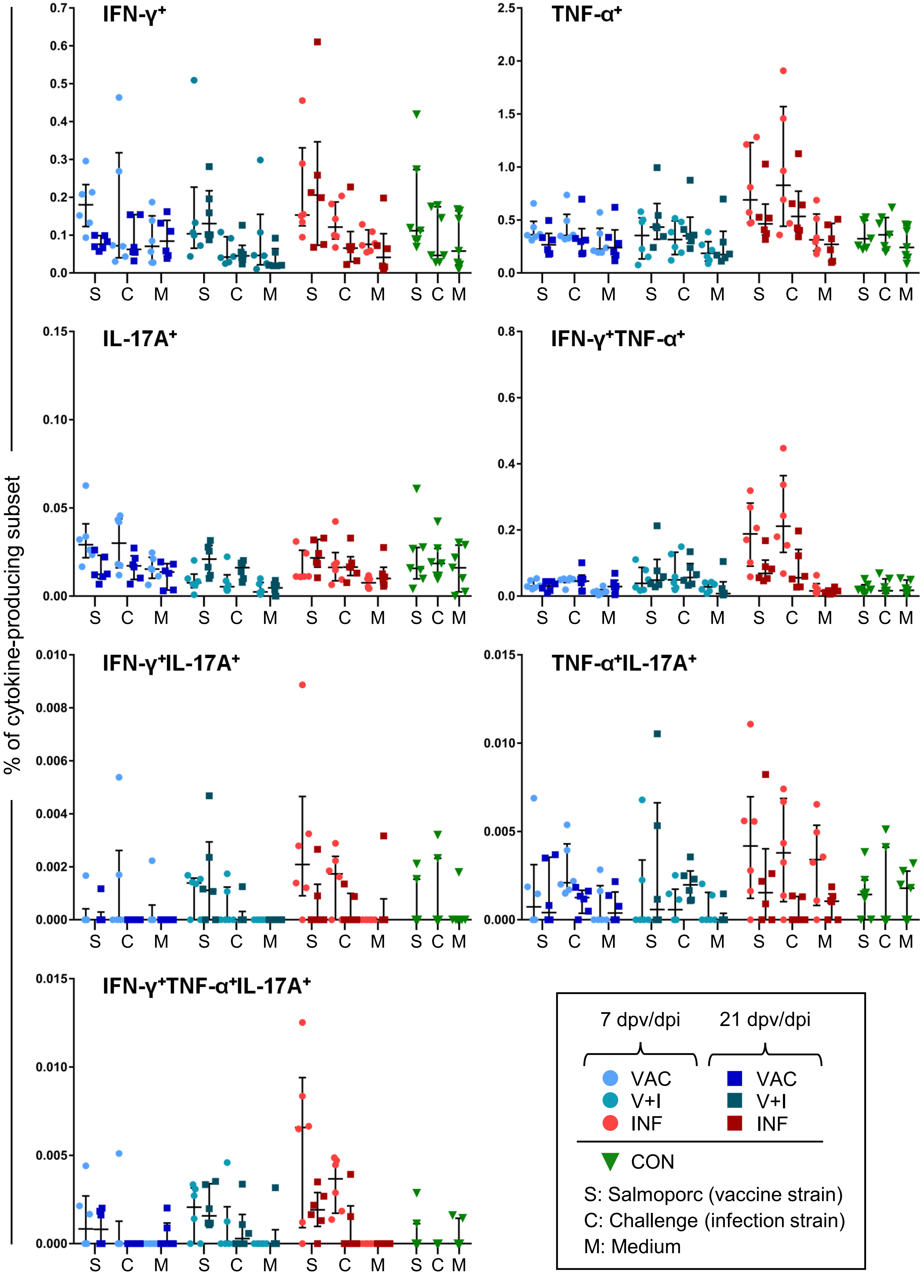

Figure S7E: CD4⁻CD8β⁻ T cells; Jejunum

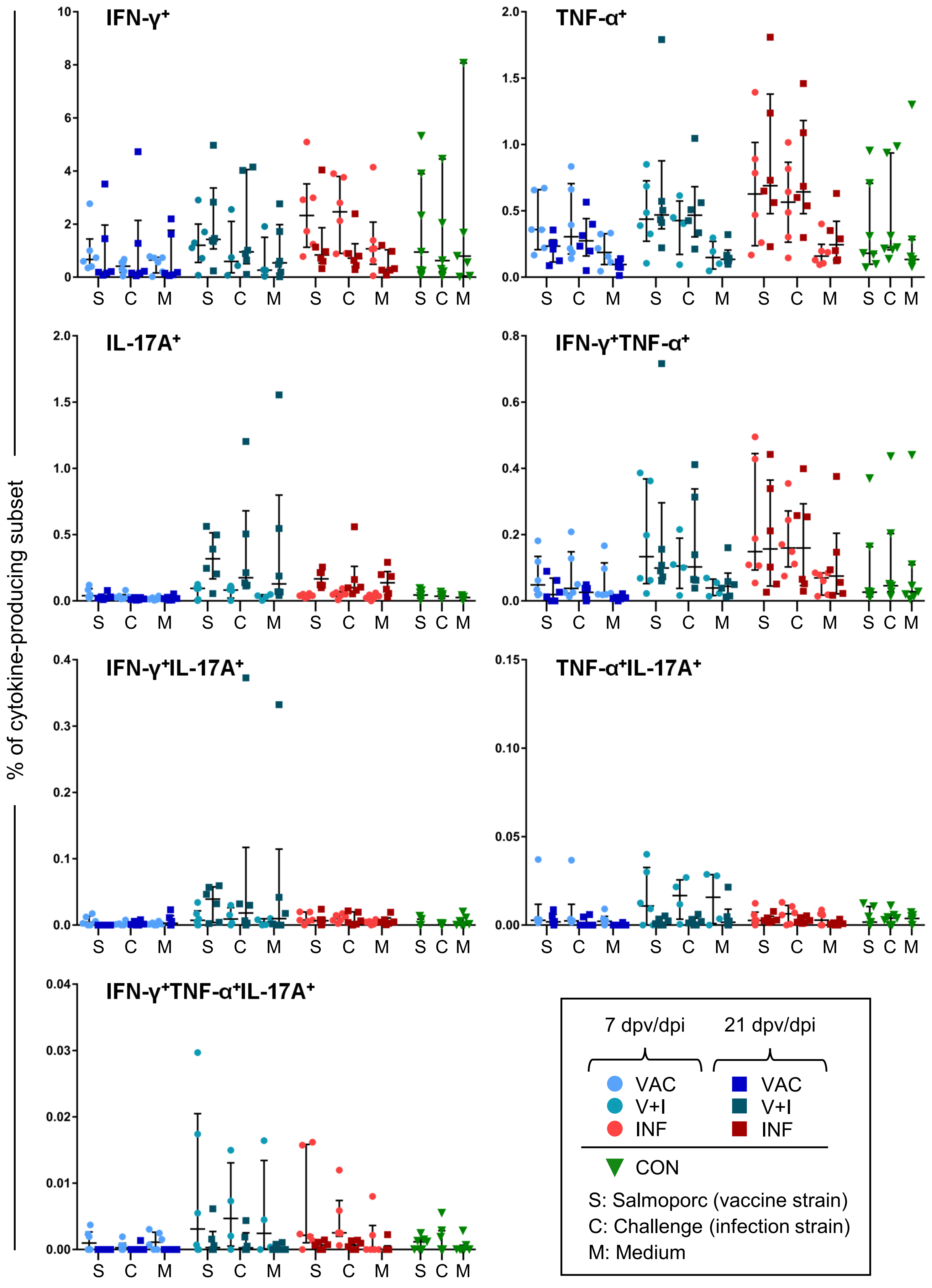

Figure S7F: CD4⁻CD8β⁻ T cells; Ileum

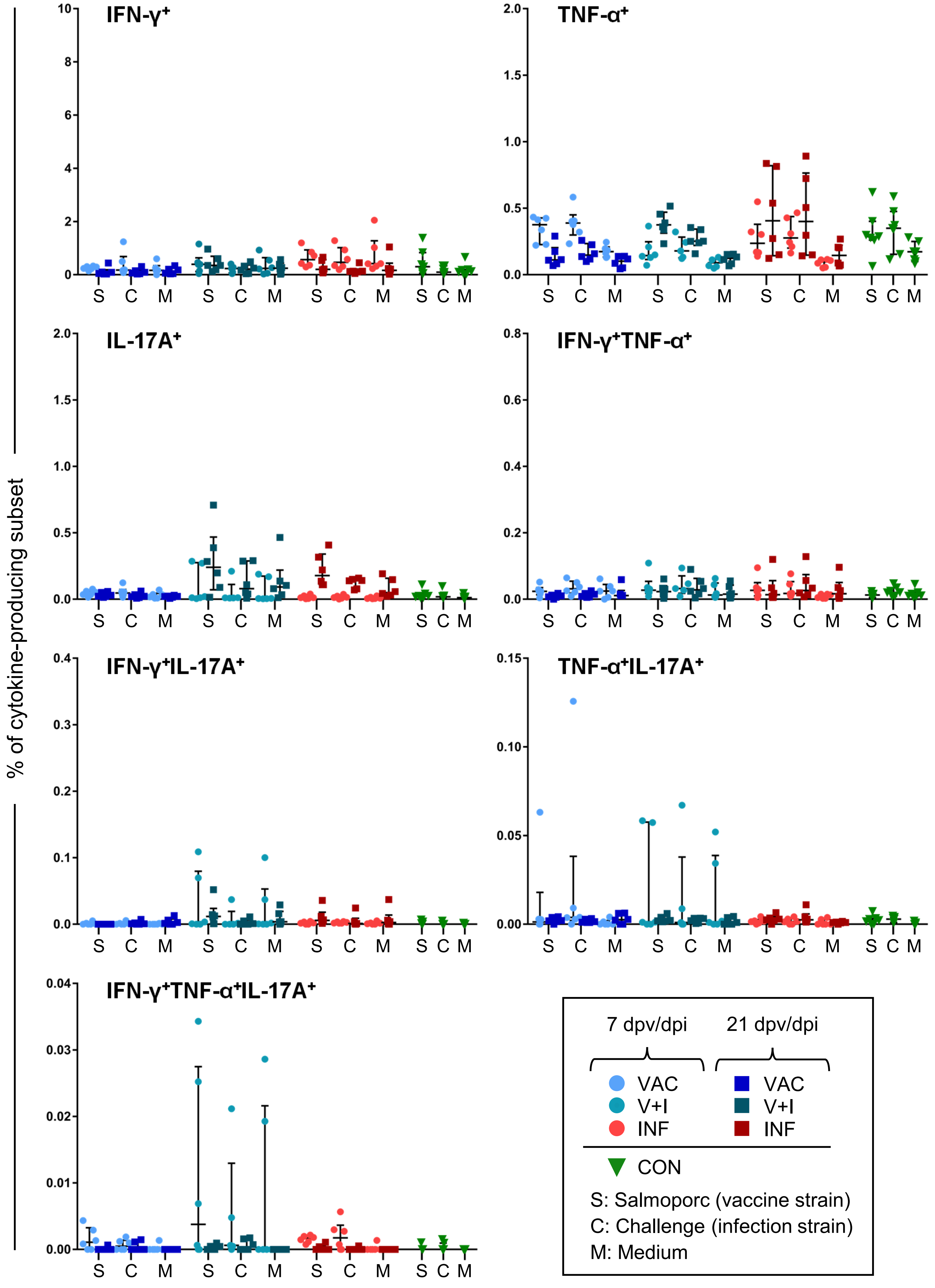

Figure S8A: CD4$^+$ T cells; Blood

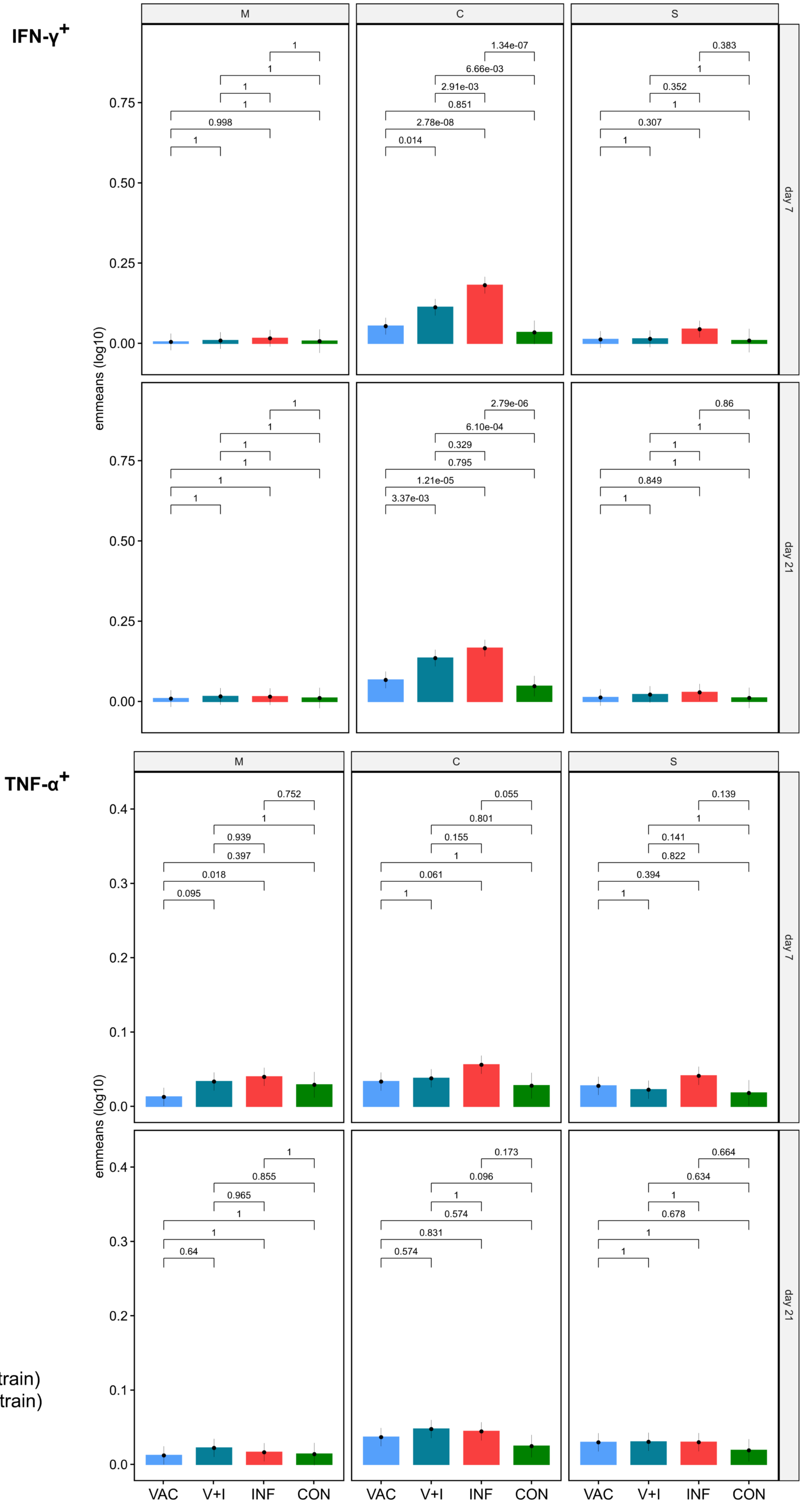

VAC
V+I
INF
CON

S: Salmoporc (vaccine strain)
C: Challenge (infection strain)
M: Medium

day 7: 7 dpv/dpi
day 21: 21 dpv/dpi

Figure S8A: CD4^{+} T cells; Blood

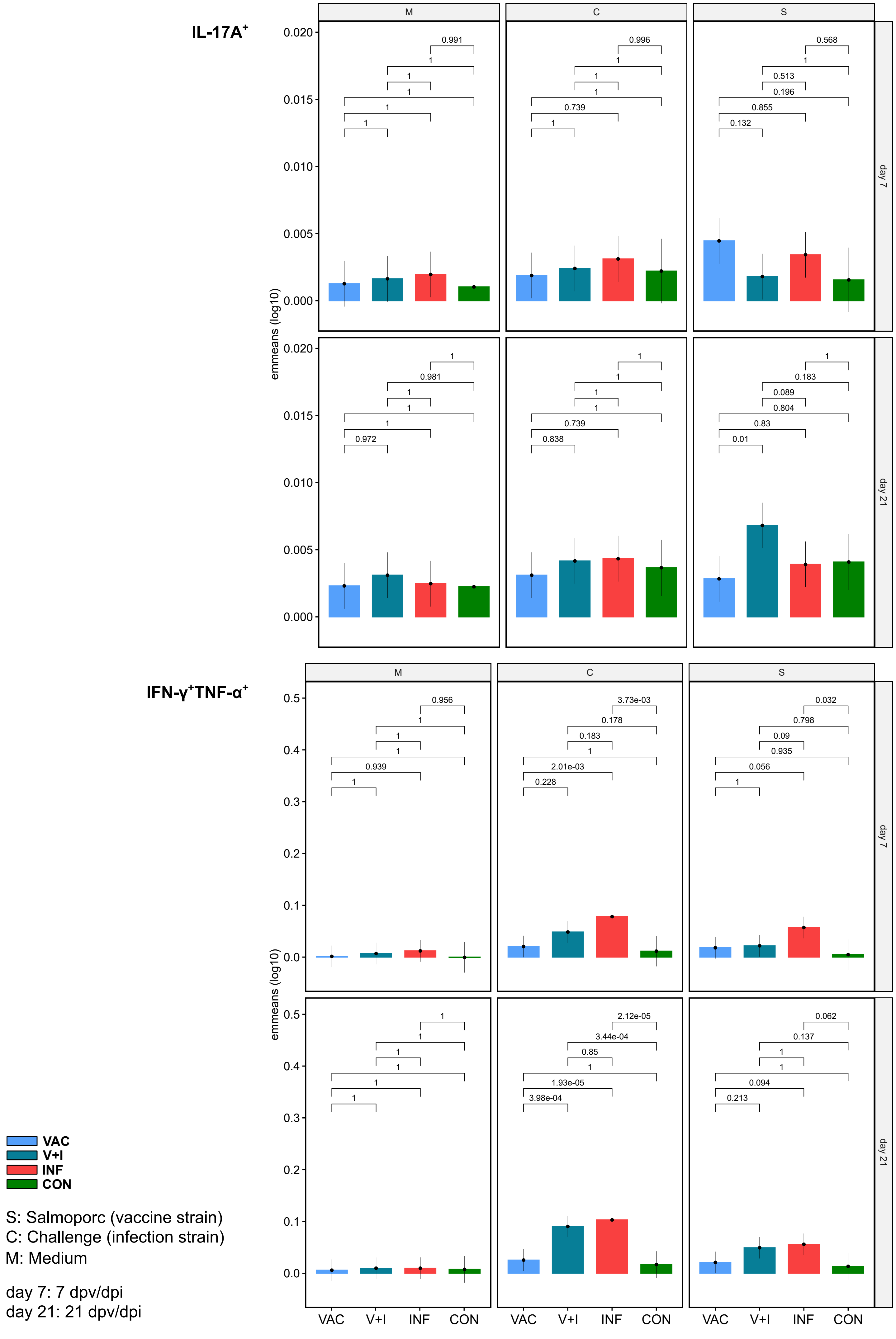

Figure S8A: CD4^{+} T cells; Blood

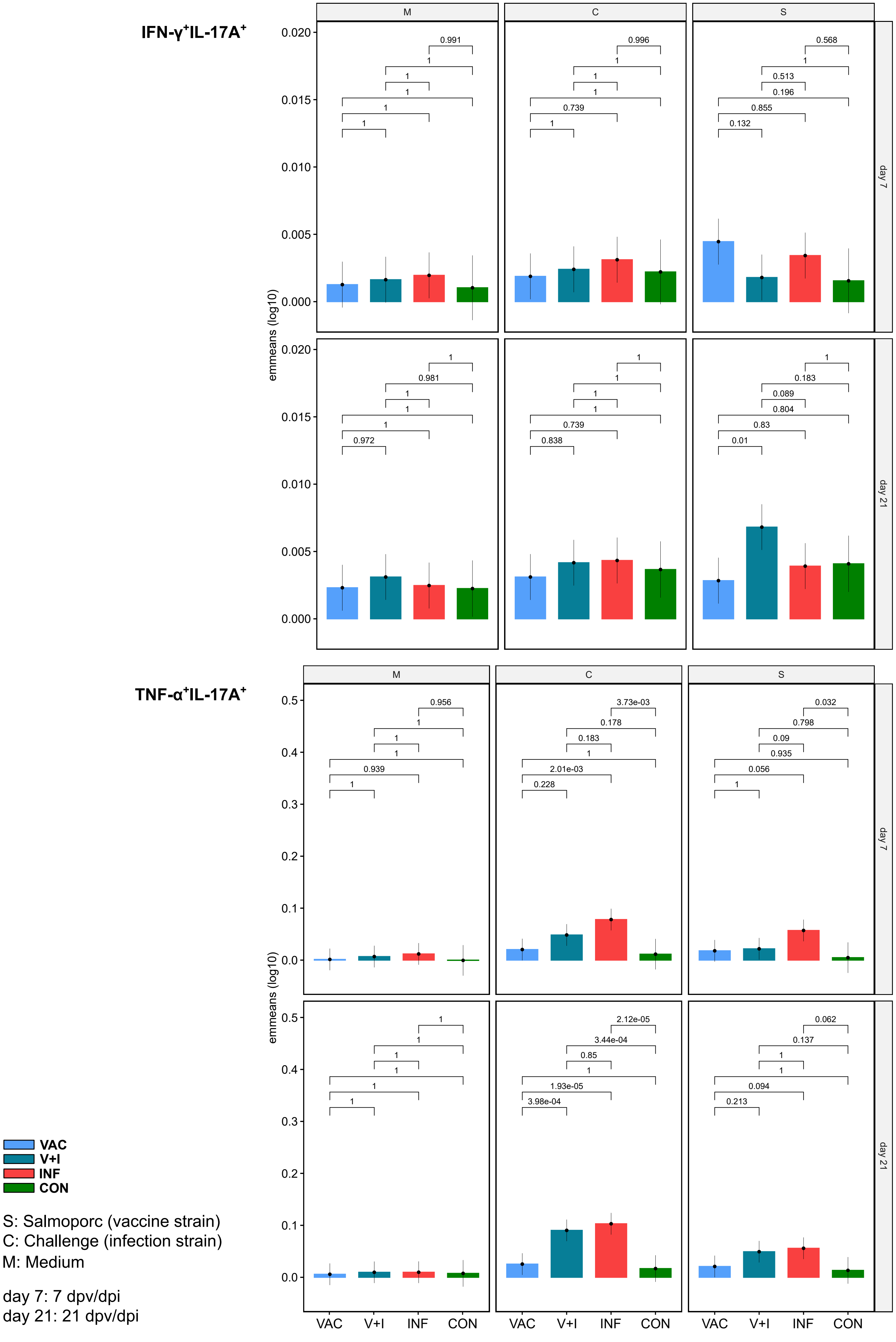

Figure S8A: CD4^{+} T cells; Blood

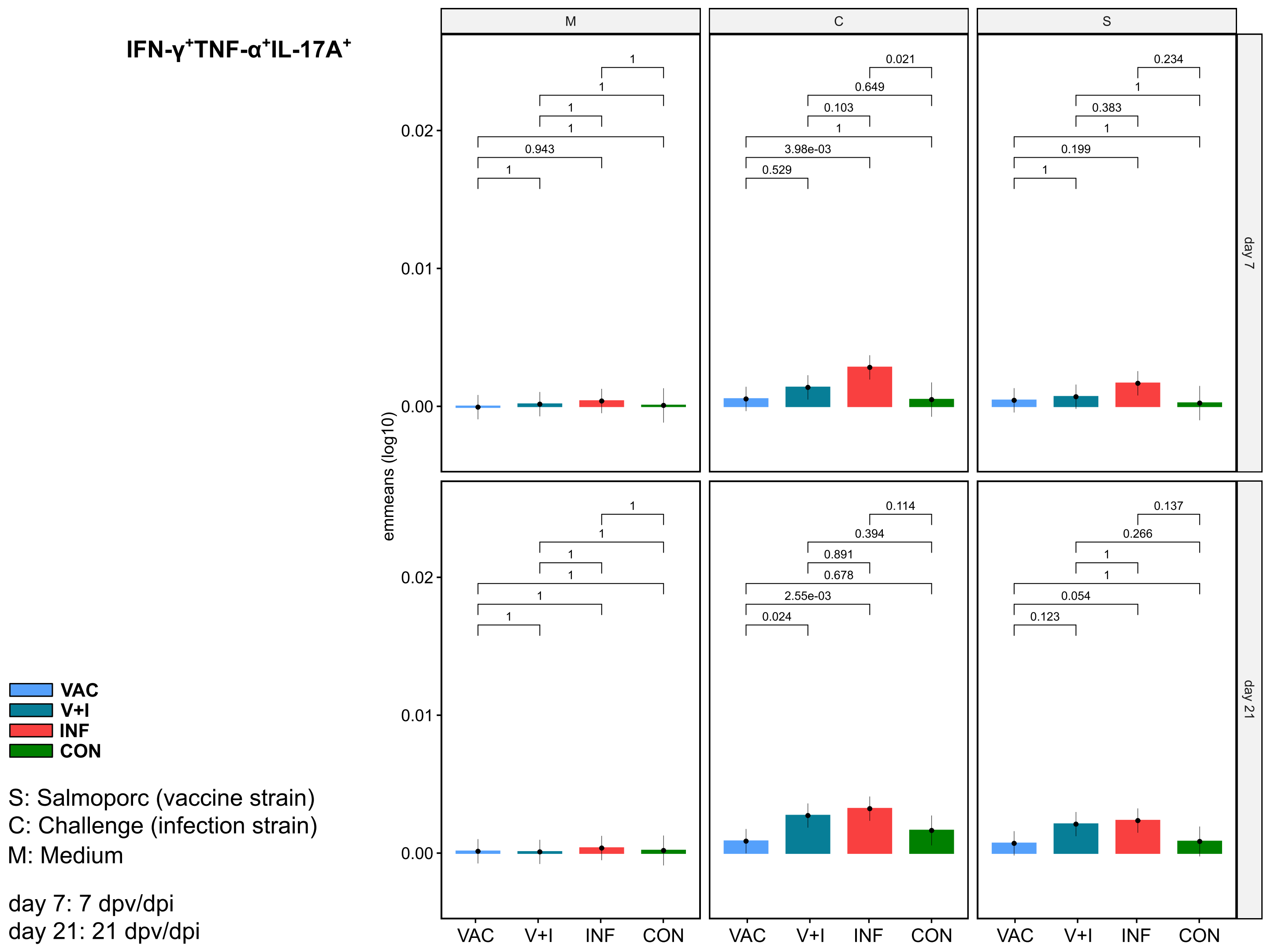

Figure S8B: CD4+ T cells; Spleen

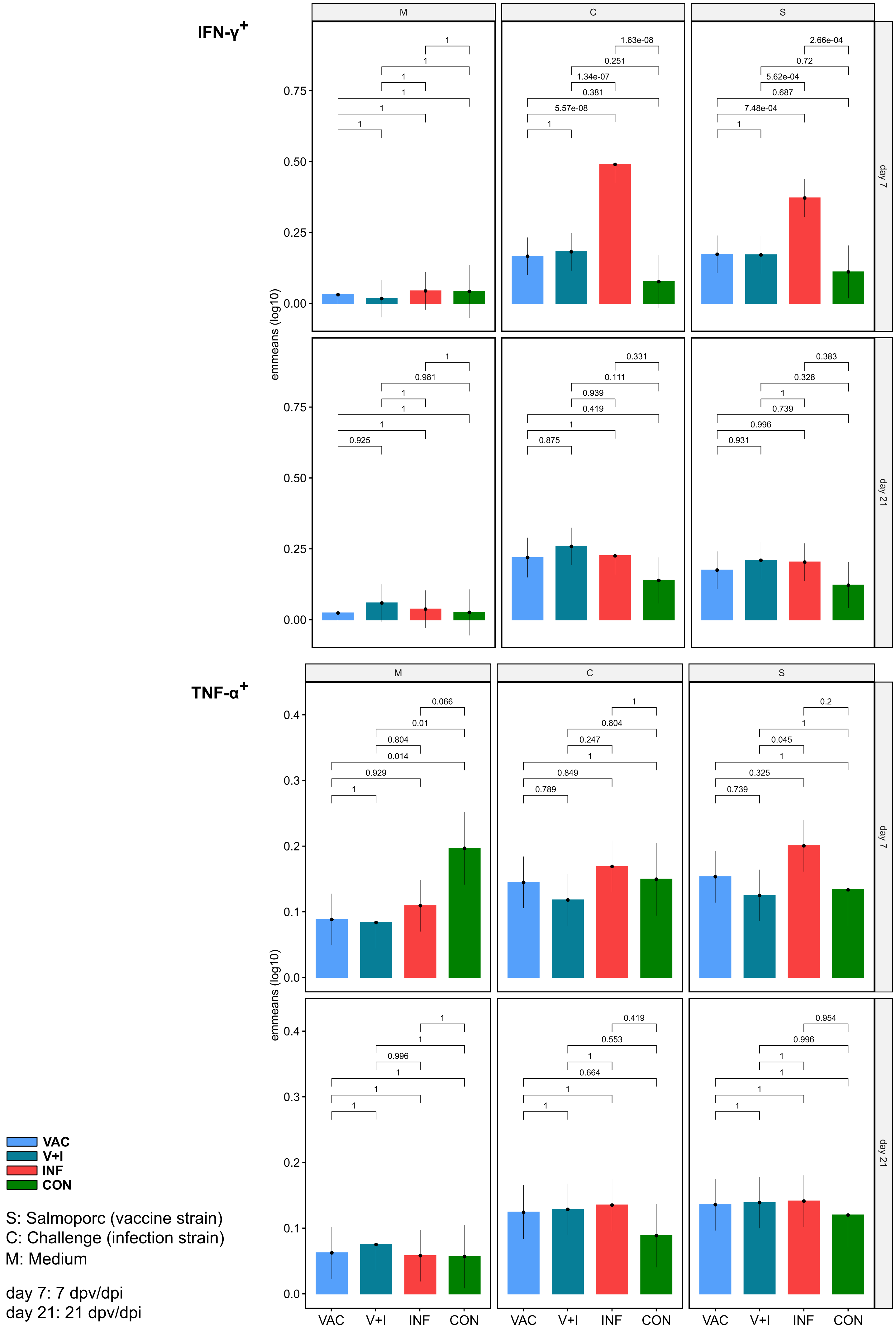

Figure S8B: CD4^{+} T cells; Spleen

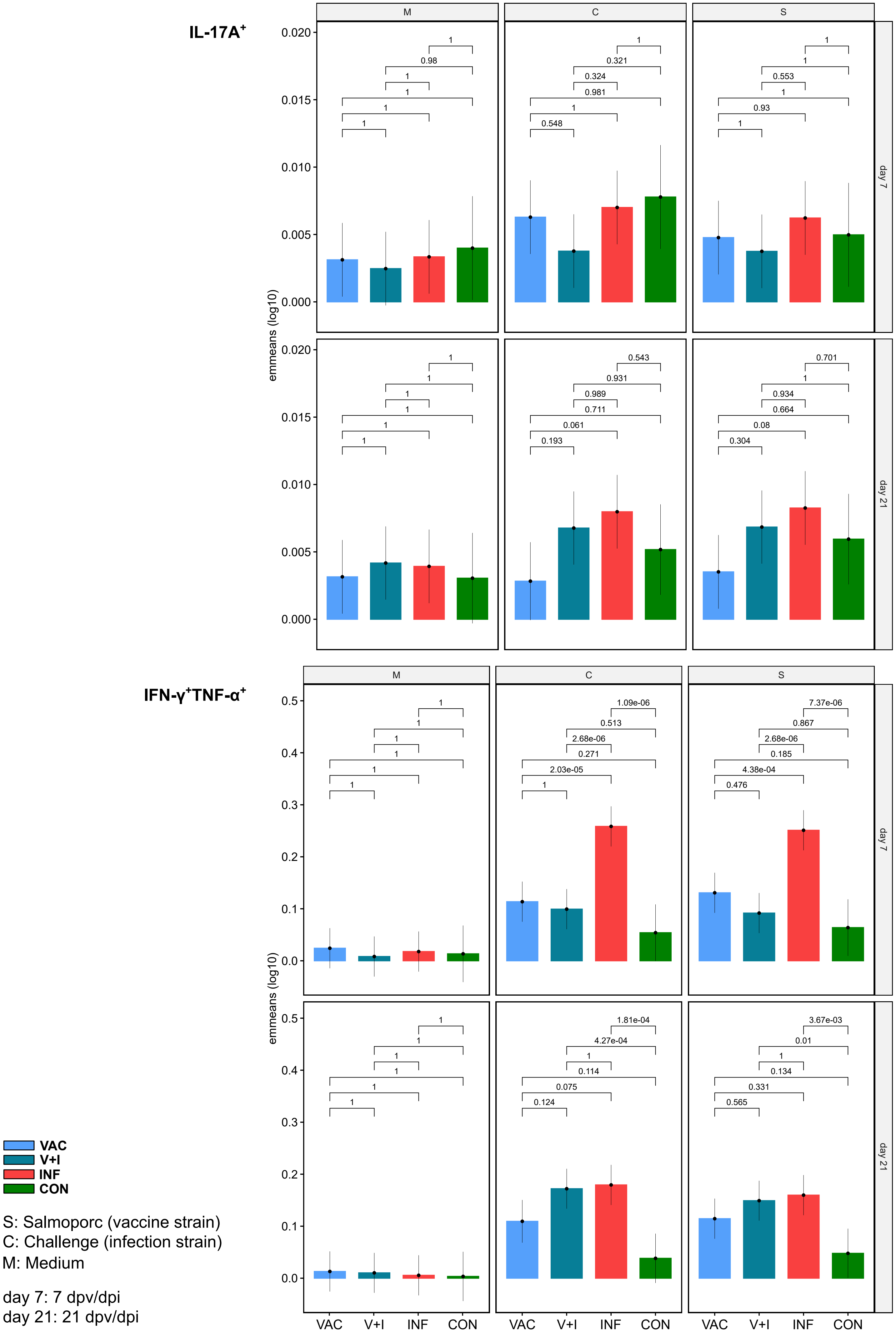

Figure S8B: CD4+ T cells; Spleen

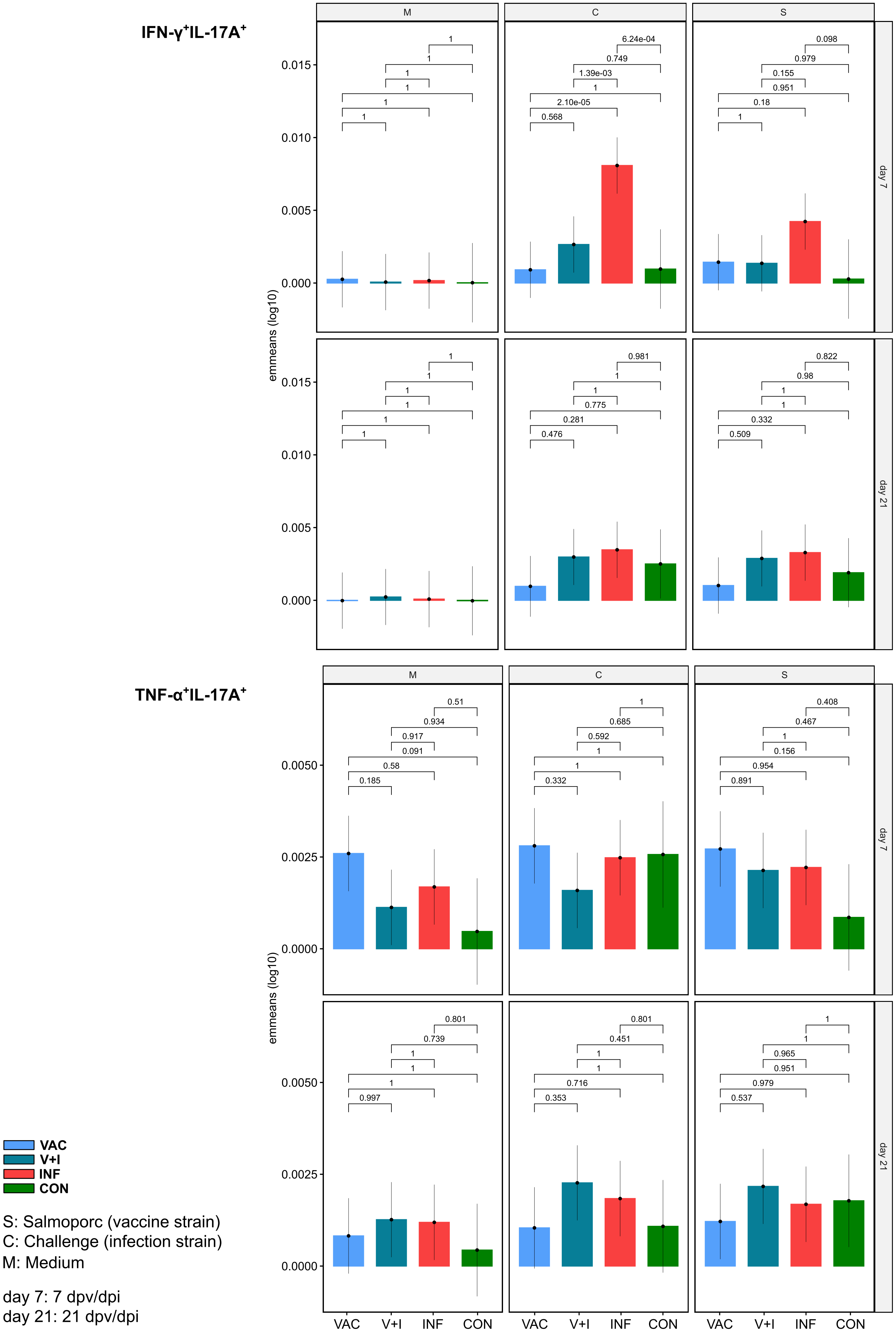

Figure S8B: CD4^{+} T cells; Spleen

IFN-γ^{+}TNF-α^{+}IL-17A^{+}

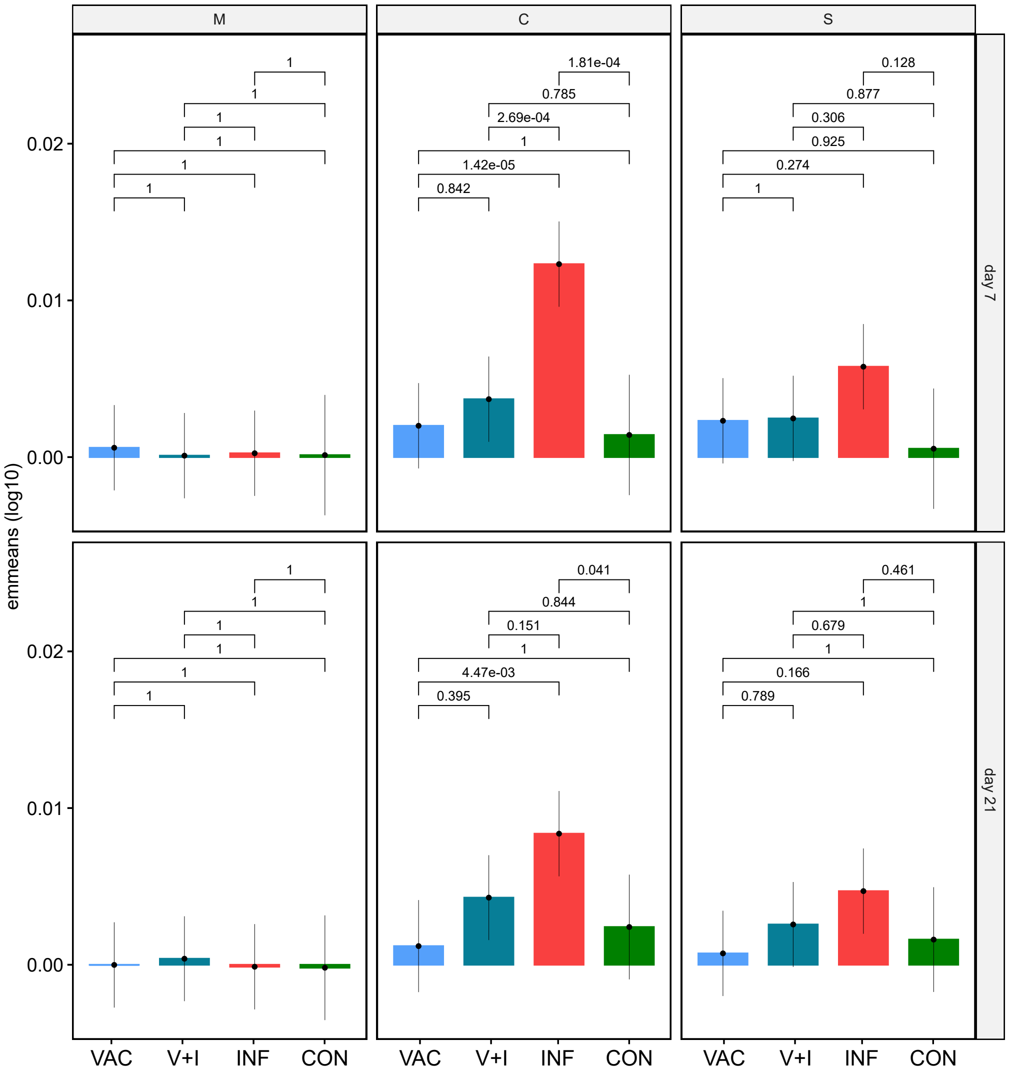

VAC
V+I
INF
CON

S: Salmoporc (vaccine strain)
C: Challenge (infection strain)
M: Medium

day 7: 7 dpv/dpi
day 21: 21 dpv/dpi

Figure S8C: CD4$^+$ T cells; JLN

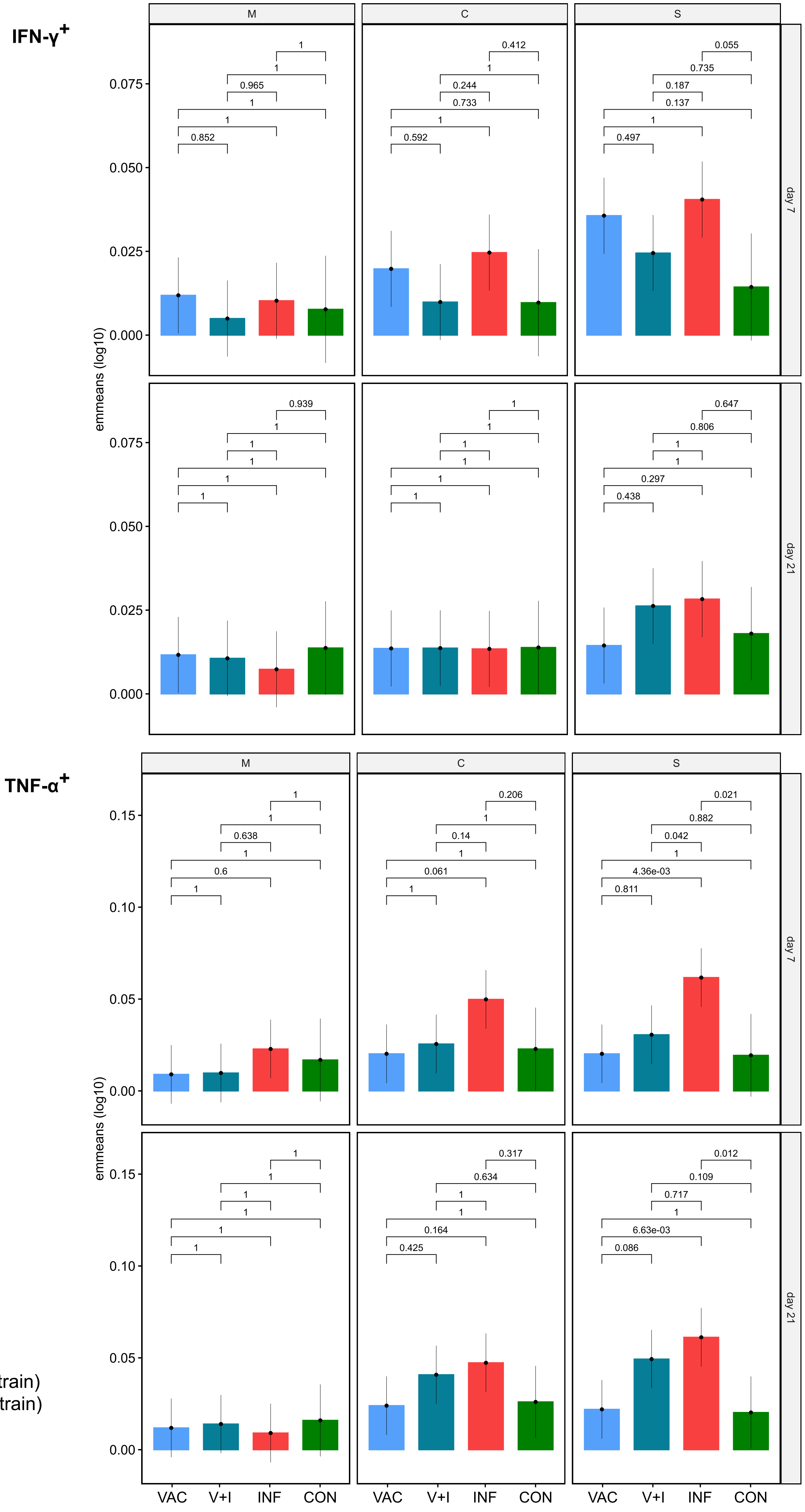

VAC
V+I
INF
CON

S: Salmoporc (vaccine strain)
C: Challenge (infection strain)
M: Medium

day 7: 7 dpv/dpi
day 21: 21 dpv/dpi

Figure S8C: CD4^{+} T cells; JLN

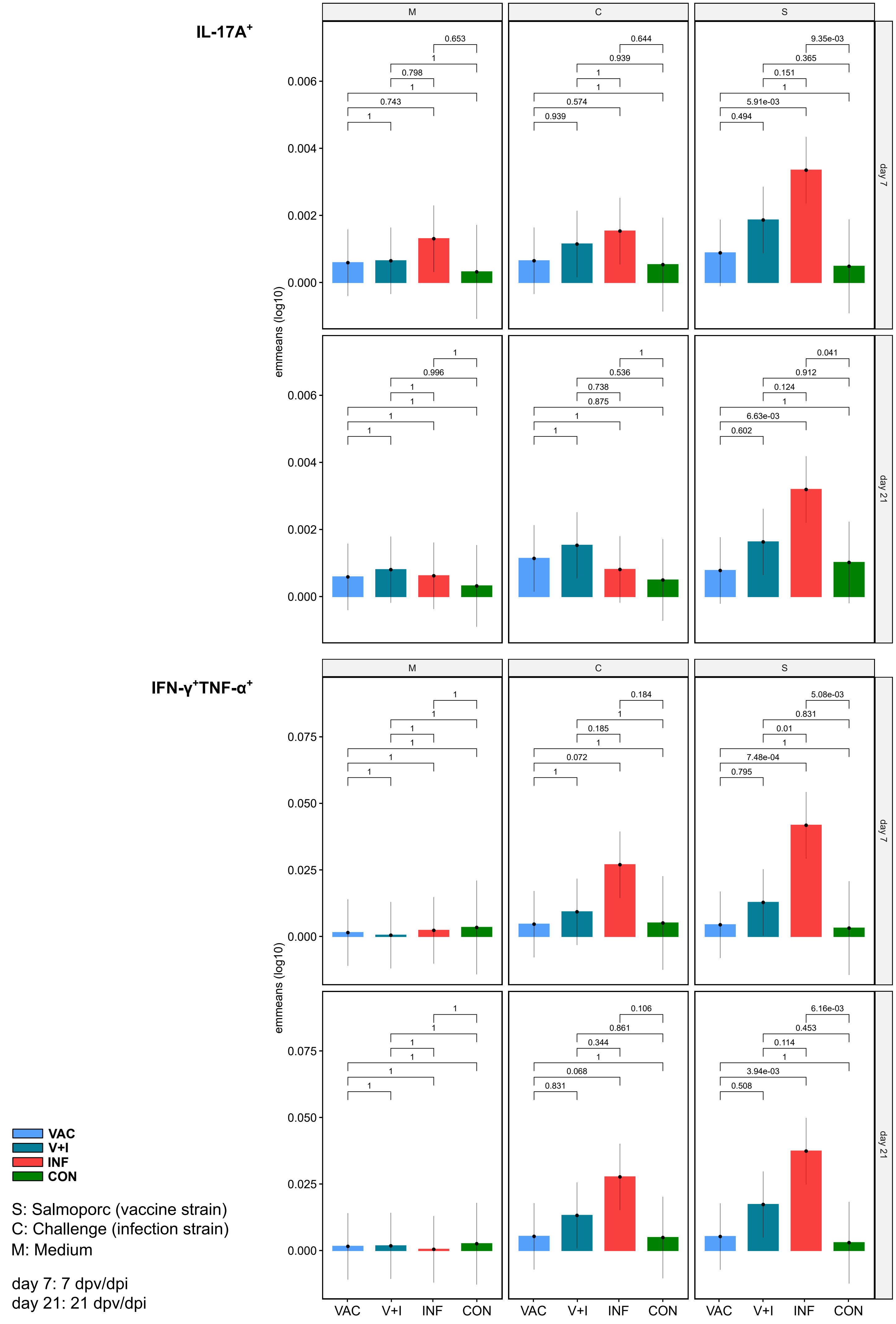

Figure S8C: CD4$^+$ T cells; JLN

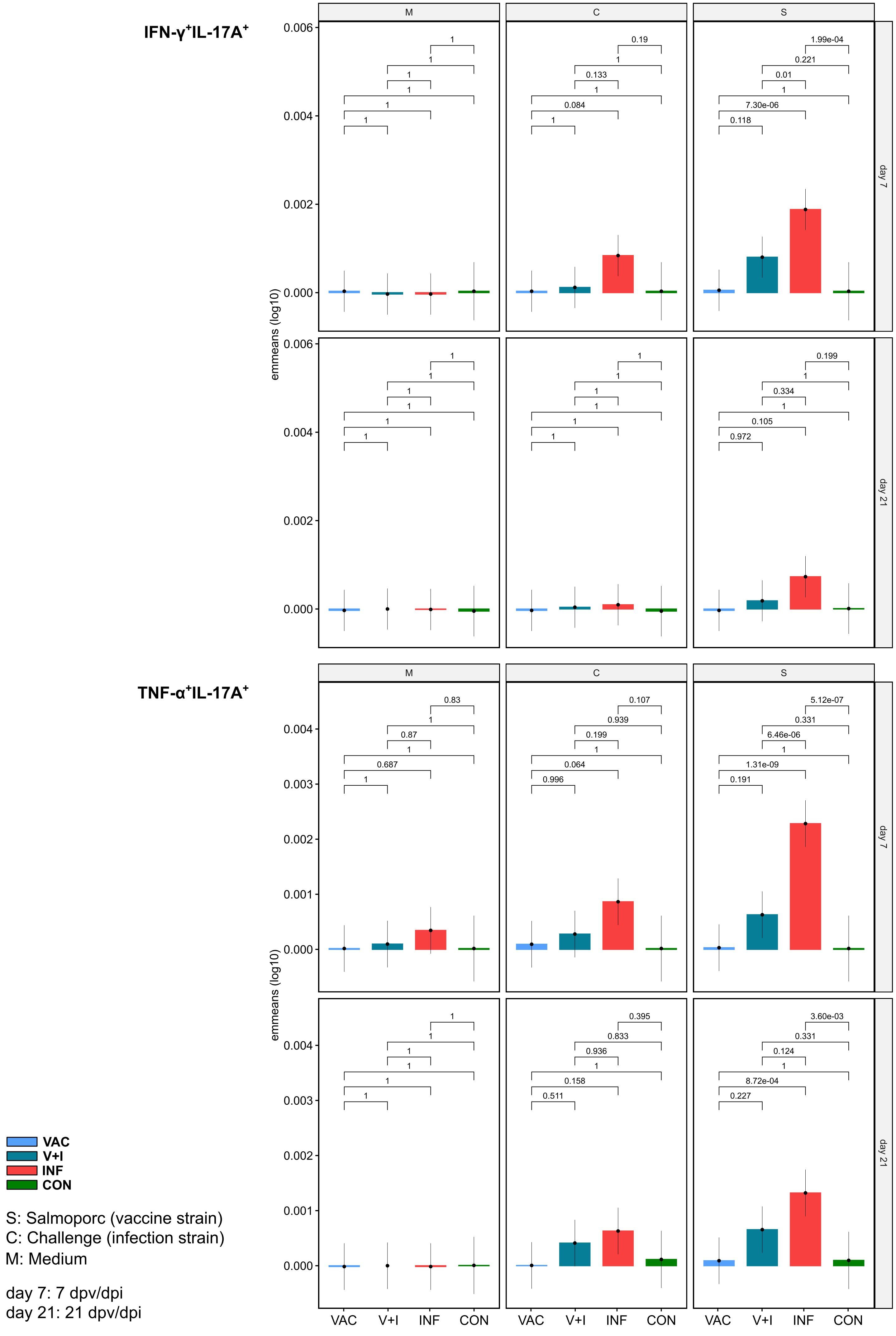

Figure S8C: CD4⁺ T cells; JLN

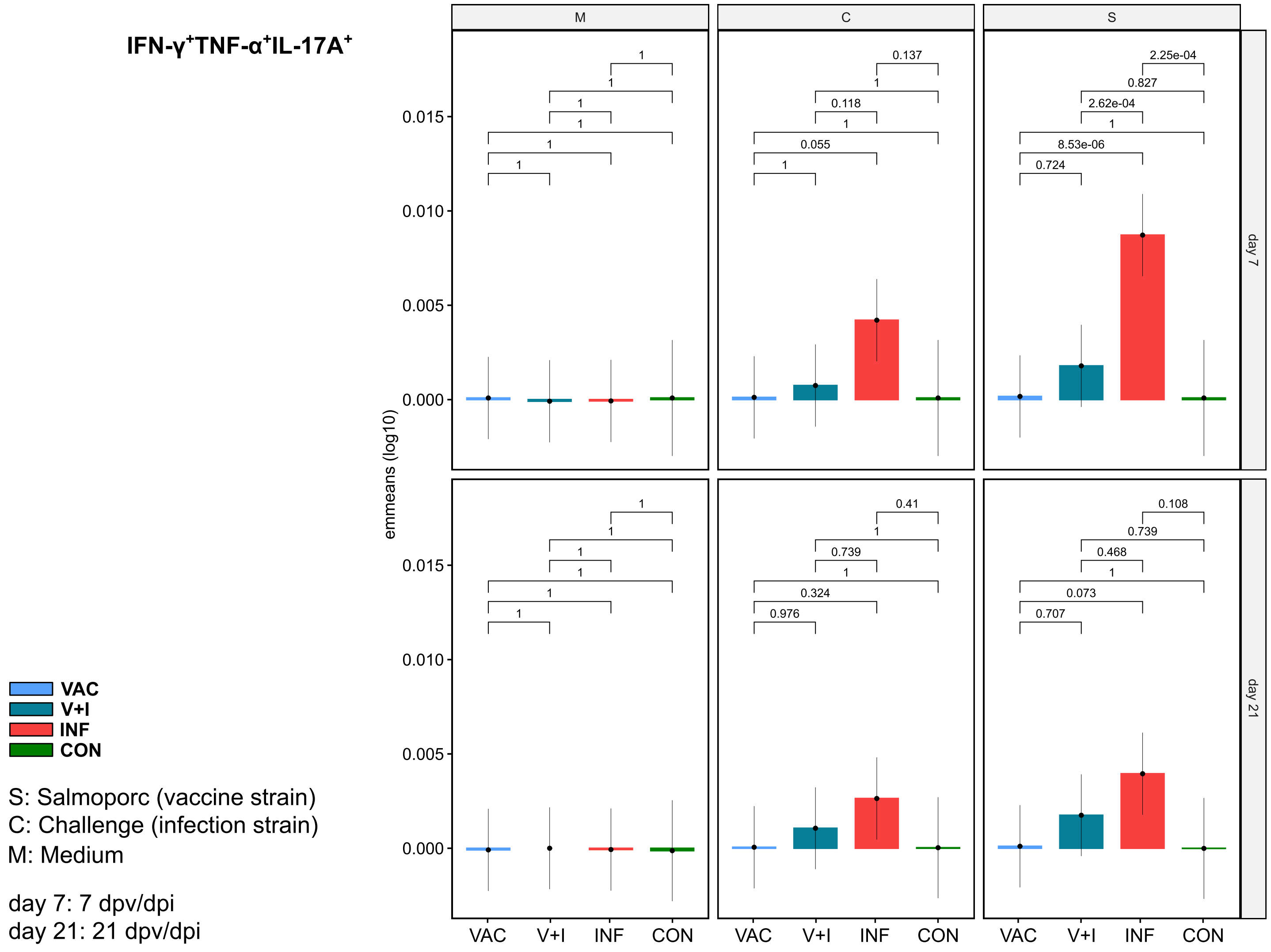

Figure S8D: CD4+ T cells; ICLN

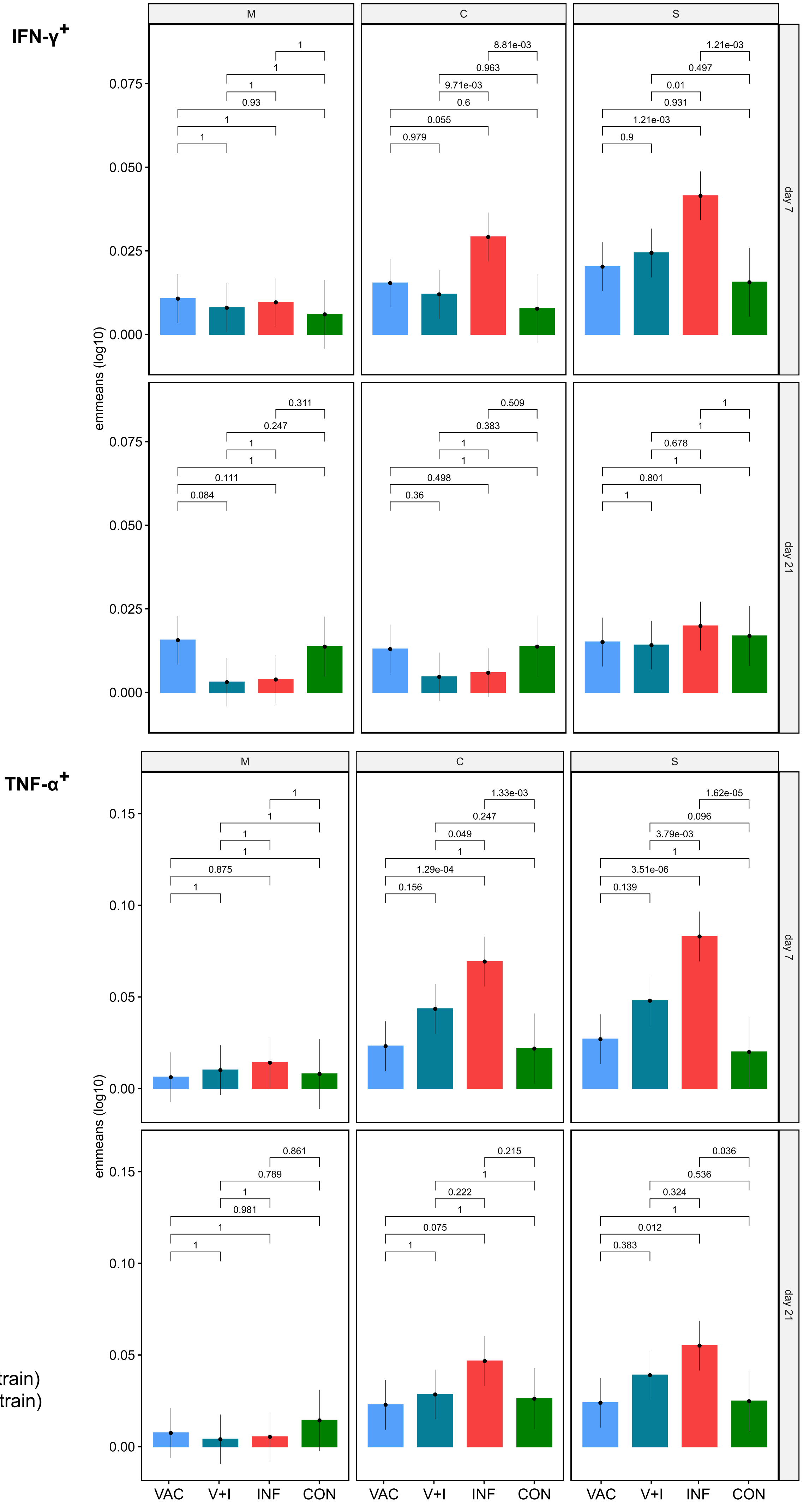

VAC
V+I
INF
CON

S: Salmoporc (vaccine strain)
C: Challenge (infection strain)
M: Medium

day 7: 7 dpv/dpi
day 21: 21 dpv/dpi

Figure S8D: CD4+ T cells; ICLN

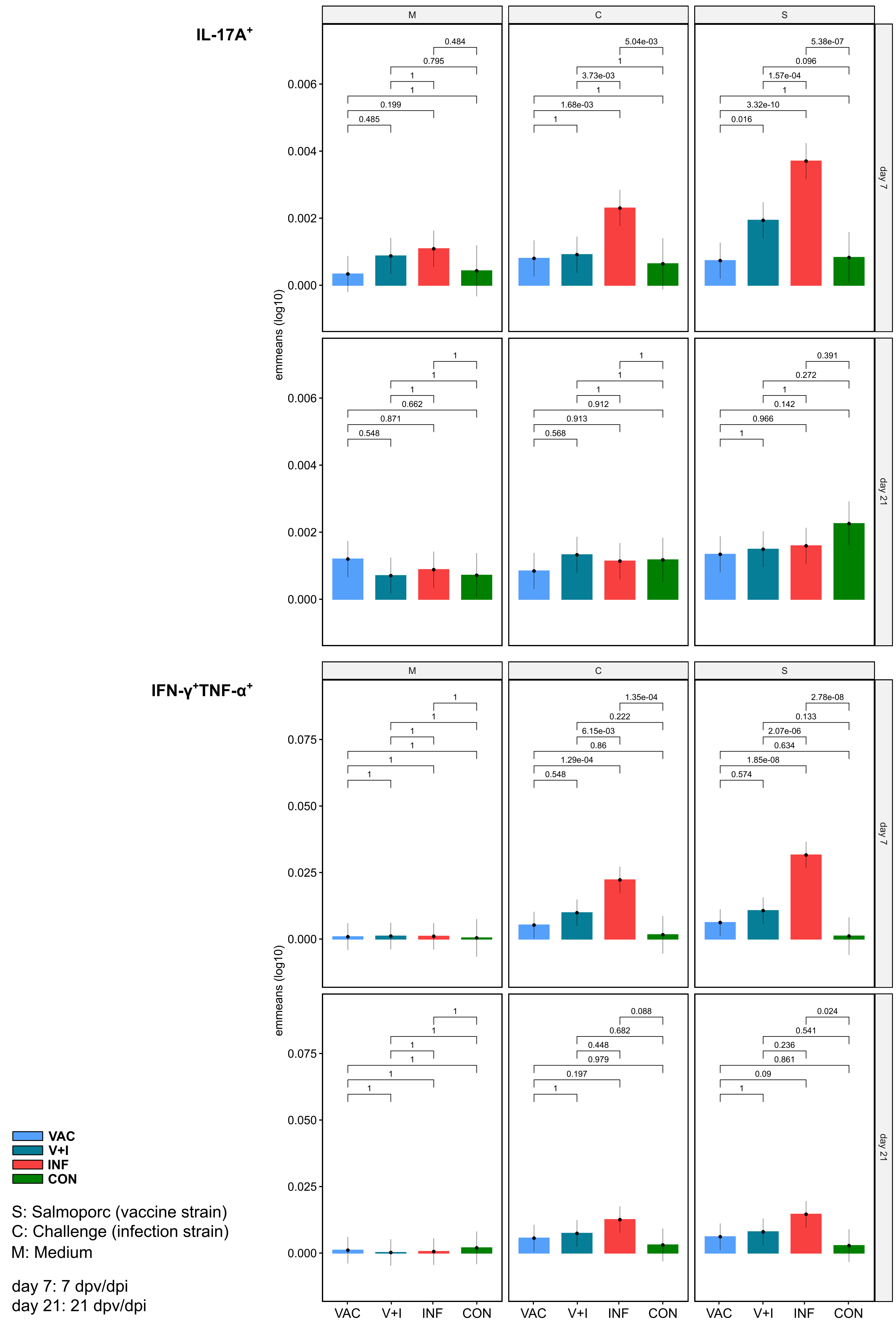

Figure S8D: CD4⁺ T cells; ICLN

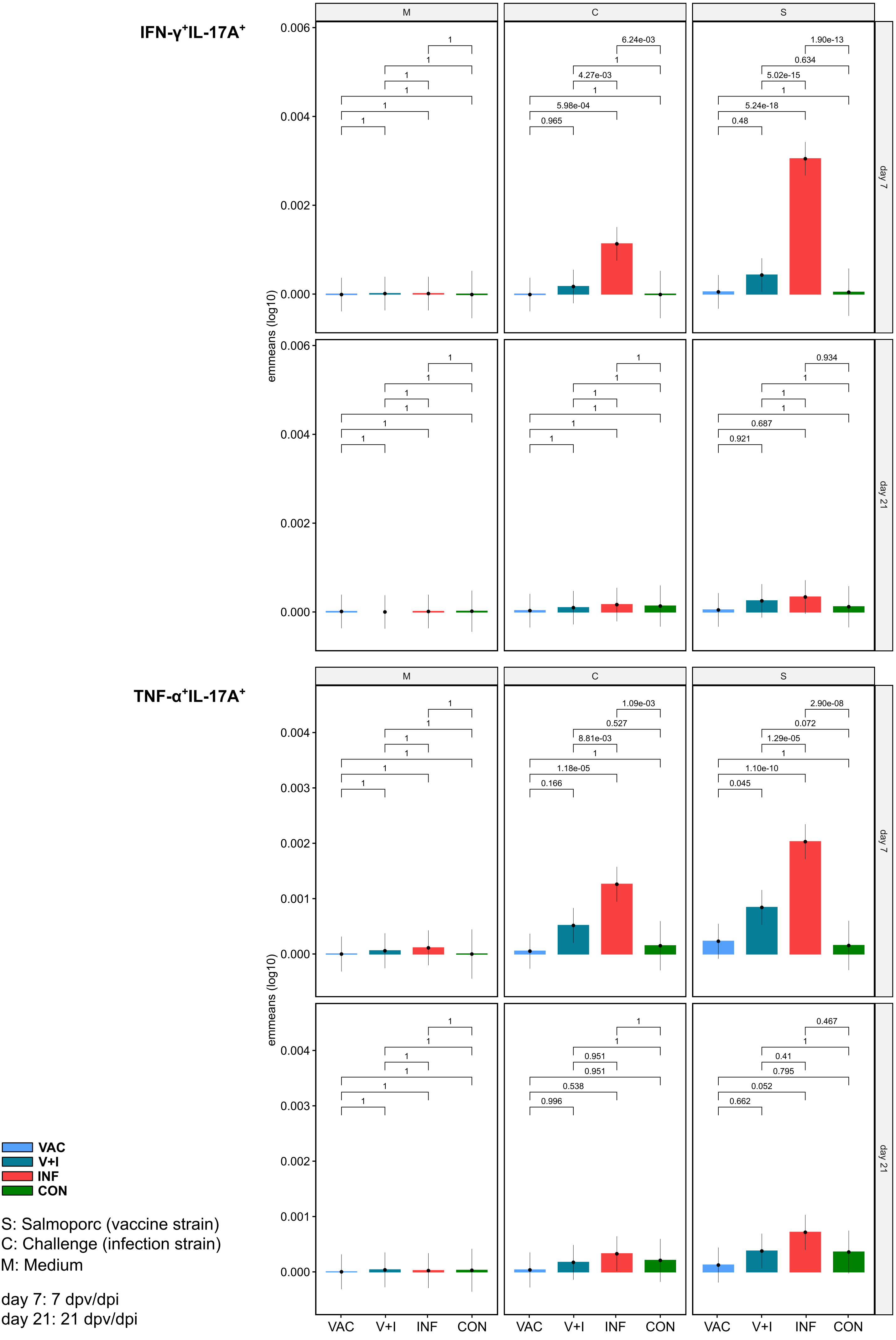

S: Salmoporc (vaccine strain)
C: Challenge (infection strain)
M: Medium

day 7: 7 dpv/dpi
day 21: 21 dpv/dpi

Figure S8D: CD4⁺ T cells; ICLN

IFN-γ⁺TNF-α⁺IL-17A⁺

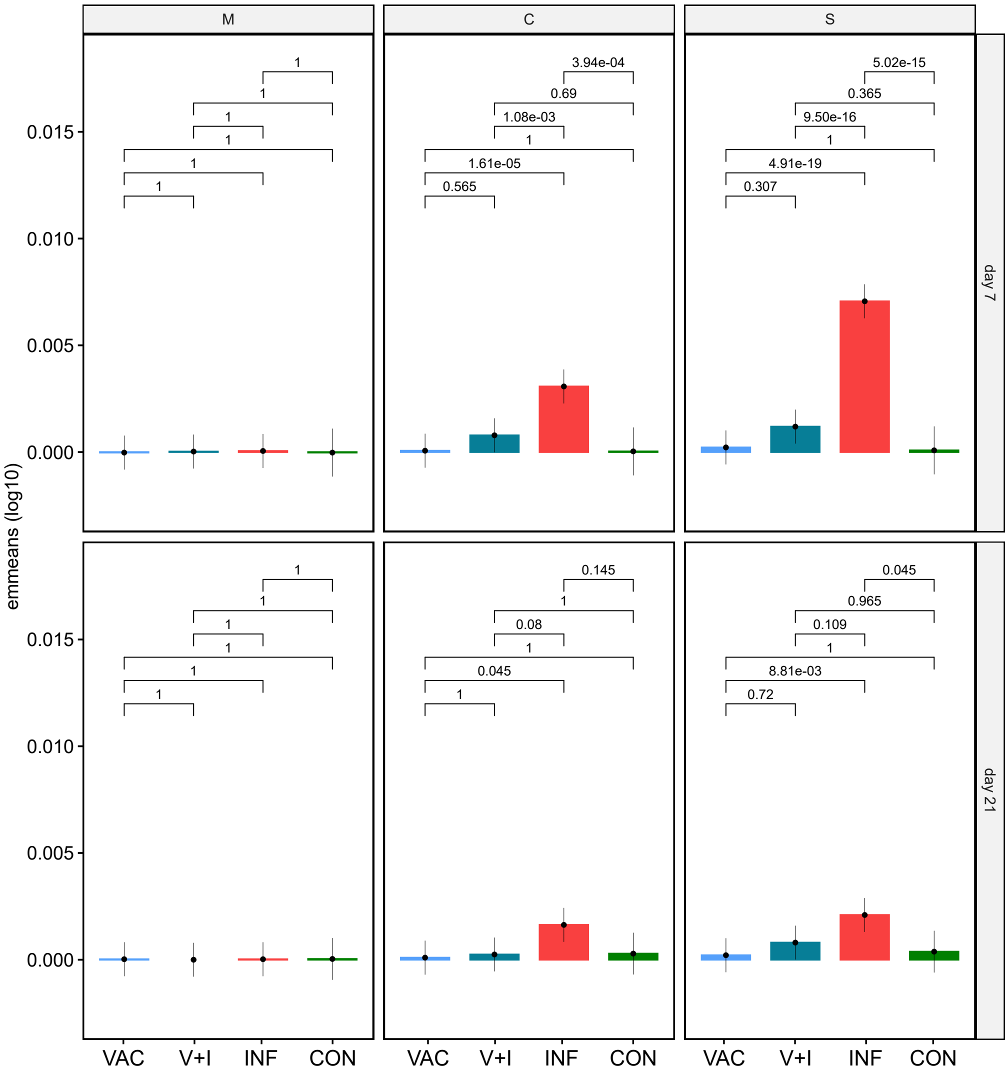

VAC
V+I
INF
CON

S: Salmoporc (vaccine strain)
C: Challenge (infection strain)
M: Medium

day 7: 7 dpv/dpi
day 21: 21 dpv/dpi

Figure S8E: CD4$^+$ T cells; Jejunum

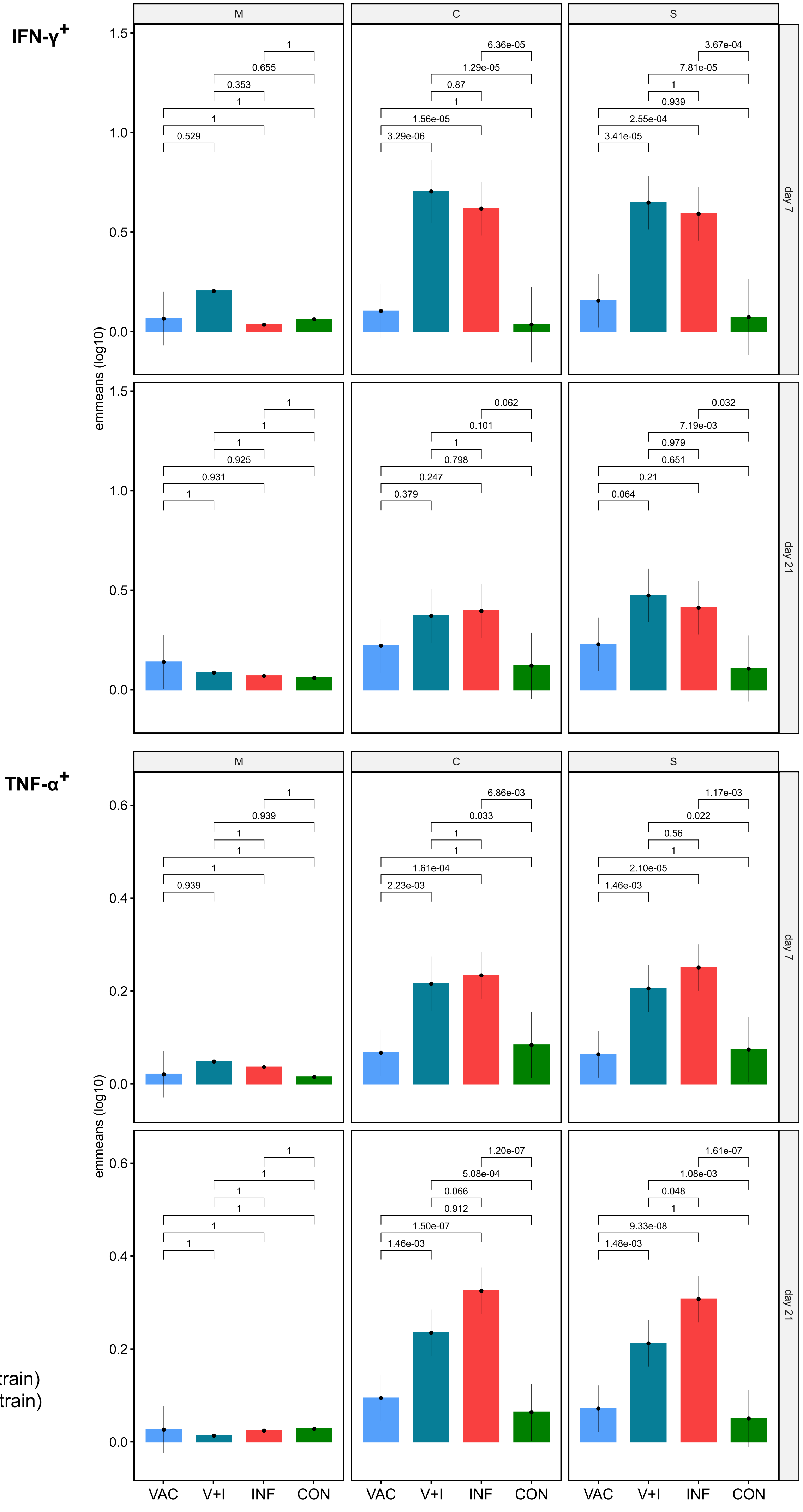

VAC
V+I
INF
CON

S: Salmoporc (vaccine strain)
C: Challenge (infection strain)
M: Medium

day 7: 7 dpv/dpi
day 21: 21 dpv/dpi

Figure S8E: CD4+ T cells; Jejunum

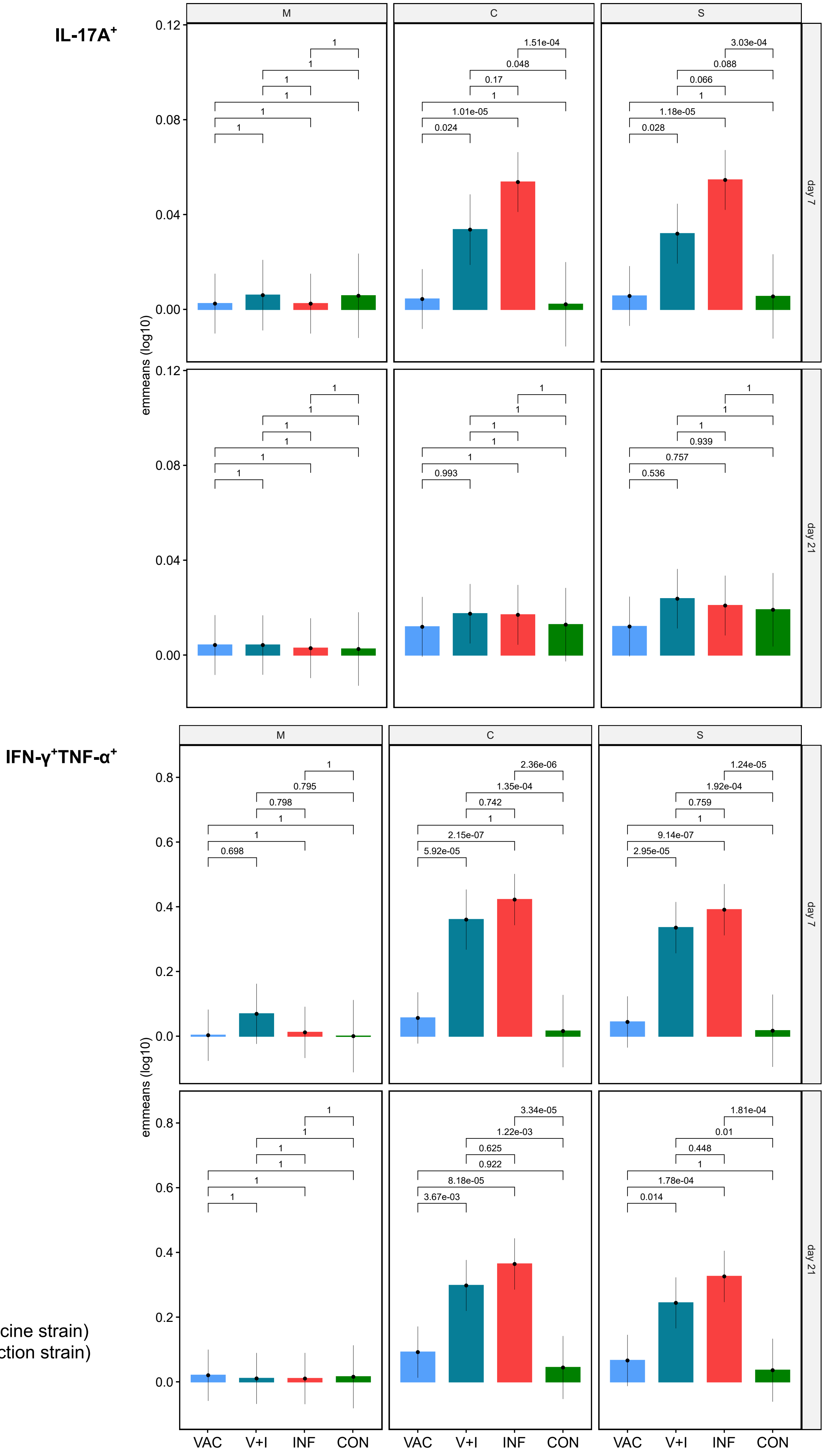

VAC
V+I
INF
CON

S: Salmoporc (vaccine strain)
C: Challenge (infection strain)
M: Medium

day 7: 7 dpv/dpi
day 21: 21 dpv/dpi

Figure S8E: CD4$^+$ T cells; Jejunum

IFN-γ$^+$IL-17A$^+$

TNF-α$^+$IL-17A$^+$

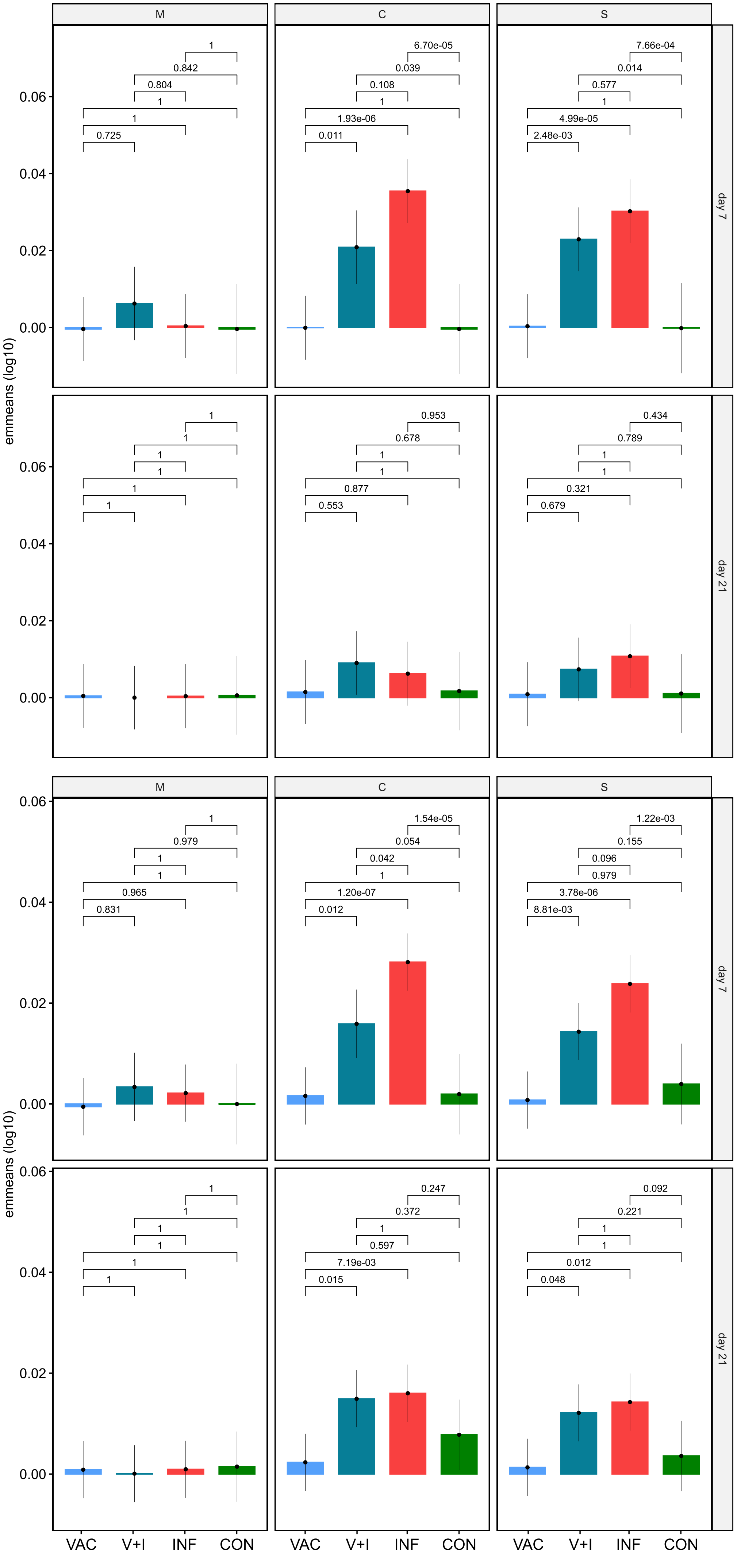

VAC
V+I
INF
CON

S: Salmoporc (vaccine strain)
C: Challenge (infection strain)
M: Medium

day 7: 7 dpv/dpi
day 21: 21 dpv/dpi

Figure S8E: CD4^{+} T cells; Jejunum

IFN-γ^{+}TNF-α^{+}IL-17A^{+}

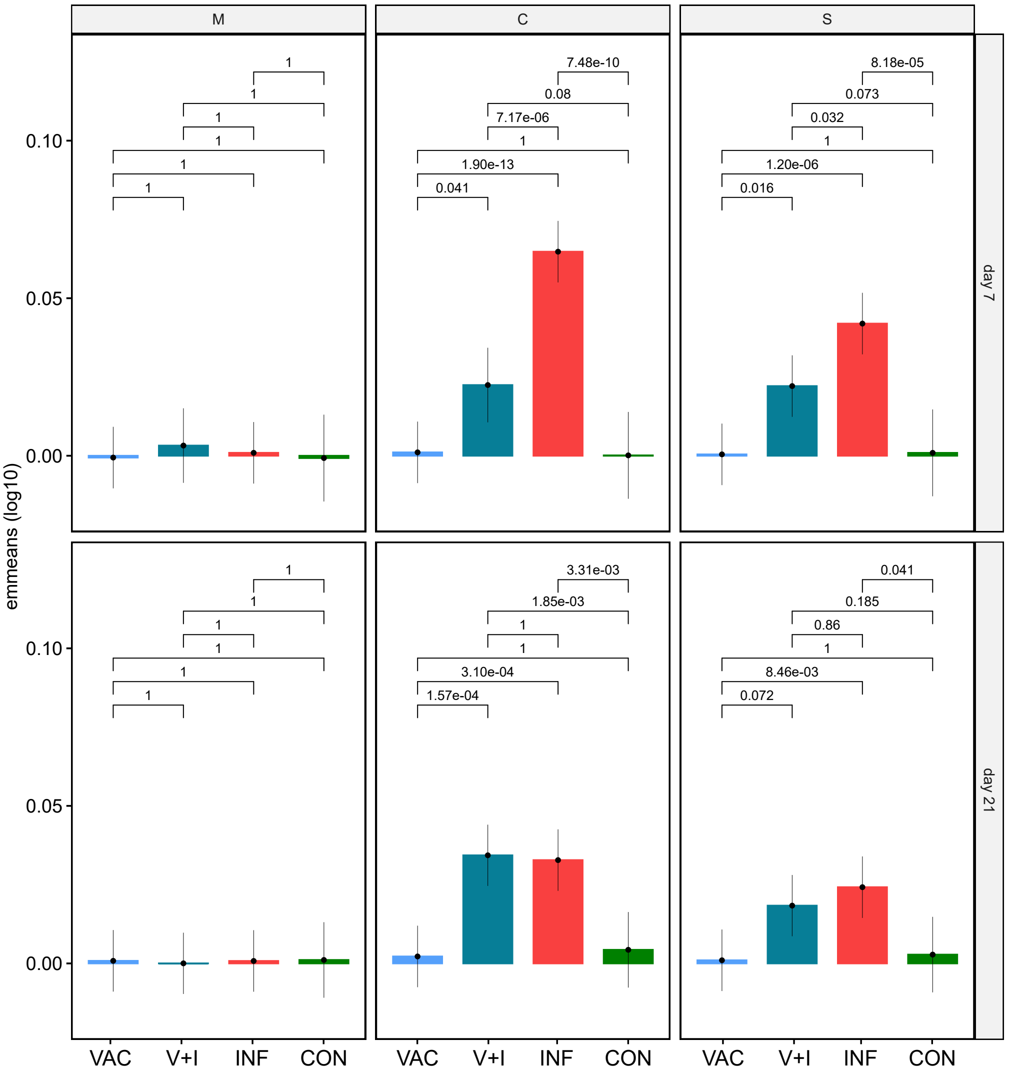

VAC
V+I
INF
CON

S: Salmoporc (vaccine strain)
C: Challenge (infection strain)
M: Medium

day 7: 7 dpv/dpi
day 21: 21 dpv/dpi

Figure S8F: CD4$^+$ T cells; Ileum

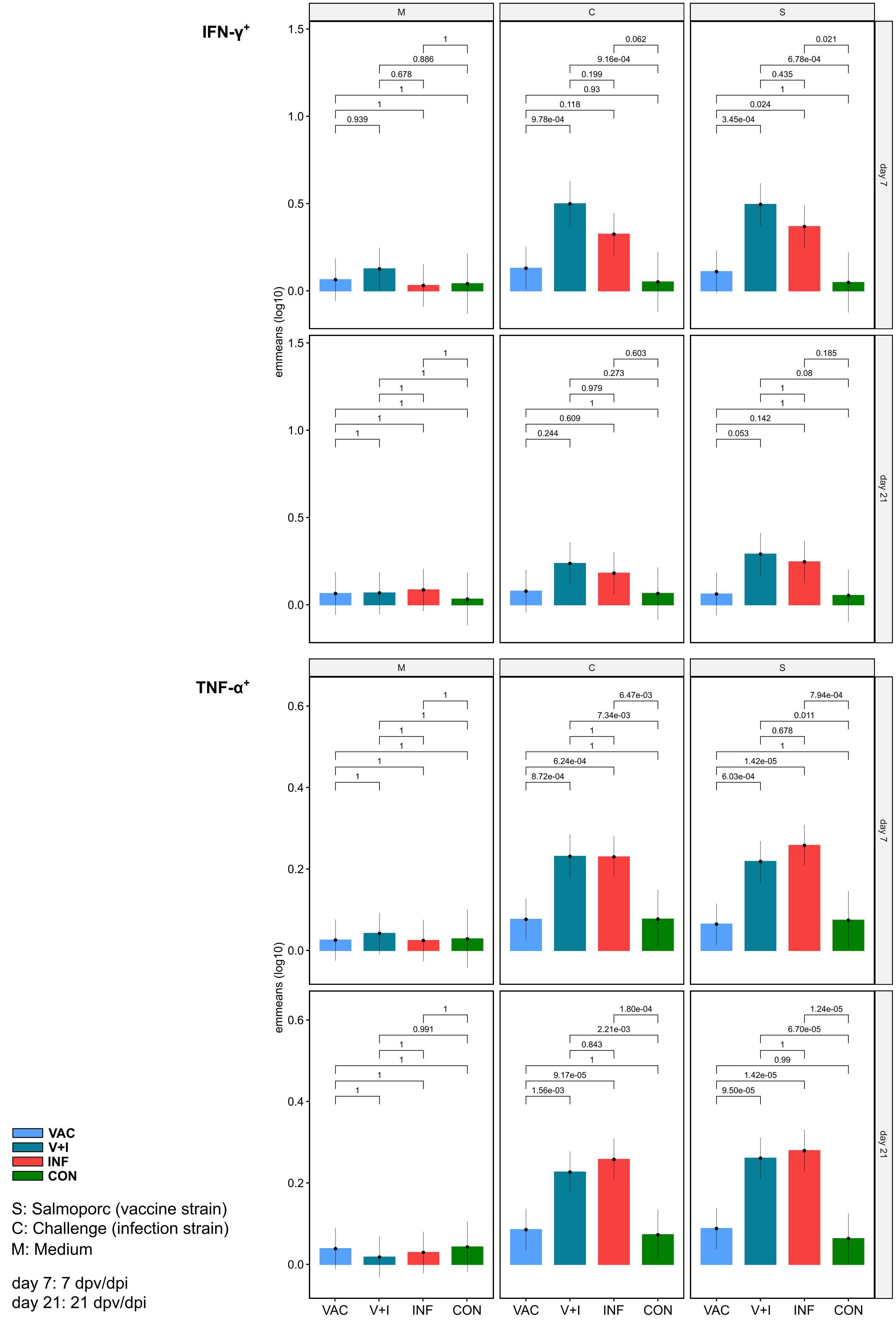

Figure S8F: CD4$^+$ T cells; Ileum

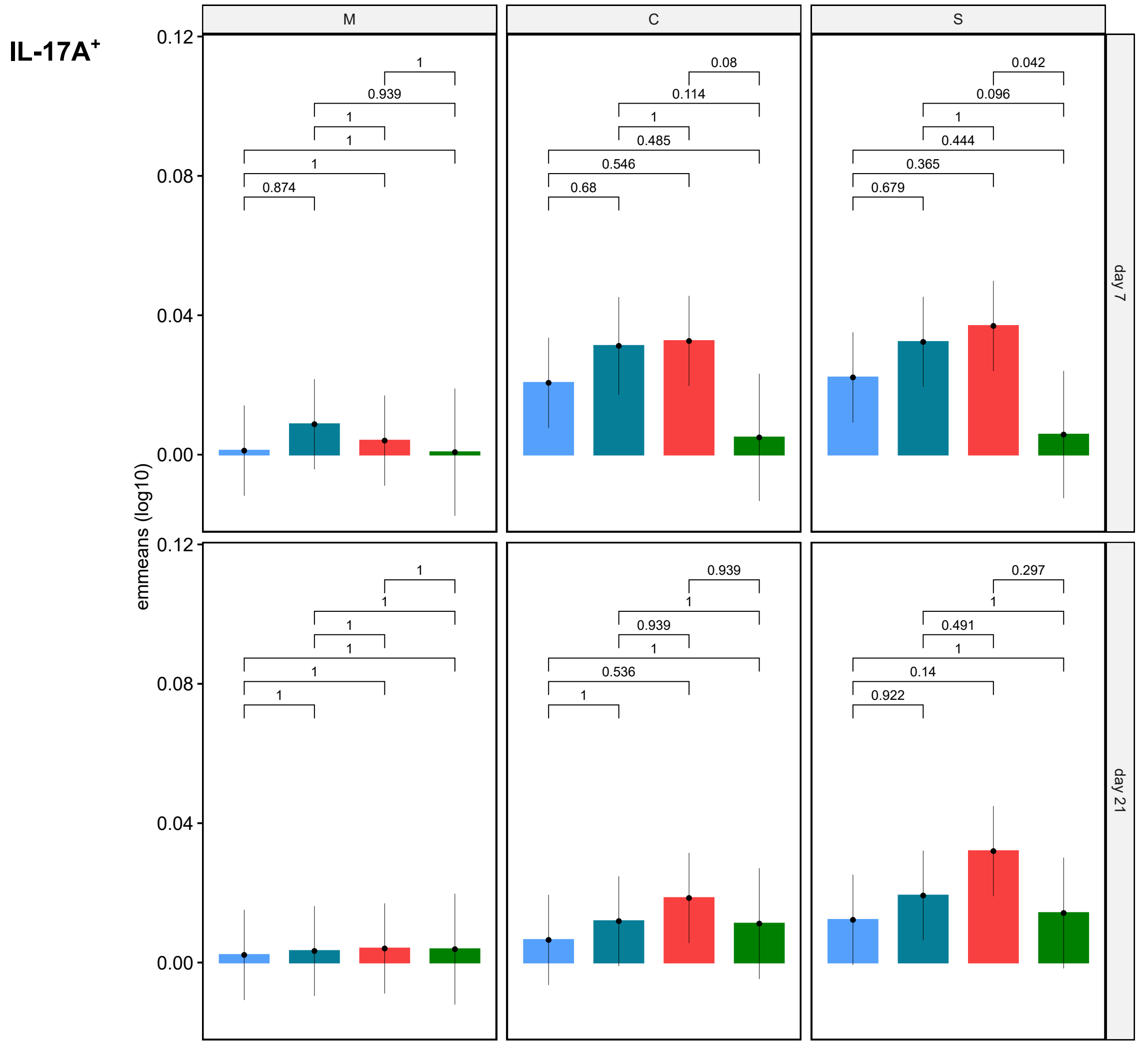

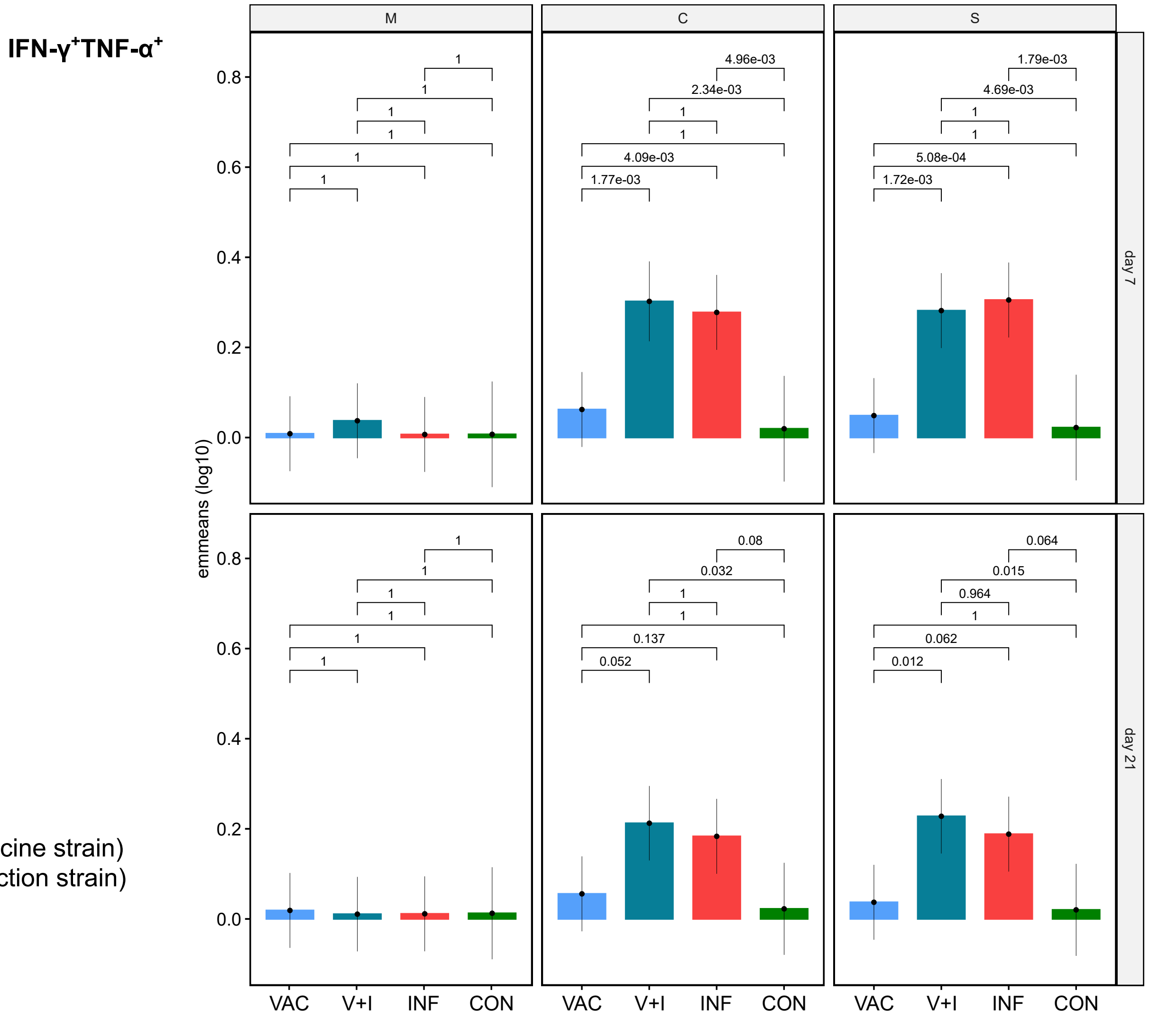

VAC
V+I
INF
CON

S: Salmoporc (vaccine strain)
C: Challenge (infection strain)
M: Medium

day 7: 7 dpv/dpi
day 21: 21 dpv/dpi

Figure S8F: CD4$^+$ T cells; Ileum

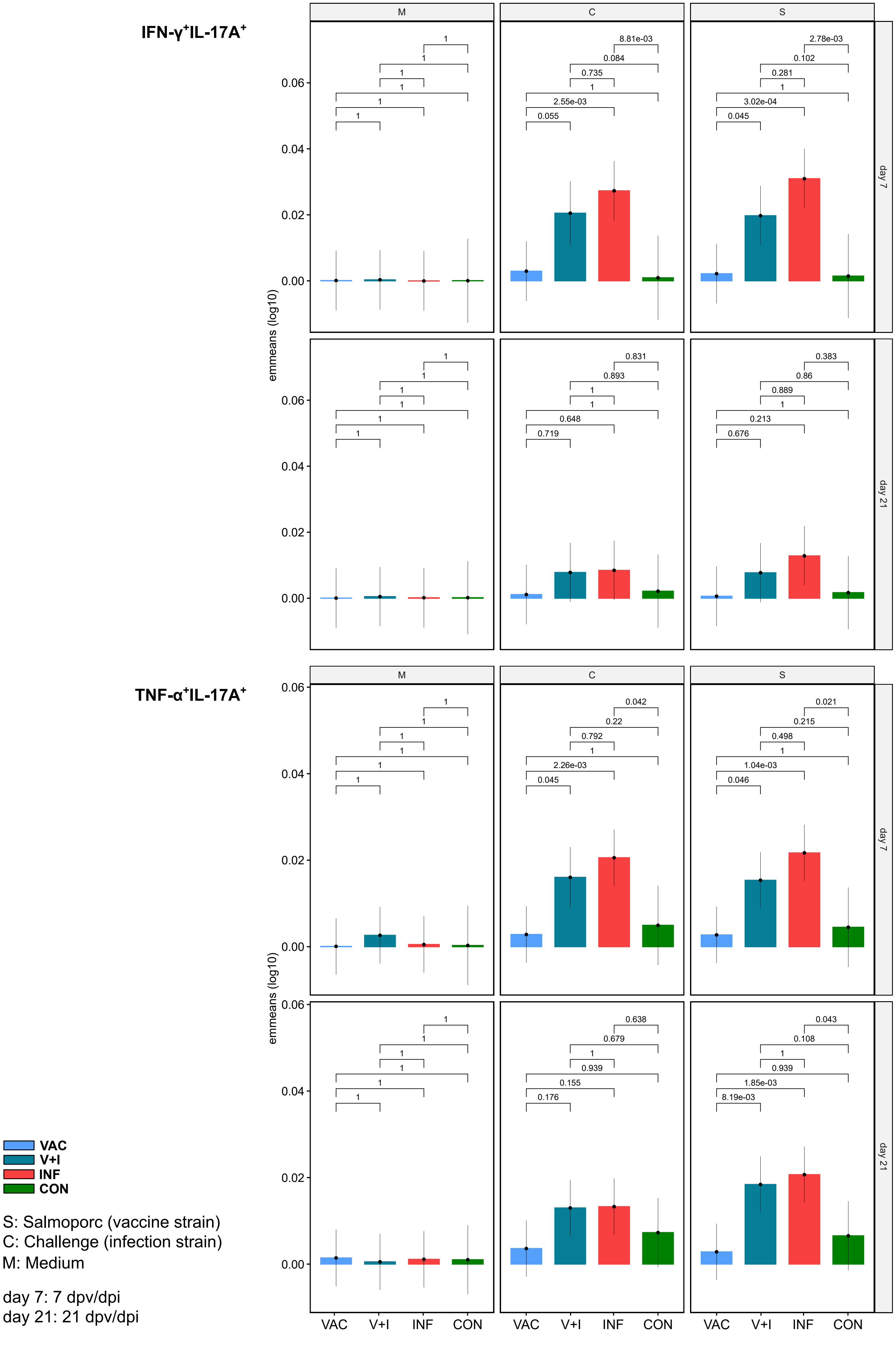

Figure S8F: CD4$^+$ T cells; Ileum

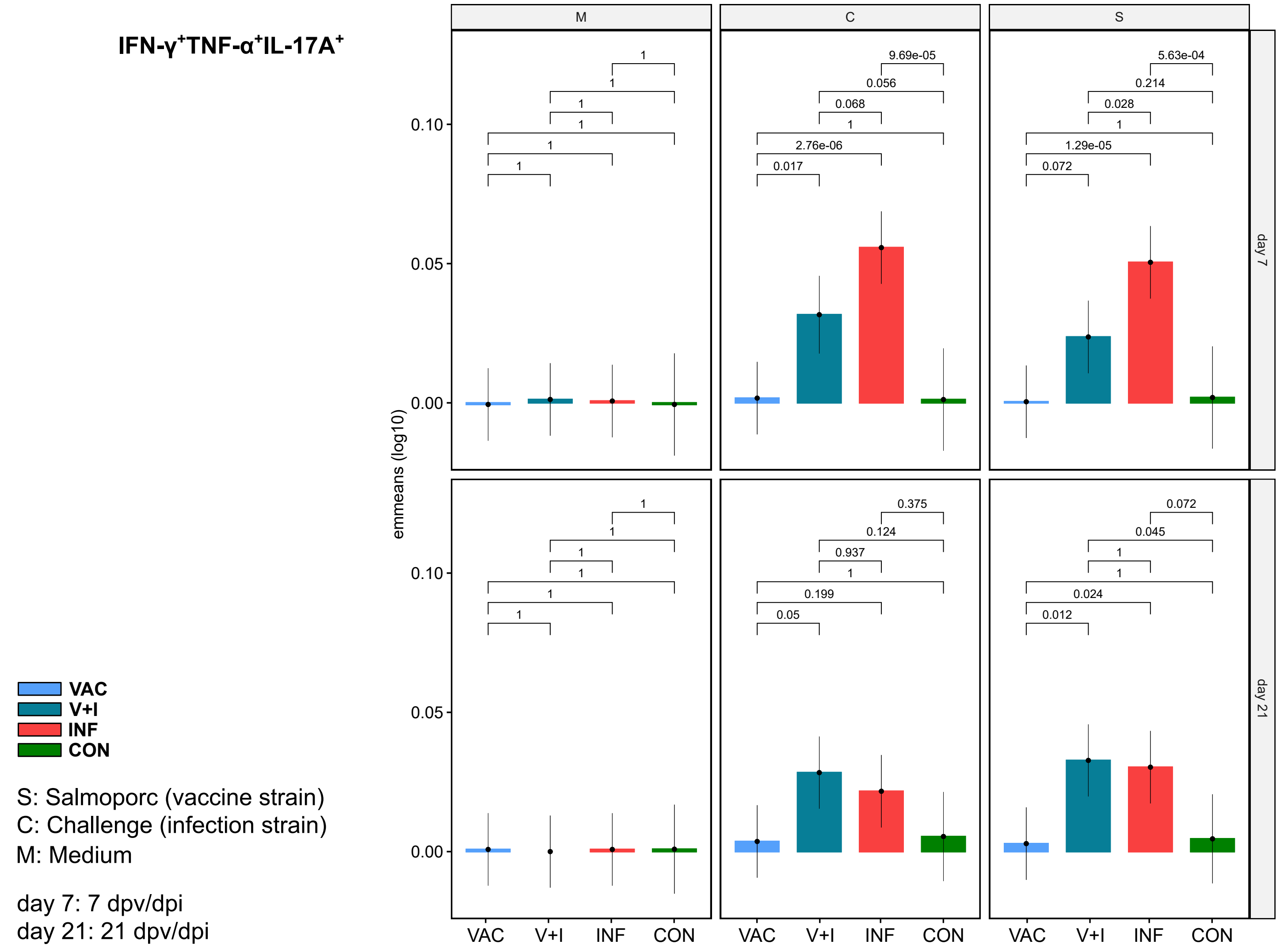

Figure S9: CD8$^+$ T cells

		VAC vs. CON			V+I vs. CON			INF vs. CON		
		IFN-γ$^+$	TNF-α$^+$	IFN-γ$^+$TNF-α$^+$	IFN-γ$^+$	TNF-α$^+$	IFN-γ$^+$TNF-α$^+$	IFN-γ$^+$	TNF-α$^+$	IFN-γ$^+$TNF-α$^+$
7 dpv/dpi	Blood - S									
	Blood - C									0.092
	Spleen - S									0.110
	Spleen - C							0.363		0.110
	JLN - S								0.015	0.005
	JLN - C								0.019	0.008
	ICLN - S								0.021	0.006
	ICLN - C								0.021	0.007
	Jejunum - S								0.029	0.011
	Jejunum - C									
	Ileum - S									
	Ileum - C									
21 dpv/dpi	Blood - S									
	Blood - C									
	Spleen - S									
	Spleen - C									
	JLN - S									
	JLN - C									
	ICLN - S				0.086			0.085		
	ICLN - C									
	Jejunum - S									
	Jejunum - C									
	Ileum - S									
	Ileum - C									

		VAC vs. V+I			VAC vs. INF			V+I vs. INF		
		IFN-γ$^+$	TNF-α$^+$	IFN-γ$^+$TNF-α$^+$	IFN-γ$^+$	TNF-α$^+$	IFN-γ$^+$TNF-α$^+$	IFN-γ$^+$	TNF-α$^+$	IFN-γ$^+$TNF-α$^+$
7 dpv/dpi	Blood - S									
	Blood - C					-0.067	-0.075			-0.065
	Spleen - S						-0.082		-0.090	-0.095
	Spleen - C				-0.251		-0.077	-0.287	-0.090	-0.094
	JLN - S					-0.012				
	JLN - C					-0.015	-0.005		-0.014	-0.007
	ICLN - S					-0.015	-0.005			-0.004
	ICLN - C					-0.015	-0.006			-0.006
	Jejunum - S					-0.024	-0.010			
	Jejunum - C			-0.008						
	Ileum - S									
	Ileum - C									
21 dpv/dpi	Blood - S									
	Blood - C		-0.071						0.067	
	Spleen - S									
	Spleen - C									
	JLN - S	-0.130			-0.105					
	JLN - C									
	ICLN - S	-0.100			-0.099					
	ICLN - C									
	Jejunum - S									
	Jejunum - C									
	Ileum - S									
	Ileum - C									

Figure S10: CD4$^-$CD8β$^-$ T cells

VAC vs. CON

		IFN-γ$^+$	TNF-α$^+$	IL-17A$^+$	IFN-γ$^+$TNF-α$^+$	IFN-γ$^+$IL-17A$^+$	TNF-α$^+$IL-17A$^+$	IFN-γ$^+$TNF-α$^+$IL-17A$^+$
7 dpv/dpi	Blood - S					3x10^{-4}		
	Blood - C							
	Spleen - S							
	Spleen - C							
	JLN - S							
	JLN - C							
	ICLN - S							
	ICLN - C							
	Jejunum - S							
	Jejunum - C							
	Ileum - S	-0.161						
	Ileum - C							
21 dpv/dpi	Blood - S							
	Blood - C							
	Spleen - S							
	Spleen - C	0.108						
	JLN - S							
	JLN - C							
	ICLN - S							
	ICLN - C							
	Jejunum - S							
	Jejunum - C							
	Ileum - S							
	Ileum - C							

V+I vs. CON

		IFN-γ$^+$	TNF-α$^+$	IL-17A$^+$	IFN-γ$^+$TNF-α$^+$	IFN-γ$^+$IL-17A$^+$	TNF-α$^+$IL-17A$^+$	IFN-γ$^+$TNF-α$^+$IL-17A$^+$
7 dpv/dpi	Blood - S							
	Blood - C							
	Spleen - S							
	Spleen - C							
	JLN - S							
	JLN - C							
	ICLN - S							
	ICLN - C							
	Jejunum - S							0.003
	Jejunum - C							
	Ileum - S							0.005
	Ileum - C							
21 dpv/dpi	Blood - S							
	Blood - C	0.081			0.077			
	Spleen - S				0.057			
	Spleen - C				0.069			
	JLN - S							
	JLN - C							
	ICLN - S							
	ICLN - C							
	Jejunum - S			0.101				
	Jejunum - C			0.112				
	Ileum - S			0.082				
	Ileum - C							

INF vs. CON

		IFN-γ$^+$	TNF-α$^+$	IL-17A$^+$	IFN-γ$^+$TNF-α$^+$	IFN-γ$^+$IL-17A$^+$	TNF-α$^+$IL-17A$^+$	IFN-γ$^+$TNF-α$^+$IL-17A$^+$
7 dpv/dpi	Blood - S							
	Blood - C	0.089	0.109		0.129			
	Spleen - S	0.173			0.114			
	Spleen - C	0.242			0.123	3x10^{-4}		
	JLN - S				0.057			0.003
	JLN - C				0.078			
	ICLN - S		0.149		0.069		0.002	0.003
	ICLN - C		0.181		0.086			0.002
	Jejunum - S							
	Jejunum - C							
	Ileum - S							
	Ileum - C							
21 dpv/dpi	Blood - S							
	Blood - C	0.083			0.085			
	Spleen - S							
	Spleen - C				0.070			
	JLN - S				0.072			
	JLN - C				0.066			
	ICLN - S							
	ICLN - C							
	Jejunum - S		0.188					
	Jejunum - C		0.150					
	Ileum - S			0.067				
	Ileum - C							

VAC vs. V+I

		IFN-γ$^+$	TNF-α$^+$	IL-17A$^+$	IFN-γ$^+$TNF-α$^+$	IFN-γ$^+$IL-17A$^+$	TNF-α$^+$IL-17A$^+$	IFN-γ$^+$TNF-α$^+$IL-17A$^+$
7 dpv/dpi	Blood - S			0.008		3x10^{-4}	0.003	
	Blood - C							
	Spleen - S			0.008				
	Spleen - C			0.008			0.001	
	JLN - S			0.017				
	JLN - C			0.013				
	ICLN - S			0.010				
	ICLN - C			0.009				
	Jejunum - S							-0.004
	Jejunum - C							
	Ileum - S					-0.013		-0.004
	Ileum - C							-0.004
21 dpv/dpi	Blood - S							
	Blood - C				-0.048			
	Spleen - S							
	Spleen - C							
	JLN - S	-0.065						
	JLN - C							
	ICLN - S							
	ICLN - C							
	Jejunum - S			-0.106				
	Jejunum - C			-0.120		-0.027		
	Ileum - S		-0.084	-0.084				
	Ileum - C							

VAC vs. INF

		IFN-γ$^+$	TNF-α$^+$	IL-17A$^+$	IFN-γ$^+$TNF-α$^+$	IFN-γ$^+$IL-17A$^+$	TNF-α$^+$IL-17A$^+$	IFN-γ$^+$TNF-α$^+$IL-17A$^+$
7 dpv/dpi	Blood - S					2x10^{-4}		
	Blood - C		-0.094		-0.102			
	Spleen - S	-0.157			-0.086			
	Spleen - C	-0.188		0.005	-0.100			
	JLN - S			0.015	-0.052		-0.001	-0.003
	JLN - C			0.012	-0.069			-0.001
	ICLN - S		-0.101	0.007	-0.059	-0.001		-0.002
	ICLN - C		-0.126		-0.072			-0.001
	Jejunum - S				-0.065			
	Jejunum - C							
	Ileum - S							
	Ileum - C							
21 dpv/dpi	Blood - S							
	Blood - C				-0.056			
	Spleen - S							
	Spleen - C							
	JLN - S	-0.078	-0.148		-0.068			
	JLN - C		-0.145		-0.054			
	ICLN - S	-0.055						
	ICLN - C							
	Jejunum - S		-0.173					
	Jejunum - C		-0.132					
	Ileum - S		-0.098	-0.069				
	Ileum - C		-0.087					

V+I vs. INF

		IFN-γ$^+$	TNF-α$^+$	IL-17A$^+$	IFN-γ$^+$TNF-α$^+$	IFN-γ$^+$IL-17A$^+$	TNF-α$^+$IL-17A$^+$	IFN-γ$^+$TNF-α$^+$IL-17A$^+$
7 dpv/dpi	Blood - S							
	Blood - C		-0.084		-0.092			
	Spleen - S	-0.138			-0.105			
	Spleen - C	-0.171			-0.114	-3x10^{-4}		-4x10^{-4}
	JLN - S							-0.003
	JLN - C		-0.128		-0.055			
	ICLN - S		-0.128		-0.052	-0.001		-0.002
	ICLN - C		-0.160		-0.062			-0.001
	Jejunum - S							
	Jejunum - C							
	Ileum - S					0.012		0.004
	Ileum - C							
21 dpv/dpi	Blood - S							
	Blood - C							
	Spleen - S							
	Spleen - C							
	JLN - S							
	JLN - C							
	ICLN - S							
	ICLN - C							
	Jejunum - S							
	Jejunum - C							
	Ileum - S							
	Ileum - C							
